# A Bayesian Multilevel Joint Modeling of Longitudinal and Survival Outcomes in Cluster Randomized Controlled Trial Studies

**DOI:** 10.1002/sim.70385

**Published:** 2026-01-22

**Authors:** Yixiu Liu, Depeng Jiang, Mahmoud Torabi, Xuekui Zhang

**Affiliations:** ^1^ College of Community and Global Health University of Manitoba Winnipeg Manitoba Canada; ^2^ Department of Mathematics and Statistics University of Victoria Victoria British Columbia Canada

**Keywords:** Bayesian, cluster randomized controlled trail, group‐based intervention/treatment, multilevel joint model of longitudinal and survival data

## Abstract

Cluster randomized controlled trials (CRCTs) are commonly used when interventions are delivered at the group level. Since data from CTCTs are inherently multilevel, methods that properly account for clustering are required. Joint modeling (JM) of longitudinal and survival data allows for simultaneous evaluation of intervention effects on repeated measures and time‐to‐event outcomes, offering a comprehensive view of intervention effects. However, existing JMs do not accommodate clustered data structures typically of CRCTs. This study introduces a multilevel joint model (MJM) to simultaneously evaluate intervention effects on correlated longitudinal and survival outcomes. The model was applied to empirical data from a large CRCT evaluating the PAX Good Behavior Game, a classroom‐based mental health intervention involving 4189 Grade 1 students across 313 classrooms during the 2011–2012 school year. Mental health was assessed at three time points: pre‐PAX (January 2012), post‐PAX (June 2012), and Grade 5 (June 2016). Time‐to‐first mental disorder diagnosis was tracked through March 2024. Simulation studies further evaluated the MJM's performance under varying conditions, including censoring rates, cluster sizes, group‐level variances, and survival model specifications. Results indicated the PAX program significantly improved mental health trajectories and reduced the risk of mental disorder diagnoses. The MJM outperformed traditional JMs by producing more accurate estimates and standard errors. Both empirical and simulation findings demonstrated that ignoring hierarchical structures leads to biased inferences and underestimation of intervention effects. The proposed MJM offers a robust and flexible analytic framework for analyzing data from CRCTs, emphasizing the importance of accounting for clustering in evaluating group‐based interventions.

## 1 Introduction

Mental health concerns among children and youth have become an urgent global public health priority. The World Health Organization reported that one in seven individuals aged 10–19 worldwide experienced a mental disorder, most commonly anxiety, depression, and behavioral disorders [1]. The COVID‐19 pandemic has exacerbated this crisis, because of disrupting schooling, increasing social isolation, and growing stress within families. Studies have reported rising levels of emotional distress, behavioral challenges, and a greater demand for mental health services among children during and following the pandemic [2, 3]. As societies continue to recover, prioritizing children's mental health is essential in post‐pandemic public health planning.

There is strong evidence that mental health trajectories are shaped early in life. Childhood is a critical developmental period for emotional regulation, social skills, and coping mechanisms. Mental health difficulties that emerge early often persist or intensify over time, leading to adverse outcomes such as school dropout, substance use, and chronic mental illness in later life [4, 5]. Conversely, early interventions promoting resilience and well‐being can provide enduring protective benefits. Preventive strategies targeting the social and emotional development of children within natural settings, especially schools, are therefore critical.

School‐based mental health interventions have the advantage of efficiently reaching many children, reducing stigma, and fostering supportive classroom environments. The PAX Good Behavior Game (PAX), an evidence‐based intervention aimed at enhancing children's behavioral self‐regulation and peer interactions, exemplifies this approach. In Manitoba, Canada, the PAX program was implemented province‐wide for Grade 1 students during the 2011/2012 school year to improve children's mental well‐being [6]. To evaluate its effectiveness, a cluster randomized controlled trial (CRCT) was conducted, with participating schools randomly assigned either to implement the PAX program (PAX group) or to continue standard classroom practices (control group).

Children's mental health was assessed at two initial time points: before (pre‐PAX) and after (post‐PAX) the implementation of the PAX program. Analyses using these data demonstrated the immediate effectiveness of PAX in improving children's mental health [6, 7]. Subsequently, the Government of Manitoba conducted a provincial‐wide assessment of Grade‐5 children's mental health using the same instrument as the PAX study, allowing longitudinal tracking of most participants from the initial cohort. Analysis using data from the three timepoints confirmed the PAX program's effect in reducing the risk of mental disorders, which was defined using a validated cutoff for the measure of mental health [8].

Furthermore, data from the PAX CRCT cohort can be linked to provincial administrative databases to track clinical mental disorder diagnoses. An earlier provincial report, based on the data up to March 31, 2015, found no significant differences in mental disorder diagnoses between children in the PAX and control groups, and authors called for future evaluation of long‐term follow‐up on the effect of the PAX program. Given the increasing mental health challenges post‐pandemic, assessing the sustained effectiveness of the PAX is timely and essential. Understanding how the PAX program influences developmental trajectories and prevents clinically significant mental health problems could significantly inform policy decisions and optimize the allocation of mental health services.

To comprehensively evaluate the effectiveness of the PAX program, it is important to consider both longitudinal measures of mental health and clinical diagnoses of mental disorders. The joint model for longitudinal and survival data (JM), which allows analyzing the two outcomes simultaneously, is the most appropriate method to use [9, 10]. However, the classrooms as the unit of randomization and the implementation of the intervention create challenges when applying JMs. The PAX data exhibits a hierarchical data structure, with children nested within classrooms, yet there have been limited discussions on JMs that can properly account for such multilevel data.

Existing research on JMs for multilevel data has several limitations. Some studies overlooked group‐level factors [11], while others focused on different multilevel data structures, such as repeated measurements within lesions nested inside patients [12]. Recent developments in multilevel joint models (MJMs) have incorporated multilevel structures and allowed time‐varying effects of the longitudinal outcome on the survival outcome but have not included group‐level random effects in the survival submodel [13, 14]. Another recent spatiotemporal MJM adopted shared random effects to link the longitudinal and survival submodels rather than directly including the longitudinal outcome as a predictor in the survival model [15]. Importantly, existing MJMs have not addressed interactions between time and group‐level factors, limiting the evaluation of group‐based interventions like PAX.

One possible reason for the limited research on MJMs is their complexity and high computational burden. The Bayesian approach, particularly using Just another Gibbs Sampler (JAGS), has become a popular method for fitting JMs and was used in abovementioned research on MJMs for multilevel data. However, it is well known that there are two computationally intensive procedures—the “zero‐trick” and the numeric integration—to approximate the survival function when using JAGS to fit JM with proportional hazards (PH) model as the survival submodel [16]. An alternative approach, the auxiliary Poisson model, avoids these complexities and has proven effective in standard JMs [17]. For multilevel survival data, statistical theory and simulation studies have demonstrated that the auxiliary mixed‐effect Poisson model produces equivalent estimates to the piecewise constant proportional hazards (PCPH) model with random effects [18, 19, 20]. Moreover, using the auxiliary mixed‐effect Poisson approach for nested survival data offers significant computational efficiency [21]. Therefore, adopting the auxiliary mixed‐effect Poisson approach for modeling the survival outcome in MJMs could potentially reduce computational demands and enable the development of more flexible MJMs.

The purpose of this study is to propose a Bayesian multilevel joint model (MJM) for analyzing nested longitudinal and survival data. The longitudinal submodel utilizes a three‐level linear mixed‐effects model (LMM), while the survival submodel employs the auxiliary mixed‐effect Poisson regression with group‐level random effects.

The remainder of this manuscript is organized as follows: In Section 2, the model formulation is described. Bayesian inference of our proposed MJM is discussed in Section 3. Section 4 applies the proposed model to the PAX study. Section 5 outlines the design of simulation studies to assess the performance of the proposed MJM under various conditions and investigates the impact of disregarding the nested data structure. Finally, we summarize the findings of this study in Section 6.

## 2 Model Formulation

### 2.1 Longitudinal Submodel

In this study, we focus on a single continuous three‐level longitudinal outcome. Repeated measurements (level 1) are nested within individuals (level 2), who are further nested within groups (level 3). Specifically, let $y_{\mathrm{ijl}}\left(t_{\mathrm{ijl}}\right)$ represent the longitudinal outcomes measured at time $t_{\mathrm{ijl}}\;\left(i=1,\ldots ,n_{\mathrm{lj}}\right)$ for subject j(j=1,…,n), who belongs to the group l(l=1,…,L). We model this longitudinal outcome using a three‐level linear mixed‐effect model (LMM).

(1) $$\begin{array}{cc} & y_{\mathrm{jl}}\left(t_{\mathrm{ijl}}\right)=m_{\mathrm{jl}}\left(t_{\mathrm{ijl}}\right)+\varepsilon_{\mathrm{jl}}\left(t\right),\\ & m_{\mathrm{jl}}=X_{\mathrm{jl}}^{T}\beta +Z_{\mathrm{jl}}^{T}u_{\mathrm{jl}}+{\mathrm{WL}}_{l}^{T}v_{l},\\ & u_{\mathrm{jl}}∼N\left(0,\sum_{u}\right),v_{l}∼N\left(0,\sum_{v}\right),\varepsilon_{\mathrm{jl}}\left(t\right)∼N\left(0,\sigma_{\varepsilon }^{2}\right), \end{array}$$

where for simplicity, $y_{\mathrm{jl}}\left(t_{\mathrm{ijl}}\right)$ indicates the $y_{\mathrm{ijl}}\left(t_{\mathrm{ijl}}\right)$; $m_{\mathrm{jl}}\left(t_{\mathrm{ijl}}\right)$ indicates the linear predictor at time $t_{\mathrm{ijl}}$ for subject j from group l; $X_{\mathrm{jl}}^{T}\left(t\right)$ is the design matrix for covariates associated with the fixed effect parameter β, which may include time‐varying variables at the first level, variables at the individual level and group level, as well as interaction terms among the variables across all three levels. $Z_{\mathrm{jl}}^{T}$ is the design matrix for the subject‐level random‐effect $u_{\mathrm{jl}}$, which commonly consists of two columns, with the first column being one to indicate the intercept and the second column being time; ${\mathrm{WL}}_{l}^{T}$ is the design matrix for the group‐level random effects $v_{l}$; The first column of each design matrix consists of ones to indicate the intercept; $\varepsilon_{\mathrm{jl}}\left(t\right)$ is the level‐1 residual, also called measurement error. We assume $\varepsilon_{\mathrm{jl}}\left(t\right)$ follows a normal distribution $N\left(0,\sigma_{\varepsilon }^{2}\right)$ while the subject‐level random effects $u_{\mathrm{jl}}$ and group‐level random effects $v_{l}$ follow a multivariate normal distribution with variance–covariance matrix $\sum_{u}$ and $\sum_{v}$, respectively. The random effects at different levels and the level 1 residual are assumed to be mutually uncorrelated.

### 2.2 Survival Submodel

The n subjects are assumed to be followed from time 0 to time T. Let $T_{\mathrm{jl}}^{*}$ denotes the true event time for subject j, which is nested in group l. The observed event time $T_{\mathrm{jl}}$ equals $\min\left\{T_{\mathrm{jl}}^{*},C_{\mathrm{jl}}\right\}$, where $C_{\mathrm{jl}}$ is the right‐censoring time. Let $\delta_{\mathrm{jl}}=I\left(T_{\mathrm{jl}}^{*}\leq C_{\mathrm{jl}}\right)$be the event indicator, where I(·) is an indication function. The proportional hazards model has the form.

(2) $$\lambda_{\mathrm{jl}}\left(t\right)=\lambda_{0}\exp\left\{\omega_{\mathrm{jl}}^{T}\left(t\right)\gamma +f\left\{M_{\mathrm{jl}}\left(t\right),u_{\mathrm{jl}},\alpha \right\}+{\mathrm{WS}}_{l}^{T}g_{l}\right\},$$

where $\lambda_{0}$ indicates the baseline hazard function and γ is a vector of fixed‐effect parameters for covariates $\omega_{\mathrm{jl}}$. The fixed‐effect covariates $\omega_{\mathrm{jl}}$ can contain the same or partly the same fixed‐effect covariates in the longitudinal submodel. $M_{\mathrm{jl}}\left(t\right)=\left\{m_{\mathrm{jl}}\left(s\right),0\leq s<t\right\}$ indicates the history of the repeated measurements up to time t. The parameter α indicates the strength of the association between features of the longitudinal outcomes and the hazard function. f(·) specifies the association structure between the longitudinal and survival outcomes. The $g_{l}$ is the random effects at group level for the covariate vector ${\mathrm{WS}}_{l}$. Similarly, the first element of ${\mathrm{WS}}_{l}$ is one to indicate intercept. We assume a multivariate normal distribution for the random effects, $g_{l}∼N\left(0,\sum_{g}\right)$.

The baseline hazard function needs to be specified in JMs to get accurate estimation of standard errors [22]. The piecewise constant has been shown to have good performance and has been widely adopted in the development of JMs [23, 24, 25, 26, 27, 28, 29]. Additionally, using the piecewise constant proportional hazards (PCPH) model allows the adoption of the auxiliary mixed‐effects Poisson model in the survival submodel in JMs [17].

When using PCPH model for survival data, the follow‐up period is divided into Q time intervals, $\left[\tau_{q-1},\tau_{q}\right),q=1,\ldots ,Q$, where $0=\tau_{0},\tau_{1}$,…, $\tau_{Q}=\infty$. Then, the hazard function takes the form:

(3) $$\begin{array}{ll} \lambda_{\mathrm{jlq}}\left(t\right) & =\lambda_{q}\exp\left\{\omega_{\mathrm{jlq}}^{T}\left(t\right)\gamma +f\left\{M_{\mathrm{jlq}}\left(t\right),u_{\mathrm{jl}},\alpha \right\}+{\mathrm{WS}}_{l}^{T}g_{l}\right\},\\ & t\in \left[\tau_{q-1},\tau_{q}\right), \end{array}$$

where the baseline hazard in the time interval $\left[\tau_{q-1},\tau_{q}\right)$ is assumed to be a constant $\lambda_{q}$. For each subject, we generate pseudo‐observations $\delta_{\mathrm{jlq}},q=1,\ldots ,Q,$ to use the auxiliary mixed‐effect Poisson modeling approach. These pseudo‐observation $d_{\mathrm{liq}}$ indicate whether the event occurs in interval q, for subject j, who is nested in group l. The occurrence of the event follows a Poisson distribution with random effects,

$$\delta_{\mathrm{jlq}}∼\text{Poisson}\left(\mu_{\mathrm{jlq}}\right),q=1,\ldots ,Q.$$

Using the GLMM with the log link function, the mean, $\mu_{\mathrm{jlq}}$, is modeled as follows, where the exposure time in the interval q of subject j nested in group l is treated as an offset,

**Table:** 

$\log\left(\mu_{\mathrm{jlq}}\right)=\log t_{\mathrm{jlq}}+\log\lambda_{q}+\omega_{\mathrm{jlq}}^{T}\left(t\right)\gamma +f\left\{M_{\mathrm{jlq}}\left(t\right),u_{\mathrm{jl}},\alpha \right\}+{\mathrm{WS}}_{l}^{T}g_{l}$
$\left\{\begin{array}{l} t_{\mathrm{liq}}=\tau_{q}-\tau_{q-1},ifeventhasnotoccurred\\ t_{\mathrm{jlq}}=T_{\mathrm{jl}}-\tau_{q-1},ifeventoccurredin[\tau_{q-1},\tau_{q}) \end{array}\right.$

In survival analysis, the parameter estimates from the auxiliary mixed‐effects Poisson regression and the PCPH model with random effects are theoretically identical [19, 30]. Furthermore, estimates from a PCPH model with random effects are theoretically identical to those from a mixed‐effects PH model with an unspecified baseline hazard function (i.e., mixed‐effects Cox model) when the number of time intervals used in the data splitting equals the number of events in the study [19]. However, generating such a large number of pseudo‐observations through the auxiliary mixed‐effect Poisson model to achieve identical estimates to the mixed‐effects Cox model would impose unrealistic computational demands, particularly when the sample size and/or the number of events is large.

Fortunately, it has been shown that the auxiliary mixed‐effect Poisson regression approach can provide estimates that closely approximate those from the mixed‐effects Cox model even when the follow‐up time is split into a few time intervals [21, 30, 31]. In practice, it is common to split the follow‐up time into equal‐length time intervals such as yearly intervals, which often align with the data collection schedule of time‐dependent covariates [21]. Alternatively, the length of the time interval can be determined based on clinical relevance [31]. Within the JM framework, splitting the follow‐up period into intervals consistent with the longitudinal data collection schedule has been shown to yield satisfactory results [17].

We propose to use the auxiliary mixed‐effect Poisson model as the survival submodel in the proposed multilevel joint model of nested longitudinal and time‐to‐event data. The random effects $g_{l}$ in the mixed‐effect Poisson model is assumed to be independent of the random effects at group level in the longitudinal submodel. Adopting a generalized form in JM literature, the association structure is a function of subject‐specific predictions, random effects, and various parameterizations of the predicted longitudinal trajectory [23, 32].

## 3 Estimation and Inference

### 3.1 Bayesian Inference

We employ a Bayesian approach using Markov chain Monte Carlo (MCMC) methods to estimate the parameters of the proposed MJM. The posterior sampling was implemented in JAGS through the R packages ***rjags*** and ***coda***. The trace plots, autocorrelation function (ACF) plots, posterior density plots, and the Gelman‐Rubin‐Brooks diagnostic ($\hat{R}$) were checked to assess the convergence of MCMC chains. The trace plot shows the realization of MCMC chains at each iteration, and convergence may be indicated by overlapping chains resembling a hairy caterpillar [33]. Additionally, the trace plot was used to decide the number of burn‐in iterations, where a number of iterations before the chains become stable were discarded [34]. Three chains were conducted to check the influence of initial values on the Bayesian estimates. The number of thinning was decided according to the ACF plot to draw more independent posterior samples while saving storage [35]. Sensitivity analyses were conducted to compare the results with and without thinning. Convergence was deemed achieved when the value of Gelman–Rubin–Brooks diagnostic statistics ($\hat{R}$) is less than 1.1 for all parameters [34].

### 3.2 Prior and Likelihood Distributions

The unknown parameters that need to be estimated in the proposed MJM are indicated by $\Theta =\left(\beta ,\sigma_{\varepsilon },\sum_{u},\sum_{v},\gamma ,\alpha ,\sum_{g},\lambda \right)$. Weak priors with hyperparameters informed by the literature were adopted [36, 37, 38]. Specifically, the priors for parameters in the longitudinal submodel $(\beta ,\sigma_{\varepsilon },\sum_{u},\sum_{v}$) are:

$$\beta_{s}∼N\left(0,\frac{1}{0.001}\right),s=1,\ldots ,p_{\beta },$$

$$\sigma_{\varepsilon }∼\text{Inverse}-\text{Gamma}\left(0.01,0.01\right),$$

$$\sum_{u}∼\text{Inverse}-\text{Wishart}\left({\left[\begin{array}{lll} 1 & \cdots & 0\\ \vdots & \ddots & \vdots \\ 0 & \cdots & 1 \end{array}\right]}_{p_{u}\times p_{u}},p_{u}\right),$$

$$\sum_{v}∼\text{Inverse}-\text{Wishart}\left({\left[\begin{array}{lll} 1 & \cdots & 0\\ \vdots & \ddots & \vdots \\ 0 & \cdots & 1 \end{array}\right]}_{p_{v}\times p_{v}},p_{v}\right),$$

where $p_{u}$ and $p_{v}$ is the dimension of random effects at the subject level and group level, respectively. For parameters in the survival submodel ($\lambda_{q},\gamma ,\alpha ,\sum_{g}$), the prior distributions are:

$$\lambda_{q}∼\text{gamma}\left(0.01,0.01\right),q=1,\ldots ,Q,$$

$$\gamma_{s}∼N\left(0,\frac{1}{0.001}\right),s=1,\ldots ,p_{\gamma },$$

$$\alpha_{s}∼N\left(0,\frac{1}{0.001}\right),s=1,\ldots ,p_{\alpha },$$

$$\sum_{g}∼\text{Inverse}-\text{Wishart}\left({\left[\begin{array}{lll} 1 & \cdots & 0\\ \vdots & \ddots & \vdots \\ 0 & \cdots & 1 \end{array}\right]}_{p_{g}\times p_{g}},p_{g}\right),$$

where $p_{\gamma }$ is the dimension of covariate in the survival submodel, $p_{\alpha }$ is the dimension of the association parameters in the survival submodel, $p_{g}$ is the dimension of the random effects at the group level in the survival submodel.

Conditional on the random effects at subject level and group level in both submodels, $u_{\mathrm{jl}},v_{l},g_{l}$, and other parameters, the distributions for the longitudinal and pseudo‐Poisson outcomes are:

$$y_{\mathrm{jl}}|\;u_{\mathrm{jl}},v_{l},g_{l},\Theta ∼N\left(X_{\mathrm{jl}}^{T}\left(t\right)\beta +Z_{\mathrm{jl}}^{T}u_{\mathrm{jl}}+{\mathrm{WL}}_{l}^{T}v_{l},\sigma_{\varepsilon }^{2}\right),$$

$$\delta_{\mathrm{jlq}}|\;u_{\mathrm{jl}},v_{l},g_{l},\Theta ∼\text{Poisson}\left(\mu_{\mathrm{jlq}}\right),t_{i}\in \left[\tau_{q-1},\tau_{q}\right),\text{where},$$

(4) $$\log\left(\mu_{\mathrm{jlq}}\right)=\log t_{\mathrm{jlq}}+\log\lambda_{q}+\omega_{\mathrm{jlq}}^{T}\left(t\right)\gamma +\alpha *m_{\mathrm{jlq}}\left(t\right)+{\mathrm{WS}}_{l}^{T}g_{l}.$$

### 3.3 Posterior Distribution

We assume that conditional on all the random effects and other parameters, the longitudinal outcome and the survival outcome, and the repeated measurements are independent. Then, the posterior distribution of unknown parameters takes the form:

$$\begin{array}{ll} L\left(\Theta |\text{Data}\right) & \propto L\left(\text{Data}|\Theta \right)\cdot p\left(\Theta \right)\\ & =\prod_{l=1}^{L}\prod_{j=1}^{n}p_{s}\left(T_{\mathrm{jl}},\delta_{\mathrm{jl}}|u_{\mathrm{jl}},v_{l},g_{l},\Theta \right)\\ & \;\cdot \prod_{i=1}^{n_{\mathrm{jl}}}p_{y}\left(y_{\mathrm{ijl}}|u_{\mathrm{jl}},v_{l},g_{l},\Theta \right)\cdot p\left(u_{\mathrm{jl}}|\Theta \right)\\ & \;\cdot p\left(v_{l}|\Theta \right)\cdot p\left(g_{l}|\Theta \right)\cdot p\left(\Theta \right), \end{array}$$

where $p_{s}$ is the probability density function (pdf) for the survival outcome; $p_{y}$ is the pdf for the repeated measurements. The prior pdfs for all the parameters that need to be estimated are indicated by p(Θ).

The likelihood for the mixed‐effect PCPH model is proportional to the likelihood for the auxiliary mixed‐effect Poisson model [19, 30, 39]. Therefore, the joint likelihood function is proportional to the product of the pdfs of the longitudinal and pseudo‐Poisson outcomes. Then, the posterior distribution of the unknown parameters in the proposed MJM can be written as:

$$\begin{array}{ll} L\left(\Theta |\text{Data}\right) & \propto \prod_{l=1}^{L}\prod_{j=1}^{n}\prod_{q=1}^{q\left(j\right)}p_{s}\left(T_{\mathrm{jl}},\delta_{\mathrm{jlq}}|u_{\mathrm{li}},v_{l},g_{l},\Theta \right)\\ & \;\cdot \prod_{i=1}^{n_{\mathrm{jl}}}p_{y}\left(y_{\mathrm{ijl}}|u_{\mathrm{jl}},v_{l},g_{l},\Theta \right)\cdot p\left(u_{\mathrm{jl}}|\Theta \right)\cdot p\left(v_{l}|\Theta \right)\\ & \;\cdot p\left(g_{l}|\Theta \right)\cdot p\left(\Theta \right), \end{array}$$

where q(j) denotes the time interval that the event occurred for subject j.

## 4 Application on Data From the PAX Program

### 4.1 Data Source and Study Population

The PAX Good Behavior Game (PAX) program was designed to improve children's mental, emotional, behavioral, and academic outcomes (https://www.paxis.org/school‐based‐programming/). In Manitoba, the Healthy Child Manitoba Office (HCMO), which was later incorporated into the Manitoba Education, initiated a cluster randomized control trial (CRCT) to evaluate the PAX program, implementing with Grade 1 students during the 2011–2012 school year [6]. A total of 196 schools participated and were randomly assigned to either the intervention group or the control group. Grade‐1 teachers in the intervention group attended a two‐day PAX training sessions and implemented the program in their classrooms [6]. Teachers in the control group continued with their standard classroom practices. The final PAX CRCT study cohort consisted of 4676 Grade‐1 students: 2576 in PAX group and 2100 in the control group [6].

The Strengths and Difficulties Questionnaire (SDQ), which is a widely used tool to assess children's mental health [40], was used in the PAX study to measure students' mental health. The total SDQ difficulty score is calculated as the sum of 20 items, each rated on a 3‐point Likert scale, with the value of 0, 1 and 2 indicating the categories of not true, somewhat true and certainly true. Higher scores indicate greater difficulties. In the PAX study, the SDQ was administered at two time points: before the PAX implementation (pre‐PAX, before January 2012) and after its completion (post‐PAX, June 2012). In May/June 2016, the HCMO conducted the Grade 5 (G‐5) Mental Health Survey, the province‐wide assessment of mental health among Grade 5 students in Manitoba, involving over 12 000 participants [8]. Of the original PAX study cohort, 3302 students (71%) also participated in the G‐5 survey, allowing for data linkage. Since the SDQ was also used in the G‐5 survey, the Grade 5 SDQ total score served as the third timepoint for measuring students' mental health in this study.

The data of the PAX study were collected and owned by HCMO and later transferred to the Manitoba Centre for Health Policy (MCHP). The MCHP (https://umanitoba.ca/manitoba‐centre‐for‐health‐policy/) is a Manitoba Population Research Data Repository, where the data are de‐identified and linkable to administrative databases via a scrambled identifier. By linking the PAX data to administrative records from Manitoba Health, Seniors and Active Living (MHSAL), Manitoba Families, Manitoba Education, we can access comprehensive information on social demographic, hospital and emergency room visits, medicine prescriptions, and involvement in special government programs [6].

This study has been approved by the Health Research Ethics Board (HREB) of the University of Manitoba (H2023:333). Data access was provided by MCHP for use of data contained in the Manitoba Population Research Data Repository under project #2024–043. Data access approval was provided by the Provincial Health Research Privacy Committee (PHRPC No. P2023‐110), Manitoba Education, and Manitoba Families.

### 4.2 Outcomes

The SDQ total score measured at the three time points: pre‐PAX (time 0), post‐PAX (time 0.5), and Grade 5 (time 4.5) served as the longitudinal outcome in this study. The timing reflects the actual intervals between assessments. Students from the PAX study were linked to the Hospital Abstract, Drug Product Information Network, and Medical Claims/Medical Services databases to obtain mental disorder diagnostics. This study focused on three mental disorders: attention‐deficit hyperactivity disorder (ADHD), conduct disorder, and mood and anxiety disorders.

A child mental disorder diagnosis was determined through hospitalizations or physician visits coded with specific ICD‐9‐CM or ICD‐10‐CA codes, as well as prescription records for specific drugs. For ADHD, a diagnosis was made if a child has at least one hospital or physician visit coded for ADHD, or at least two prescriptions for ADHD medications, or one prescription for ADHD drugs in one fiscal year and a diagnosis of hyperkinetic syndromes in the previous 3 years. Conduct disorder was diagnosed if there was at least one hospital or physician visit with a conduct disorder code. Mood and anxiety disorders were diagnosed if a child has one or more hospitalizations or physician visits coded for mood or anxiety conditions, plus at least two prescriptions for antidepressants or mood stabilizers depending on the ICD codes. Diagnoses typically apply to residents aged 3 years or older. More details can be found in the Appendix Table 7 in the PAX report prepared by the MCHP at the request from the Government of Manitoba [6]; this study used the same case definitions. The time‐to‐the first diagnosis of any of the three mental disorders, up to March 31, 2024, was used as the survival outcome in this study. Therefore, the study period spans from January 2012 (time zero) to March 31, 2024 (time 12.25), covering from Grade 1 to about 9 months after Grade 12 graduation.

### 4.3 Covariates

The covariates considered in this study were selected based on the PAX report and related publications [6, 7, 8]. At the student‐level, covariates were sex, socio‐economic factor index (SEFI), their mother's maternal mental disorder diagnosis before PAX (up to December 31, 2011), ever being a recipient of Child in Care (CIC) services (up to March 31, 2015), ever being a recipient of Child and Family Services (up to March 31, 2015), and ever being a recipient of family income assistance (IA) (up to March 31, 2015). The SEFI is an area‐level socioeconomic status (SES) indicator created by MCHP, where higher values indicate lower neighborhood SES compared to the provincial average [41]. At the classroom‐level, covariates included a binary indicator for PAX participation and an indicator to indicate if a classroom was attending an urban school. The classroom was considered as the group‐level rather than school level because the PAX intervention was classroom‐based, and most schools only had one or two Grade‐1 classrooms [42].

### 4.4 Statistical Analyses

The proposed MJM with a current‐value association structure was implemented to evaluate (a) the effect of PAX on the longitudinal SDQ total score, (b) the direct effect of PAX on the survival outcome—time to mental disorder diagnosis, and (c) the indirect effect of PAX on the time to mental disorder diagnosis mediated through SDQ.

We fitted three MJMs with different specifications for the longitudinal trajectory and selected the final model as the one with the smallest Deviance Information Criteria (DIC). When fitting the proposed MJMs, the follow‐up time in the survival submodel was split into three pieces to mimic the longitudinal data collection schedule: [0, 0.5), [0.5, 4.5), [4.5, 12.5). For comparison, we also estimated the standard JM that ignores the multilevel structure to assess the impact of ignoring the group level. To keep formulas concise, covariates other than PAX are omitted below.

MJM 1: Linear longitudinal trajectory

$$\begin{array}{cc} y_{\mathrm{jl}}\left(t_{\mathrm{ijl}}\right) & =m_{\mathrm{jl}}\left(t\right)+\varepsilon_{\mathrm{jl}}\left(t\right)\\ m_{\mathrm{jl}}\left(t\right) & =\beta_{000}+u_{0\mathrm{jl}}+v_{00l}+\left(\beta_{100}+u_{1\mathrm{jl}}\right)*t_{\mathrm{ijl}}\\ & \;+\beta_{001}*{\mathrm{PAX}}_{l}+\beta_{101}*\mathrm{PA}X_{l}*t_{\mathrm{ijl}}\\ \left[\begin{array}{c} u_{0\mathrm{jl}}\\ u_{1\mathrm{jl}} \end{array}\right] & ∼N\left(0,\sum_{u}\right),v_{00l}∼N\left(0,\sigma_{\nu }^{2}\right),\varepsilon_{\mathrm{jl}}\left(t\right)∼N\left(0,\sigma_{\varepsilon }^{2}\right)\\ \log\left(\mu_{\mathrm{jlq}}\right) & =\log t_{\mathrm{jlq}}+\log\lambda_{q}+\gamma *\mathrm{PA}X_{l}\left(t\right)\\ & \;+\alpha *m_{\mathrm{jl}}\left(t_{\mathrm{jlq}}\right)+g_{l},q=1,\ldots ,Q\\ g_{l} & ∼N\left(0,\sigma_{g}^{2}\right) \end{array}$$

MJM 2: Quadratic longitudinal trajectory, slope of time varies by PAX.

$$\begin{array}{cc} y_{\mathrm{jl}}\left(t_{\mathrm{ijl}}\right) & =m_{\mathrm{jl}}\left(t\right)+\varepsilon_{\mathrm{jl}}\left(t\right)\\ m_{\mathrm{jl}}\left(t\right) & =\beta_{000}+u_{0\mathrm{jl}}+v_{00l}+\beta_{100}*t_{\mathrm{ijl}}+\beta_{200}*t_{\mathrm{ijl}}^{2}\\ & \;+\beta_{001}*{\mathrm{PAX}}_{l}+\beta_{101}*\mathrm{PA}X_{l}*t_{\mathrm{ijl}}\\ u_{0\mathrm{jl}} & ∼N\left(0,\sigma_{u}\right),v_{00l}∼N\left(0,\sigma_{\nu }^{2}\right),\varepsilon_{\mathrm{jl}}\left(t\right)∼N\left(0,\sigma_{\varepsilon }^{2}\right)\\ \log\left(\mu_{\mathrm{jlq}}\right) & =\log t_{\mathrm{jlq}}+\log\lambda_{q}+\gamma *\mathrm{PA}X_{l}\left(t\right)+\alpha *m_{\mathrm{jl}}\left(t_{\mathrm{jlq}}\right)\\ & \;+g_{l},q=1,\ldots ,Q\\ g_{l} & ∼N\left(0,\sigma_{g}^{2}\right) \end{array}$$

MJM 3: Quadratic longitudinal trajectory, slope of time and slope of $time^{2}$ vary by PAX

$$\begin{array}{cc} y_{\mathrm{jl}}\left(t_{\mathrm{ijl}}\right) & =m_{\mathrm{jl}}\left(t\right)+\varepsilon_{\mathrm{jl}}\left(t\right)\\ m_{\mathrm{jl}}\left(t\right) & =\beta_{000}+u_{0\mathrm{jl}}+v_{00l}+\beta_{100}*t_{\mathrm{ijl}}+\beta_{200}*t_{\mathrm{ijl}}^{2}\\ & \;+\beta_{001}*{\mathrm{PAX}}_{l}+\beta_{101}*\mathrm{PA}X_{l}*t_{\mathrm{ijl}}+\beta_{201}*\mathrm{PA}X_{l}*t_{\mathrm{ijl}}^{2}\\ u_{0\mathrm{jl}} & ∼N\left(0,\sigma_{u}\right),v_{00l}∼N\left(0,\sigma_{\nu }^{2}\right),\varepsilon_{\mathrm{jl}}\left(t\right)∼N\left(0,\sigma_{\varepsilon }^{2}\right)\\ \log\left(\mu_{\mathrm{jlq}}\right) & =\log t_{\mathrm{jlq}}+\log\lambda_{q}+\gamma *\mathrm{PA}X_{l}\left(t\right)+\alpha *m_{\mathrm{jl}}\left(t_{\mathrm{jlq}}\right)\\ & \;+g_{l},q=1,\ldots ,Q\\ g_{l} & ∼N\left(0,\sigma_{g}^{2}\right) \end{array}$$

The longitudinal SDQ measurements were collected through Grade 5 (4.5 years) while the time‐to‐first mental disorder diagnosis was observed from January 2012 to March 2024. Consequently, no SDQ data exist for the final 7.75 years of follow‐up. Our joint model treats the SDQ trajectory up to 4.5 years as informative about subsequent risk through the current‐value association m(t) in the survival submodel. This relies on the substantive assumption that the SDQ trajectory captured up to 4.5 years provides information about the underlying risk trajectory beyond that point (i.e., that post‐4.5 SDQ trajectories can be meaningfully summarized by the individual's latent growth captured by the three‐level LMM and its random effects). In practice, this means we assume a continuation with a stable/linearly evolving slope estimated from earlier measurements, rather than a completely new, unobserved pattern after 2016. The hazard then depends on the inferred current SDQ value m(t) at time t, which is obtained from the longitudinal model via the random effects. This approach enables joint inference about how early‐life SDQ trajectories relate to longer‐term risk, while recognizing that post‐4.5 SDQ values are unobserved and the association is conditional on the post‐4.5 extrapolation implied by the longitudinal model.

All analyses were conducted on the MCHP server, a Windows 10 (64‐bit) virtual machine configured with an AMD EPYC 7H12 64‐Core Processor (2 cores, 2.60 GHz), 16 GB of RMA, and 500 MB of storage.

### 4.5 Results

After including students with at least one recorded SDQ total score and who did not have a clinical diagnosis of any of the three mental disorders, the final study cohort consisted of 7877 records from 3907 students. Of these, 1720 students were in 141 control group classrooms, and 2187 students were in 172 classrooms implementing the PAX program. The mean number of children per classroom was 12 (range: 1–25) in the control group and 13 (range: 1–28) in the PAX group.

Descriptive statistics are displayed in Table 1. The control and PAX groups had an almost even distribution of males and females. Compared to the control group, the students in the PAX group were more likely to come from families receiving income assistance, to have received services from CIC and CFS, to come from lower SES households, to attend urban schools, and to have a mother with maternal mental disorder diagnoses before January 2012.

**TABLE 1:** Descriptive statistics of the final study cohort.

	Control *N* = 1720	Pax *N* = 2187	*p*
Sex			0.4
Female	866 (50%)	1128 (52%)	
Male	854 (50%)	1059 (48%)	
Ever on IA	394 (23%)	590 (27%)	0.004
Ever in CIC	164 (10%)	256 (12%)	0.030
Ever in CFS	354 (21%)	556 (25%)	< 0.001
Student SEFI			0.002
*N* non‐missing	1720	2187	
Mean (SD)	0.17 (0.99)	0.28 (1.21)	
Min–Max	−2.4 to 3.81	−3.01 to 3.81	
School urbanicity	792 (46%)	908 (42%)	0.005
Mother's maternal mental disorder prior to PAX	590 (34%)	826 (38%)	0.025

*Note:* Ever on IA/CIC/CFS indicates if the child/family has ever received IA/CIC/CFS before 31 March 2015.

Abbreviations: CFS—child family services; CIC—child in care; IA – income assistance; SD – standard deviation; SEFI—area‐level socioeconomic status.

**FIGURE 1:**
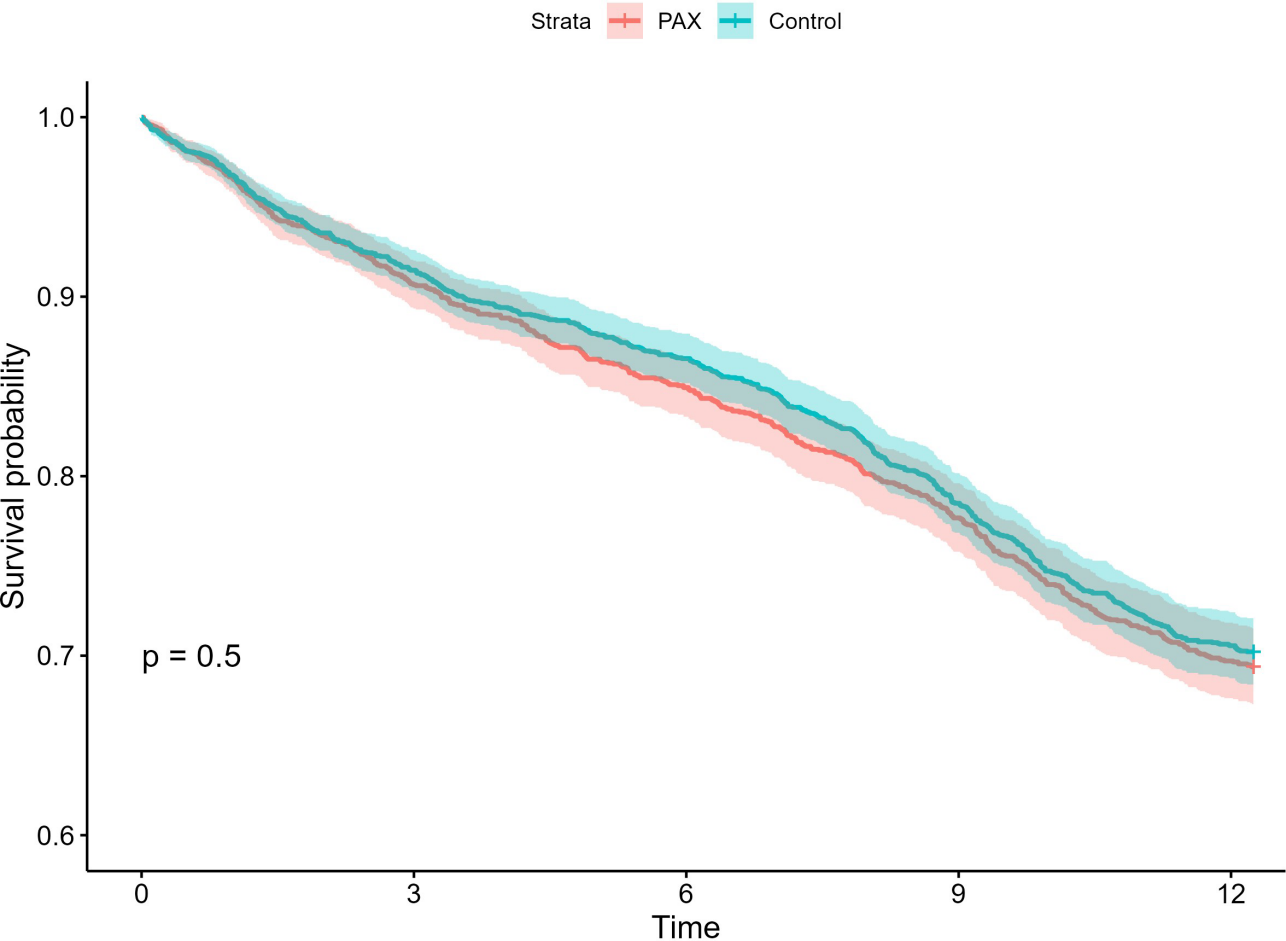
Kaplan–Meier survival curves for the control and PAX group.

Table 2 shows the summary statistics for the survival outcome in this study. By March 31, 2024, 1153 (29.5%) students had received at least one diagnosis of any of the three mental disorders resulting in a right censoring rate of 70.5%. Of these, 561 (30.0%) students were in the control group and 637 (29.1%) were in the PAX group. The median follow‐up times until diagnosis were 6.16 years (around Grade 6) for the control group and 7.04 years (around Grade 7) for the PAX group. Figure 1 displays the survival curves for the control group and the PAX group. The log‐rank test results (*p* value in Figure 1) indicated that the survival curves were not significantly different between the two groups.

**TABLE 2:** Descriptive statistics for the outcomes.

	Control *N* = 1720	Pax *N* = 2187	*p*
Mental disorder diagnosis	516 (30.0%)	637 (29.1%)	0.6
Total difficulty score			
Pre‐PAX			< 0.001
*N* non‐missing (%)	1242 (72.2%)	1815 (82.9%)	
Mean (SD)	7.46 (6.76)	9.06 (7.46)	
Min–Max	0–34	0–40	
Post‐PAX			0.6
*N* non‐missing (%)	964 (56.1%)	1196 (54.7%)	
Mean (SD)	6.98 (6.76)	6.71 (6.60)	
Min–Max	0–33	0–35	
Grade 5			0.6
*N* non‐missing (%)	1283 (74.6%)	1377 (63.0%)	
Mean (SD)	7.87 (7.36)	7.66 (7.03)	
Min– Max	0–35	0–34.25	

**FIGURE 2:**
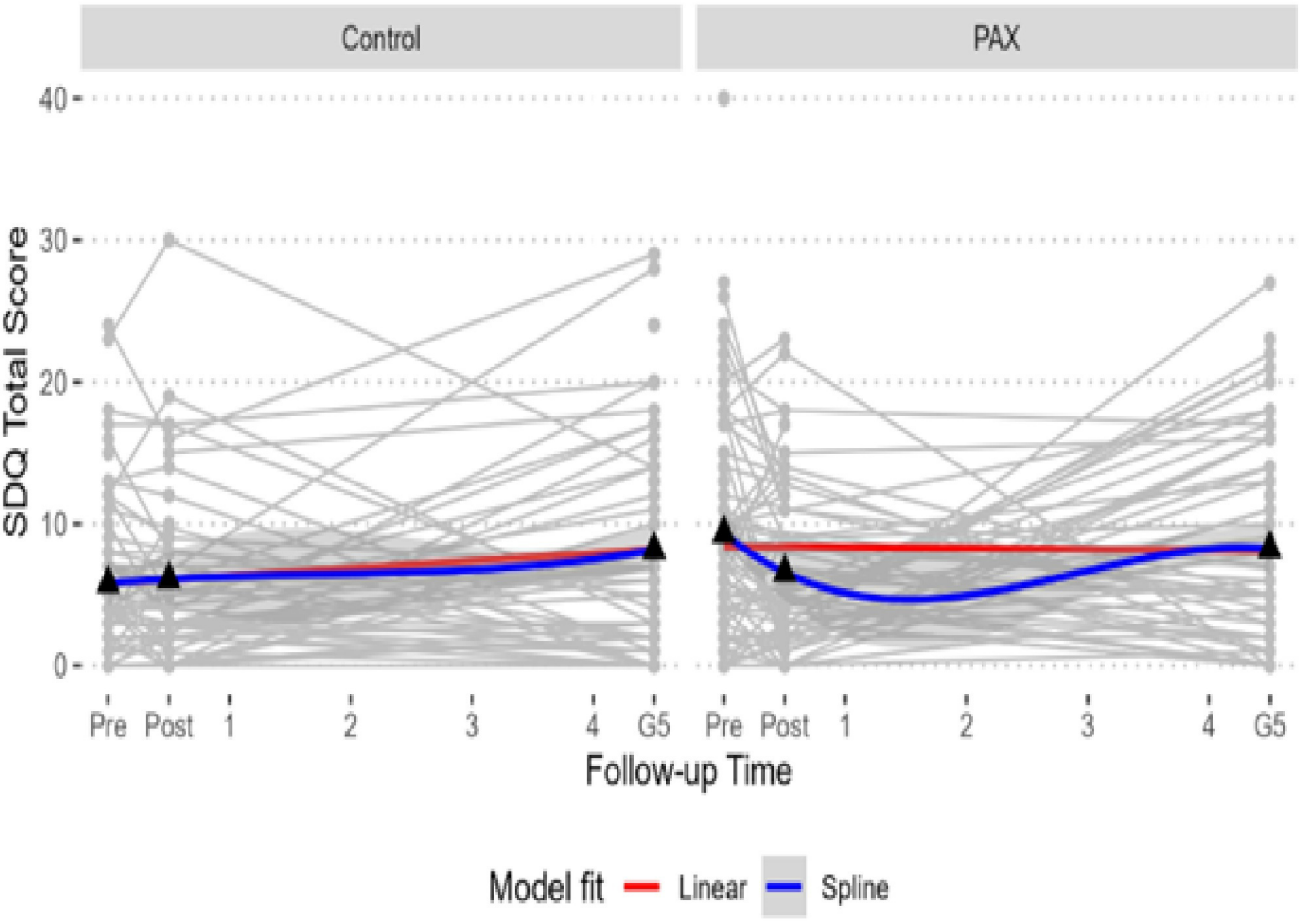
Longitudinal trajectories of SDQ total score for a random 200 subjects in both control and PAX group.

Table 2 displays the summary statistics for the SDQ total score measured at three time points. At the baseline (pre‐PAX), the mean SDQ total difficulty score was higher in the PAX group than in controls; by post‐PAX and Grade 5, the means were similar between groups. Figure 2 shows the observed SDQ total score trajectories for a random sample of 200 participants in each group. Visual inspection suggests an approximately linear trajectory for the control group, whereas the PAX group appears non‐linear (quadratic or cubic). Given only three longitudinal measurements, we considered two plausible patterns: (1) a linear trajectory with random intercepts and slopes at the individual level (MLM 1) and (2) a quadratic trajectory with only random intercepts at the individual level (MLM 2 and MJM 3). Prior literature indicates considerable heterogeneity in SDQ trajectories across populations, with linear, quadratic, or piecewise linear/quadratic patterns often observed from early childhood (around age 5) to late adolescence (around age 17) [43, 44]. Therefore, the three measurements from Grade 1 to Grade 5 may be informative for distinguishing between approximately linear and quadratic trajectories.

As described in Section 5 the simulation studies, posterior samples were obtained from three parallel MCMC chains, with adaption phase of 100 iterations followed by 34 000 sampling iterations. For the MJM 1 assuming a linear trajectory, the first 4000 iterations were discarded as burn‐in based on the traceplots of the raw MCMC chains (see Supporting Information: Appendix 3). To address autocorrelation (see Supporting Information: Appendix 4) and due to limited storage on the MCHP server, a thinning interval of 150 were applied, resulting in 200 posterior samples retained per chain. Therefore, a total of 600 samples were used for inference. After thinning, the autocorrelation among the posterior samples was substantially reduced (see Supporting Information: Appendix 5). To ensure that this thinning did not compromise the reliability of the inferences, we conducted post hoc diagnostic checks. We report Gelman–Rubin‐Brooks (R‐hat) and ACF plots for all primary parameters, including the association parameter α and the variance components, and we provide trace plots for the three chains. Convergence of the thinned MCMC chains for all parameters across the three MJMs was confirmed based on visual inspection of the traceplots (see Supporting Information: Appendix 6) and the $\hat{R}$ statistics (see Supporting Information: Appendix 7).

Among the three MJMs, MJM 1—which assumed a linear trajectory of SDQ with random intercepts and slopes at the individual level—achieved the smallest DIC (see Table 3) and was therefore chosen as the final model. A sensitivity analysis for thinning interval was conducted for the final model, showing that parameter estimates and standard deviations before and after thinning are very close (see Supporting Information: Appendix 8).

**TABLE 3:** DIC of the three MJMs for the PAX study.

Model	DIC
MJM1	54655.95
MJM2	60043.95
MJM3	60043.70

Table 4 displays the results of the selected MJM with and without covariates, using the PAX data. Without adjusting for covariates, the average total difficulties in the control group did not significantly change over time (β = 0.106, 95% CI: −0.021 to 0.234). In contrast, the PAX program significantly reduced the total difficulties by 0.306 units (95% CI: 0.138–0.479) per year. This PAX effect remained statistically significant after adjusting for covariates. The PAX group had significantly 1.002 units (95% CI: 0.534–1.466) higher mean total difficulties in the baseline (i.e., Grade 1), however, by Grade 5, mean total difficulties were similar between the two groups.

**TABLE 4:** Results of proposed multilevel joint model with and without covariates.

Parameters			MJM without covariates				MJM with covariates			
			Estimate	SD	Q2.5	Q97.5	Estimate	SD	Q2.5	Q97.5
Survival	Fixed effects	PAX	−0.048	0.062	−0.170	0.079	−0.064	0.061	−0.182	0.046
		Sex M					−0.461	0.061	−0.586	−0.335
		Student SEFI					−0.206	0.032	−0.269	−0.187
		Urban School					0.264	0.061	0.456	0.387
		Ever on IA					0.191	0.079	0.044	0.334
		Ever in CIC					0.279	0.103	0.076	0.476
		Ever in CFS					0.187	0.093	−0.010	0.360
		Maternal mental disorder					0.569	0.062	0.445	0.688
		Association	0.055*	0.006	0.043	0.065	0.056*	0.006	0.045	0.066
	Random effects	Intercept at group level (SD)	0.124	0.041	0.059	0.218	0.142	0.047	0.059	0.217
Longitudinal	Fixed effect	Intercept	7.334	0.193	6.961	7.723	5.135	0.236	4.655	5.566
		Time	0.106	0.064	−0.021	0.234	0.552	0.078	0.383	0.705
		PAX	1.002*	0.244	0.534	1.466	0.769*	0.247	0.282	1.264
		Sex M					2.301	0.229	1.830	2.712
		Student SEFI					0.522	0.092	0.336	0.702
		Urban School					−0.578	0.183	−0.927	−0.222
		Ever on IA					2.695	0.321	2.090	3.294
		Ever in CIC					1.399	0.484	0.401	2.303
		Ever in CFS					1.570	0.370	0.795	2.277
		Maternal mental disorder					0.561	0.177	0.230	0.899
		Time*PAX	−0.306*	0.083	−0.479	−0.138	−0.249*	0.080	−0.401	−0.096
		Time*sex M					−0.387	0.080	−0.512	−0.234
		Time*IA					−0.626	0.110	−0.845	−0.404
		Time*CIC					−0.439	0.166	−0.747	−0.109
		Time*CFS					−0.317	0.135	−0.585	−0.072
	Random effects	Intercept at individual level (variance)	42.405	1.297	40.002	44.926	36.045	1.188	33.988	38.694
		Time (variance)	4.140	0.159	3.824	4.457	3.852	0.149	3.580	4.167
		Intercept*time (covariance)	−9.642	0.398	−10.42	−8.847	−8.310	0.367	−9.112	−7.675
		Residual (SD)	3.353	0.056	3.242	3.454	3.356	0.055	3.253	3.464
		Intercept at group level (SD)	0.244	0.132	0.070	0.544	0.241	0.134	0.074	0.561

*Note:* * indicates the significant estimates.

Table 4 also shows that higher total difficulty scores were significantly associated with an increased risk of mental disorder diagnoses (hazard ratio (HR) = $e^{0.055}=1.057$, 95% CI: 1.0474–1.067) and this association remained significant after covariates adjustment. Therefore, the PAX program indirectly reduced the risk of clinical mental disorder diagnoses through decreasing the total difficulties over time. Additionally, the PAX program showed trend toward directly reducing the risk of mental disorder diagnoses (HR = $e^{-0.048}=$ 0.953, 95% CI: 0.844–1.050), although this direct effect was not statistically significant.

Regarding the impact of covariates, several notable patterns emerged. Male students had significantly higher mean total difficulty scores at baseline (Grade 1) by 2.301 units (95% CI: 1.830–2.712) compared to females. However, male students' annual rate of increase was significantly slower than that of female students by 0.387 units (95% CI: 0.234–0.512) per year. Additionally, male students had a 36.9% lower risk of being diagnosed with a mental disorder compared to females (95% CI: 28.5%–55.7%). It is important to interpret this direct effect of sex with caution as there is a positive association between total difficulty scores and the risk of mental health disorders.

Additional patterns were observed for other covariates:

- Students from lower SES neighbourhood had significantly higher mean total difficulty scores in Grade 1, but significantly lower risk of having a mental health disorder.
- Students attending urban schools had significantly lower mean total difficulty score in Grade 1 but significantly higher risk of having a mental disorder diagnosis.
- Students whose mother had a maternal mental disorder diagnosis had significantly higher baseline total difficulty scores and significantly higher hazard of having a mental health disorder.
- Students from families receiving income assistance had significantly higher mean total difficulty scores in Grade 1 and significantly slower yearly changing rate over time compared to students from families not receiving income assistance. These students also had a higher risk of being diagnosed with a mental health disorder.
- Students from families that were recipients of services from CIC had significantly higher baseline total difficulty scores and significantly slower yearly changing rates over time, compared to children from families that did not receive CIC services.
- Students from families that were recipients of services from CFS had significantly higher baseline total difficulty scores and significantly slower yearly changing rates over time, compared to children from families that did not receive services from CFS. These students also had a higher risk of being diagnosed with a mental health disorder.

The random effects for both intercept and the slope of time at the individual level, as well as the random effects at the group level, were significant in both the longitudinal and survival submodels. This indicated that students from the same classroom had significantly different baseline total difficulty scores and rates of change over time. Additionally, classrooms differed significantly in their average baseline total difficulty scores and baseline hazards of developing a mental health disorder. Importantly, these variations between classrooms in both the average total difficulty score and the baseline hazard could only be identified through the use of multilevel joint models. Traditional joint models would not account for these group‐level differences, highlighting the value of the multilevel joint modeling approach in uncovering classroom‐level effects that may otherwise remain hidden.

Table 5 shows a comparison between the selected MJM and standard JM, the latter of which does not account for the group‐level effects. When the group‐level was ignored, the estimated effect of PAX in decreasing total difficulties was underestimated by 21% (= (0.249–0.196)/0.249), and its direct effect in decreasing the risk of mental disorder diagnosis was underestimated by 18% (= (0.064–0.053)/0.064). The indirect effect of PAX in decreasing the risk of mental disorder through decreasing the total difficulty score was also underestimated. Specifically, the association between total difficulties and the hazard was underestimated by 45% (= (0.056–0.031)/0.056), and the relationship between PAX and the total difficulties was underestimated by 4%. Ignoring the group‐level further led to substantial underestimation (over 60%) of the variation in both baseline difficulties and the changing rate of total difficulties over time among students. Moreover, failing to account for the group‐level prevented the analysis from capturing the variation in both baseline total difficulties and baseline hazard of mental disorder diagnosis across classrooms.

**TABLE 5:** Results of MJM versus standard JM ignoring the group level.

Parameters			MJM				JM				Percent of difference
			Estimate	SD	Q2.5	Q97.5	Estimate	SE	*Z*‐value	*p*	Estimates
Survival	Fixed effects	PAX	−0.064	0.061	−0.182	0.046	−0.053	0.060	−0.869	0.385	−18%
		Sex M	−0.461	0.061	−0.586	−0.335	−0.380	0.061	−6.200	< 0.0001	−18%
		Student SEFI	−0.206	0.032	−0.269	−0.187	−0.195	0.032	−6.179	< 0.0001	−6%
		Urban School	0.264	0.061	0.456	0.387	0.248	0.061	4.070	< 0.0001	−6%
		Ever on IA	0.191	0.079	0.044	0.334	0.262	0.081	3.235	0.001	37%
		Ever in CIC	0.279	0.103	0.076	0.476	0.332	0.107	3.108	0.002	19%
		Ever in CFS	0.187	0.093	−0.010	0.360	0.225	0.093	2.417	0.016	20%
		Maternal mental disorder	0.569	0.062	0.445	0.688	0.577	0.062	9.301	< 0.0001	1%
		Association	0.056*	0.006	0.045	0.066	0.031	0.007	4.317	< 0.0001	−45%
	Random effects	Frailty (SD)	0.142	0.047	0.059	0.217	—				
Longitudinal	Fixed effect	Intercept	5.135	0.236	4.655	5.566	4.958	0.218	22.760	< 0.0001	−3%
		Time	0.552	0.078	0.383	0.705	0.560	0.073	7.635	< 0.0001	1%
		PAX	0.769*	0.247	0.282	1.264	0.737	0.210*	3.514	0.000	−4%
		Sex M	2.301	0.229	1.830	2.712	2.407	0.210	11.453	< 0.0001	5%
		Student SEFI	0.522	0.092	0.336	0.702	0.545	0.085	6.395	< 0.0001	4%
		Urban School	−0.578	0.183	−0.927	−0.222	−0.666	0.163	−4.082	< 0.0001	15%
		Ever on IA	2.695	0.321	2.090	3.294	2.304	0.283	8.143	< 0.0001	−15%
		Ever in CIC	1.399	0.484	0.401	2.303	1.382	0.427	3.236	0.001	−1%
		Ever in CFS	1.570	0.370	0.795	2.277	1.876	0.330	5.693	< 0.0001	20%
		Maternal mental disorder	0.561	0.177	0.230	0.899	0.641	0.172	3.718	0.000	14%
		Time*PAX	−0.249*	0.080	−0.401	−0.096	−0.196	0.078*	−2.517	0.012	−21%
		Time*sex M	−0.387	0.080	−0.512	−0.234	−0.424	0.081	−5.251	< 0.0001	10%
		Time*IA	−0.626	0.110	−0.845	−0.404	−0.570	0.106	−5.384	< 0.0001	−9%
		Time*CIC	−0.439	0.166	−0.747	−0.109	−0.451	0.162	−2.788	0.005	3%
		Time*CFS	−0.317	0.135	−0.585	−0.072	−0.352	0.125	−2.808	0.005	11%
	Random effects	Intercept at individual level (variance)	36.045	1.188	33.988	38.694	14.231				−61%
		Time (variance)	3.852	0.149	3.580	4.167	1.393				−64%
		Intercept*time (covariance)	−8.310	0.367	−9.112	−7.675	−3.353				−60%
		Residual (SD)	3.356	0.055	3.253	3.464	6.400				91%
		Intercept at group level (SD)	0.241	0.134	0.074	0.561	—				

Additionally, disregarding the group‐level structure also influenced the estimation of covariate effects in both the longitudinal and survival submodels. Not accounting for group‐level clustering tends to bias the regression coefficients for covariates, as the variance associated with group differences is erroneously attributed to individual‐level variation. This can lead to either underestimation or overestimation of covariate effects, as shown in Table 4, ultimately compromising the accuracy and interpretation of the model findings. Thus, consideration of the group‐level is essential for obtaining unbiased and reliable estimates of both intervention effects and covariate associations in joint modeling.

## 5 Simulation Study

Comprehensive simulations were conducted to assess the performance of the proposed joint model for multilevel longitudinal and survival data. In addition, the results of the proposed MJM were compared to the results of traditional JM, which the group level was ignored.

### 5.1 Data Generation

First, random effects at the subject and group level were generated for the two processes. Then, event time in the survival process was generated. The data in the longitudinal process were generated based on the event times in the survival process, with repeated measurements recorded until the last measurement time before the event occurs. The participants were assumed to be followed for 5 years for the repeated measurements and 9 years of follow‐up for the survival outcome.

The Weibull distribution was used to model the baseline hazard function, with the shape and scale parameters determined based on the censoring rate in the simulation design and the Kaplan–Meier curve in the real data. The ***simsurv*** package, which allows time‐varying covariates, was used to simulate event times in the survival process [45]. This package was built according to the methodology developed by Crowther and Lambert [46] which used Gauss‐Kronrod quadrature to calculate the cumulative hazard function and numerical root finding to determine the time. The user‐specific hazard function with time‐varying covariates was determined based on the association structure between the longitudinal and survival processes, as well as the covariates in both submodels. Individuals whose event time exceeded the follow up period were considered as right censored.

In the longitudinal process, the time intervals between two consecutive measurements were assumed to be equal and measured every 6 months. The total follow‐up time is assumed to be 5 years, resulting in that a total of 10 repeated measurements and one baseline measurement were generated. The data were generated using the following model (Equation 5), assuming random intercepts and slopes of time at subject‐level and random intercepts at group‐level in both the longitudinal and survival submodel. The intervention was assumed to be implemented at the group level.

(5) $$\begin{array}{cc} y_{\mathrm{jl}}\left(t_{\mathrm{ijl}}\right) & =m_{\mathrm{jl}}\left(t\right)+\varepsilon_{\mathrm{jl}}\left(t\right)\\ m_{\mathrm{jl}}\left(t\right) & =\beta_{000}+u_{0\mathrm{jl}}+v_{00l}+\left(\beta_{100}+u_{1\mathrm{jl}}\right)*t_{\mathrm{ijl}}\\ & \;+\beta_{001}*{\mathrm{TRT}}_{\mathrm{jl}}+\beta_{101}*\mathrm{TR}T_{\mathrm{jl}}*t_{\mathrm{jli}}\\ \left[\begin{array}{c} u_{0\mathrm{jl}}\\ u_{1\mathrm{jl}} \end{array}\right] & ∼N\left(0,\sum_{u}\right),v_{00l}∼N\left(0,\sigma_{\nu }^{2}\right),\varepsilon_{\mathrm{jl}}\left(t\right)∼N\left(0,\sigma_{\varepsilon }^{2}\right)\\ \lambda_{\mathrm{jl}}\left(t\right) & =\lambda_{0}\left(t\right)\exp\left\{\alpha *m_{\mathrm{jl}}\left(t\right)+g_{l}\right\}\\ g_{l} & ∼N\left(0,\sigma_{g}^{2}\right)\\ \lambda_{0}\left(t\right) & =\text{scale}*\text{shape}*t^{\text{shape}-1} \end{array}$$

where $y_{\mathrm{jl}}\left(t_{\mathrm{ijl}}\right)$ is the longitudinal outcome measured at time $t_{\mathrm{ijl}}$ for subject j from group l; $\beta_{000}$ indicates the mean of the longitudinal outcome at the baseline; $\beta_{100}$ indicates the average changing rate of the longitudinal outcome in the control group; $\beta_{001}$ indicates the difference of the baseline longitudinal outcome in the treatment group compared to the control group; $\beta_{101}$ indicates the difference of the changing rate in the treatment group compared to the control group, in other words, it indicates the treatment effect; $u_{0\mathrm{jl}}$ indicates the variation of the baseline longitudinal outcome among individuals from the same group; $v_{00l}$ indicates the variation of the average baseline measure among the groups; $u_{1\mathrm{jl}}$ indicates the variation of the changing rate of the longitudinal outcome among individuals from the same group; $\lambda_{0}\left(t\right)$ indicates the baseline hazard function follows a Weibull distribution with a scale and a shape parameter; $m_{\mathrm{jl}}\left(t\right)$ indicates the linear predictor of the longitudinal outcome; α indicates the association parameter between the longitudinal outcome and the survival outcome, also reflects the indirect effect of the intervention on the survival outcome; $g_{l}$ indicates the variation in the hazard among individuals from the same group. The subject‐level random effects $u_{\mathrm{jl}}$ follows a multivariate normal distribution with variance–covariance matrix $\sum_{u}$, the group‐level random effects $v_{l}$ and $g_{l}$ follow a normal distribution with the variance of $\sigma_{\nu }^{2}$ and $\sigma_{g}^{2}$. The level‐1 residual $\varepsilon_{\mathrm{jl}}\left(t\right)$ is assumed to follow a normal distribution $N\left(0,\sigma_{\varepsilon }^{2}\right)$.

### 5.2 Simulation Design

The true values used in the simulation study were determined based on the existing literature that utilized data from the PAX program (Table 6) [6, 7]. However, none of these studies considered joint models in their analysis. While Jiang [8] examined the effect of PAX intervention on the odds of being diagnosed with a mental disorder using multilevel logistic regression, time‐to‐event outcomes and joint modeling approaches were not addressed in previous research [8]. Therefore, the true value for the association between SDQ score and time‐to‐any mental disorder in the simulation study was specified based on the odds ratios reported in the literature.

**TABLE 6:** True values of parameters in the simulation study.

$\beta_{000}$	$\beta_{001}$	$\beta_{100}$	$\beta_{101}$	$\sigma_{u_{0\mathrm{jl}}}^{2}$	$\sigma_{u_{1\mathrm{jl}}}^{2}$	$\rho_{u_{0\mathrm{jl}}u_{1\mathrm{jl}}}$	$\sigma_{\varepsilon }^{2}$	α
16	1.6	−0.8	−2	10	10	0	33	−0.2

#### 5.2.1 Impact of Ignoring the Group Level

To investigate the impact of ignoring the group‐level structure on parameter estimates, we compared two models. The first model is a three‐level model that assumes random intercepts at the group‐level in both the longitudinal and survival submodels. The second model is a two‐level model that ignores the within‐group correlation among subjects. We conducted this comparison under 24 different conditions, varying by the two group‐level ICCs in the longitudinal submodel, two variances of the random effects ($\sigma_{g}^{2}$) in the survival submodel, three sample sizes at the group level (i.e., numbers of groups), and two censoring rates.

Model 1: Three‐level model with random intercepts and random slopes at subject level and random intercepts at the group level in the longitudinal submodel, random intercepts at the group level in the survival submodel.

$$y_{\mathrm{jl}}\left(t\right)=m_{\mathrm{jl}}\left(t\right)+\varepsilon_{\mathrm{jl}}\left(t\right)$$

$$\begin{array}{ll} m_{\mathrm{jl}}\left(t\right) & =\beta_{000}+u_{0\mathrm{jl}}+v_{00l}+\left(\beta_{100}+u_{1\mathrm{jl}}\right)*t_{\mathrm{ijl}}+\beta_{001}*{\mathrm{TRT}}_{\mathrm{jl}}\\ & \;+\beta_{101}*{\mathrm{TRT}}_{\mathrm{jl}}*t_{\mathrm{ijl}} \end{array}$$

$$\log\left(\mu_{\mathrm{jlq}}\right)=\log t_{\mathrm{jlq}}+\log\lambda_{q}+\alpha *m_{\mathrm{jlq}}\left(t\right)+g_{l},$$

where $y_{\mathrm{jl}}$ is the longitudinal outcome measured at time t for subject j from group l and the $m_{\mathrm{jl}}\left(t\right)$ is the true longitudinal outcome at time t; $\varepsilon_{\mathrm{jl}}\left(t\right)$ indicates the level‐1 residual or measurement error; $\beta_{000}$ indicates the mean of the longitudinal outcome at the baseline; $\beta_{100}$ indicates the average changing rate of the longitudinal outcome in the control group; $\beta_{001}$ indicates the difference of the baseline longitudinal outcome in the treatment group or intervention group compared to the control group; $\beta_{101}$ indicates the difference of the changing rate in the treatment (intervention) group compared to the control group, in other words, it indicates the treatment effect; $u_{0\mathrm{jl}}$ indicates the variation of the baseline longitudinal outcome among individuals from the same group; $v_{00l}$ indicates the variation of the average baseline measure among the groups; $u_{1\mathrm{jl}}$ indicates the variation of the changing rate of the longitudinal outcome among individuals from the same group; $\mu_{\mathrm{jlq}}$ indicates the auxiliary Poisson outcome for the time interval q for subject j from the group l; $t_{\mathrm{jlq}}$ indicates the exposure time in the time interval q for subject_jl_; $\lambda_{q}$ indicates the baseline hazard at time interval q; α indicates the association parameter between the longitudinal outcome and the survival outcome; $g_{l}$ indicates the variation in the hazard among individuals from the same group.

Model 2: Two‐level model with random intercepts and random slopes at subject level in the longitudinal submodel.

$$y_{j}\left(t\right)=m_{j}\left(t\right)+\varepsilon_{j}\left(t\right)$$

$$m_{j}\left(t\right)=\beta_{00}+u_{0j}+\beta_{01}*{\mathrm{TRT}}_{j}+\left(\beta_{10}+\beta_{11}*{\mathrm{TRT}}_{j}+u_{1j}\right)*t_{\mathrm{ij}}$$

$$\log\left(\mu_{\mathrm{jq}}\right)=\log t_{\mathrm{jq}}+\log\lambda_{q}+\alpha *m_{\mathrm{jq}}\left(t\right).$$

where $y_{j}$ is the longitudinal outcome measured at time t for subject j and the $m_{j}\left(t\right)$ is the true longitudinal outcome at time t; $\varepsilon_{j}\left(t\right)$ indicates the level‐1 residual or measurement error; $\beta_{00}$ indicates the mean of the longitudinal outcome at the baseline; $\beta_{10}$ indicates the average changing rate of the longitudinal outcome in the control group; $\beta_{01}$ indicates the difference of the baseline longitudinal outcome in the treatment (intervention) group compared to the control group; $\beta_{11}$ indicates the difference of the changing rate in the treatment (intervention) group compared to the control group, that is, the treatment effect; $u_{0j}$ indicates the variation of the baseline longitudinal outcome among the individuals; $u_{1j}$ indicates the variation of the changing rate of the longitudinal outcome among individuals; $\mu_{\mathrm{jq}}$ indicates the auxiliary Poisson outcome for the time interval q for subject j; $t_{\mathrm{jq}}$ indicates the exposure time in the time interval q for subject_j_; $\lambda_{q}$ indicates the baseline hazard at time interval q; α indicates the association parameter between the longitudinal outcome and the survival outcome.

#### 5.2.2 Simulation Conditions

Two censoring rates, 20% and 60%, were considered in the simulation. Both sample size and the intraclass correlation coefficient (ICC) are two important factors in multilevel analysis and were discussed in the study design. The 30/30 rule, which requires a minimum of 30 groups and 30 individuals per group, was suggested in two‐level research [47, 48]. When the study interest is more on the random effects, the lowest number of subjects recommended in the literature is 10, while a larger number of groups such as 50 or 100 is needed [49, 50, 51]. In a three‐level model, it requires at least 50 groups to obtain accurate fixed effects, and at least 100 groups to reveal statistically significant random effects [52]. Based on these recommendations, we considered three sample sizes at the group level (i.e., numbers of groups = 50, 100, 200), each with a fixed group size of 30 individuals per group. The 30 children per class is common in educational research.

The ICC quantifies the amount of variation that is explained in each level. There are three levels in the longitudinal submodel and two levels in the survival submodel. To focus on the group‐level random effects, we fixed the variance of level 1 residual and level 2 random effects in the longitudinal submodel based on the literature of the PAX program. According to previous research on behavioral school‐based program, ICC at the school‐level ranges from 0.05 to 0.25 [51]. Therefore, we considered two ICC values (0.1 and 0.3) at the group level for the longitudinal submodel. For the ICC calculation, we adopted the definition in Davis and Scott [53] for the longitudinal submodel. Based on the intercept only model, the ICC at subject‐level equals $\sigma_{u_{0\mathrm{jl}}}^{2}/\left(\sigma_{u_{0\mathrm{jl}}}^{2}+\sigma_{\varepsilon }^{2}+\sigma_{v}^{2}\right)$ while the ICC at group‐level equals $\sigma_{v}^{2}/\left(\sigma_{u_{0\mathrm{jl}}}^{2}+\sigma_{\varepsilon }^{2}+\sigma_{v}^{2}\right)$. Thus, the two values for $\sigma_{v}^{2}$ in the simulation study are 5 and 18. For time‐to‐event data using frailty model, no standard definition for ICC is available [54], not even to mention in the mixed‐effect Cox models. Moreover, available methods for frailty models are to estimate the ICC based on the event indicator, or observed times, or a combination of the two [55]. They are ad‐hoc estimations after the data are available, which is not helpful in choosing the value of the variances of random effects ($\sigma_{g}^{2}$) in survival submodel in the simulation study. Therefore, in the current study, $\sigma_{g}^{2}=0.4$ was selected based on the existing literature on PAX, although a mixed‐effect logistic regression was used in that study. We also considered a larger school‐level random effects in the survival submodel (i.e., $\sigma_{g}^{2}=1$) to investigate the effect of ignoring the group level in the estimates. Different number of time intervals using mixed‐effect Poisson approach (Q = 11,7,3) were compared.

A total of 72 (= 2*3*1*2*2*3) scenarios were considered, with each scenario involving 200 simulation replicates. To generate the Bayesian estimator and make Bayesian inference, 2000 independent posterior samples were drawn from the stable posterior MCMC chains. The number of burn‐in iterations, thinning intervals, and total iterations were determined according to inspection of the trace and ACF plots to ensure adequate mixing and convergence. To evaluate the performance of the proposed MJM, we reported the mean estimates, average bias, average relative bias, mean estimated standard error (MESE), and empirical standard error (ESE).

### 5.3 Simulation Results

**FIGURE 3:**
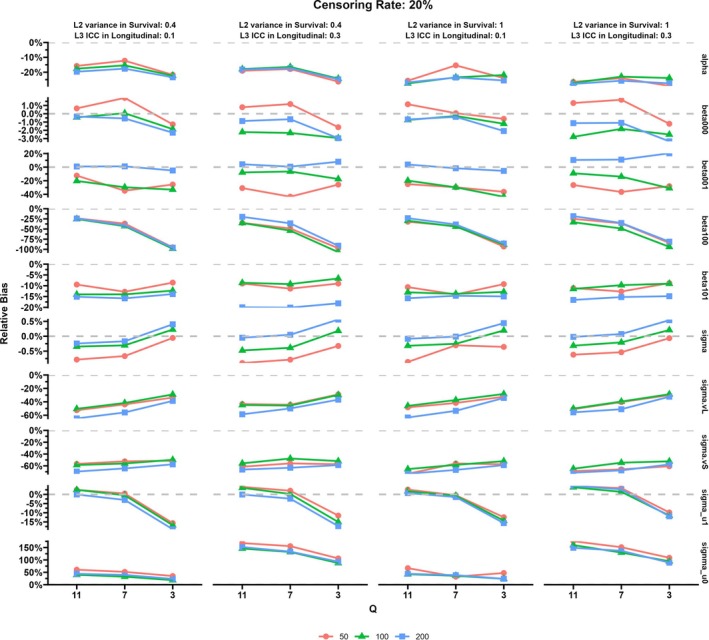
Simulation results: Relative bias of parameters in the multilevel joint model, censoring rate of 20%.

**FIGURE 4:**
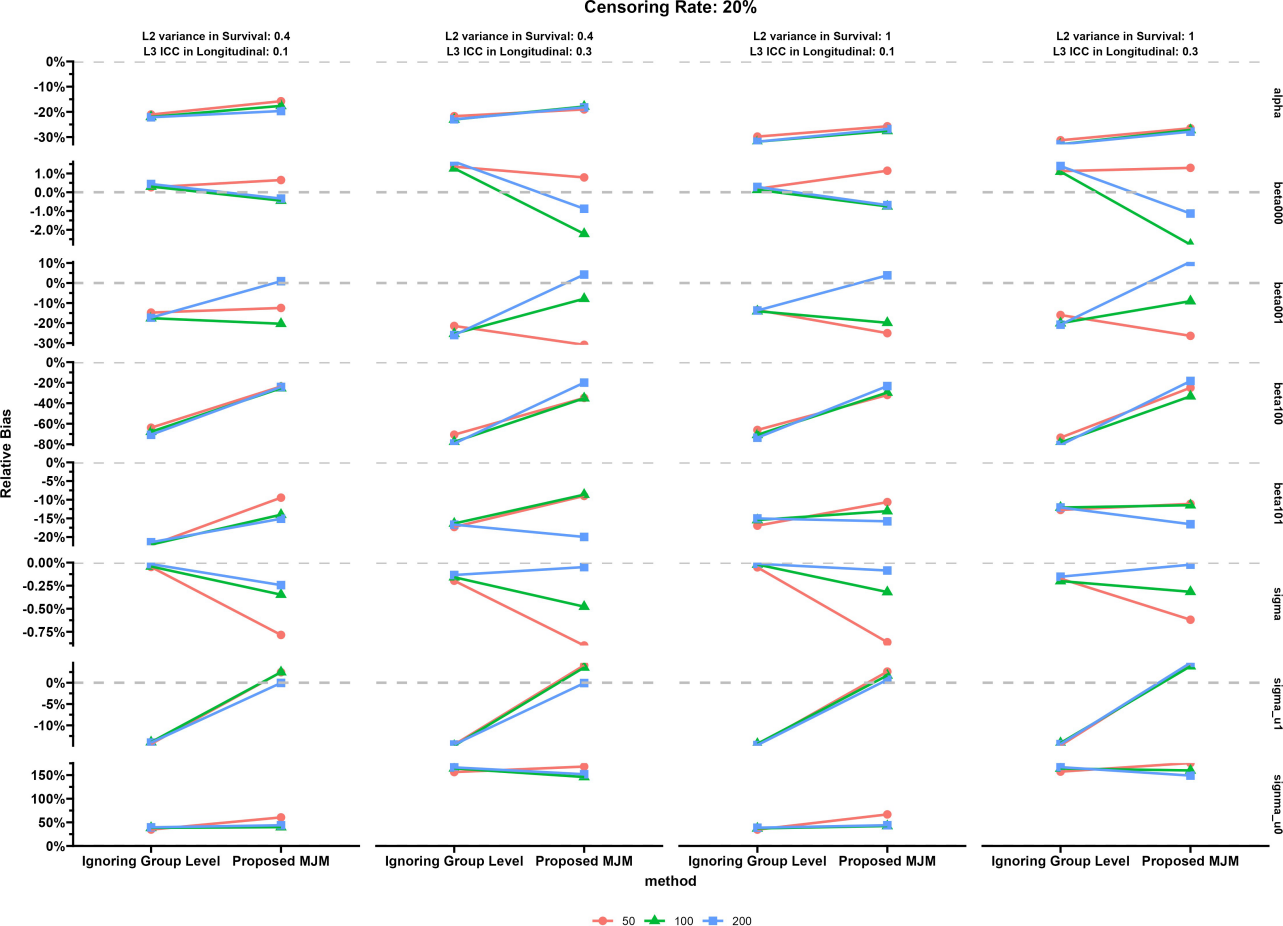
Simulation results: Relative bias of parameters in the multilevel joint model, censoring rate of 60%.

**FIGURE 5:**
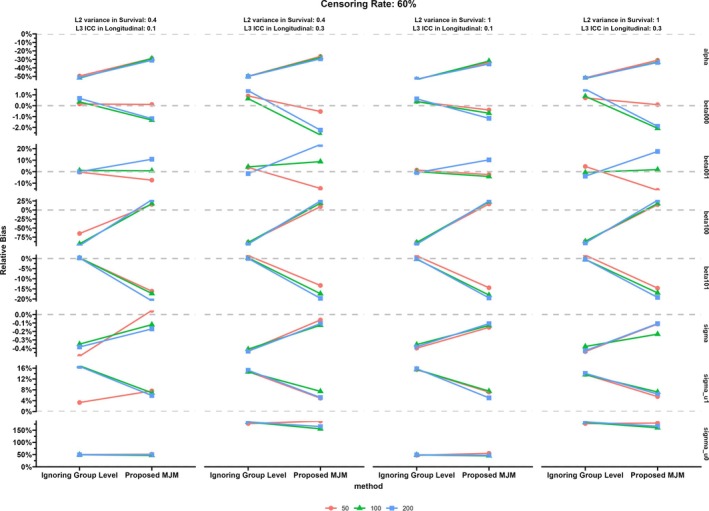
Simulation results: Comparison of results from MJM versus standard JM (ignored the group level), censoring rate of 20%.

**FIGURE 6:**
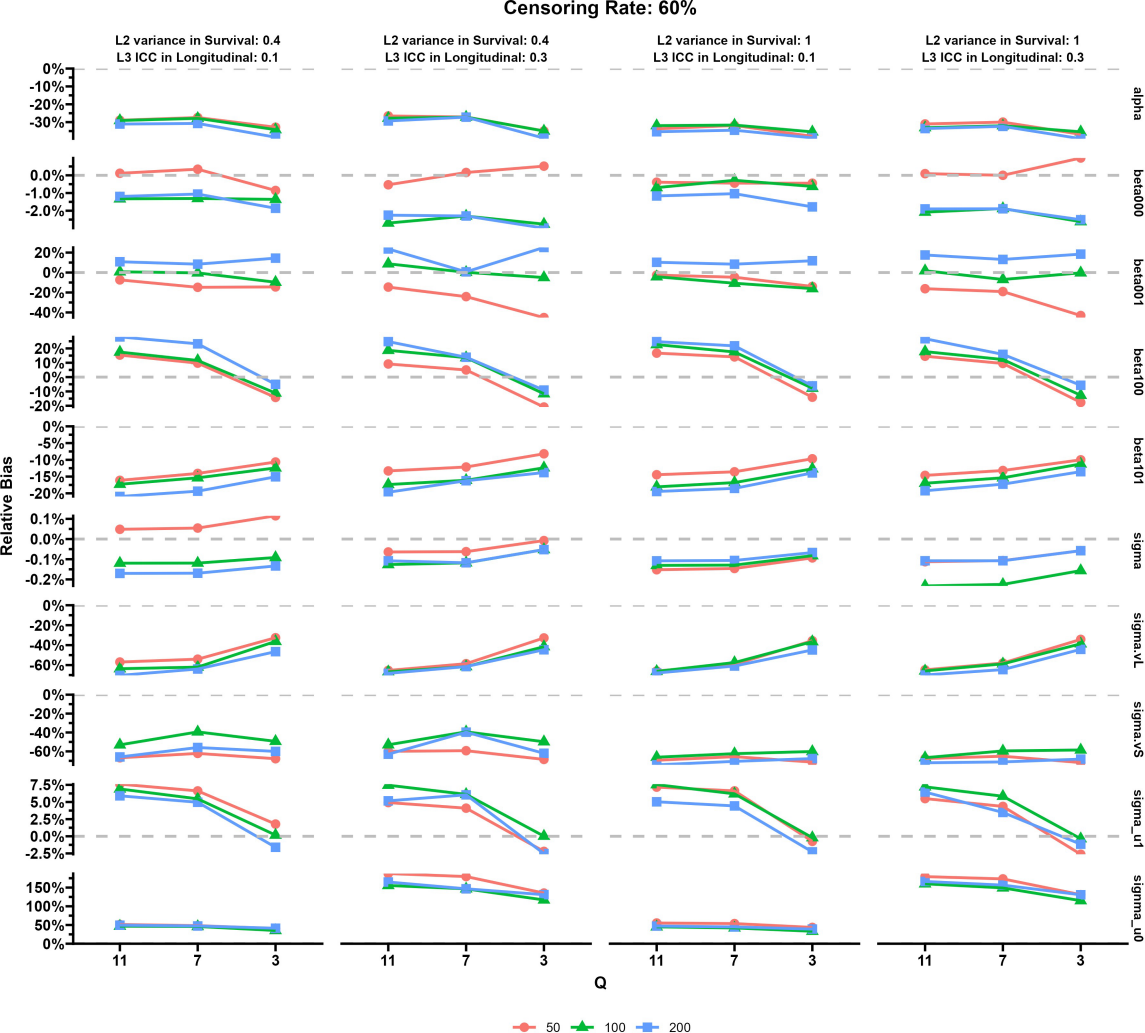
Simulation results: Comparison of results from MJM versus standard JM (ignoring the group level), censoring rate of 60%.

The results of the simulation study are displayed in Figures 3, 4, 5, 6 and more details including MESE and ESE can be found in the Supporting Information. Specifically, Figures 3 and 4 show the relative bias for parameters in the proposed MJM under the censoring rate of 20% and 60%, respectively. Figures 5 and 6 display the results of the comparison between MJM and standard JM ignoring the group‐level structure for the censoring rate of 20% and 60%, respectively. Supporting Information: Appendix 1 shows more details of the simulation results including MESE and ESE for the proposed MJM and Supporting Information: Appendix 2 shows more details of the simulation results for the comparison of the proposed MJM and the standard JM.

#### 5.3.1 Proposed MJM

Posterior samples were obtained by running three parallel MCMC chains, each with an adaption phase of 100 iterations and a burn‐in period of 4000 iterations. To reduce autocorrelation in the posterior samples, a thinning interval of 150 was applied. From each chain, 1500 posterior samples were retained, yielding a total of 4500 posterior samples for estimation and inference. Trace plots for a randomly selected example from the simulation studies are presented in Supporting Information: Appendix 4. In scenarios with 200 groups and 11 time intervals, it was not feasible to obtain 1500 posterior samples per chain in most replicates, even with the maximum allowed runtime on DRAC. Comparative analyses of estimation results using 1500 versus 200 posterior samples, conducted for sample size at the group level of 50 and 100, showed negligible differences in results. Therefore, for the eight scenarios involving 200 groups and 11 time intervals, 200 posterior samples per chain were used for estimation and inference.

When the censoring rate was 20%, the proposed MJM had acceptable performance in estimating both the treatment (intervention) effect $\beta_{101}$ and the association parameter α (see Figure 3). Estimation accuracy for these two parameters was generally better with small or median number of groups. The RB of the association parameter ($\hat{\alpha }$) increased when the variance at the group level in the survival submodel increased from 0.4 to 1 and when the censoring rate increased from 20% to 60% (compare Figures 3 and 4). The RB for the effect of the intervention (${\hat{\beta }}_{101}$) decreased when the group‐level ICC in the longitudinal submodel increased but increased when the censoring rate increased from 20% to 60% (compare Figures 3 and 4). Lastly, the effect of the number of time intervals used in the survival submodel did not appear to affect the estimates of the intervention effect on the longitudinal outcome. However, the RB of the indirect effect on the survival outcome ($\hat{\alpha }$) increased when the number of time intervals decreased to less than half of the number of repeated measurements.

For the remaining fixed effect parameters and the individual‐level random effects related to the change rate of the longitudinal outcome, the proposed MJM had good performance, especially for larger number of groups and higher number of time intervals. However, the change rate of the longitudinal outcome for the control group ($\beta_{100}$) tended to be underestimated when the group‐level ICC was lower (i.e., 0.1). The RB of ${\hat{\beta }}_{100}$ decreased when the censoring rate increased from 20% to 60%. In contrast, the RB for random effects were relatively large: the individual‐level random effects of the intercept ($\sigma_{u_{0\mathrm{jl}}}$) were overestimated, while the group‐level random effects ($v_{00l}$ and $g_{l}$) in both submodels were underestimated. Notably, the RB of the individual‐level random effects for intercept ($\sigma_{u_{0\mathrm{jl}}}$) increased substantially when the group‐level ICC in the longitudinal outcome increased from 0.1 to 0.3. The sample size at the group level primarily affects the RB of the difference in the baseline longitudinal outcome in the two groups (${\hat{\beta }}_{001}$) and the group‐level random effects in both submodels. Overall, the RB for the group‐level random effects and ${\hat{\beta }}_{001}$ was the lowest when sample size was 100. As the number of time intervals decreased, the RB for the ${\hat{\beta }}_{100}$ and ${\hat{\sigma }}_{u_{1\mathrm{jl}}}$ increased, especially when the number of intervals changed from 7 to 3.

#### 5.3.2 Impact of Ignoring the Group Level

First, standard JM that ignore group‐level structure are unable to capture variation in both the trajectories of the longitudinal outcome and the hazard of the event across groups. Figures 5 and 6 display the results using the proposed MJM and the standard JM. When censoring rate was 20%, the standard JM exhibited greater underestimation of the treatment (intervention) effect on the longitudinal outcome compared to the proposed MJM, particularly at a moderate number of groups (*n* = 50 or 100). However, when censoring rate was 60%, the standard JM had better performance in estimating the effect of the treatment (intervention) on the longitudinal outcome compared to the proposed MJM. Across all conditions, the indirect effect of the treatment (intervention) on the hazard of the event, mediated by the association between the longitudinal and survival outcomes, was consistently more underestimated by the standard JM than by the proposed MJM.

For other parameters in the analysis, the proposed MJM had better performance under most conditions compared to the standard JM ignored the group level. When the censoring rate was 20%, the difference of the longitudinal outcome at the baseline between the treatment (intervention) group and the control group was underestimated if the group level was ignored. The changing rate of the longitudinal outcome over time was significantly underestimated by about 70% if the group‐level was ignored. Additionally, the variation of the changing rate among individuals from the same group was also underestimated if the group‐level was not considered. Although the proposed MJM showed less overestimation of the variation of the baseline longitudinal outcome across individuals compared to the standard JM, the degree of overestimation remained considerable. The proposed MJM had similar or better performance in estimating the baseline longitudinal outcome in the control group ($\beta_{000}$) and the level 1 residuals ($\sigma_{\varepsilon }$) compared to the JM ignoring the group level.

In terms of standard error estimation for all fixed effect parameters in both the longitudinal and survival submodels, standard JMs tended to underestimate standard errors (reflected by smaller MESEs) compared to the proposed MJM. In contrast, the estimations of all fixed effect parameters had higher variation using the standard JM (as indicated by larger ESEs) compared to those from the proposed MJM.

In summary, this study demonstrates that failing to account for group‐level structures in joint modeling leads to substantial bias and increased variability in the estimation of key model parameters. The proposed Multilevel Joint Model (MJM) more accurately captures the distribution of treatment effects, between‐ and within‐group variances, and indirect effects mediated through longitudinal outcomes, particularly when censoring rates are moderate and the number of groups is not small. Although standard JM may perform better under high censoring rates for certain parameters, it consistently underestimates standard errors and compromises the accuracy of indirect effect estimates. Overall, these findings highlight the importance of incorporating group‐level effects in joint modeling to achieve more reliable and interpretable results in studies involving hierarchical or clustered data.

## 6 Discussion

This study proposed a multilevel joint model (MJM) for analyzing correlated longitudinal and survival data within a nested study design, addressed a gap in the literature on joint modeling for clustered data. The proposed MJM accommodates a three‐level hierarchy (repeated measurements within individuals, individuals within groups such as classrooms or schools), allows interactions across measurements, individual, and group levels, and provides a flexible framework suitable for evaluating group‐based interventions on both longitudinal markers and time‐to‐event outcomes—an approach particularly relevant for cluster randomized controlled trials (CRCT). We also investigated the consequences of ignoring group‐level variation when evaluating group‐based interventions in CRCTs using a joint modeling approach.

To demonstrate the practical utility and methodological advantages of the MJM, we applied the proposed model to data from the PAX intervention, which aimed to improve children's mental health. As highlighted in Tables 1, 2 and Figure 2, the distribution and progression of total difficulties over time underscored the necessity of models accounting for group‐level clustering, individual trajectories, and clinical diagnoses of mental disorders.

We compared three MJM specifications: (1) a linear trajectory MJM, (2) a quadratic trajectory MJM in which the linear slope of time varied by PAX, and (3) a quadratic trajectory MJM in which both the linear and quadratic slopes of time varied by PAX. Based on the DIC, the MJM with a linear trajectory provided the best fit to the data and was selected as the final model.

Our findings demonstrated that the PAX program significantly enhanced children's mental health from Grade 1 through Grade 5 and substantially reduced the risk of clinical mental disorder diagnoses up to 9 months after Grade‐12 graduation, compared to the control group. These results align with earlier studies demonstrating similar positive short‐term impacts of the PAX program [6, 7, 8]. However, the current study uniquely identifies both the direct and indirect long‐term protective effects on clinical mental health outcomes, a finding further supported by the MJM's ability to capture mediation pathways through the joint analysis of longitudinal and survival data. Notably, given that the clinical diagnostic data extended 4 years beyond the onset of the COVID‐19 pandemic, our results suggest that early childhood mental health interventions may yield enduring mental health benefits, even in the context of widespread societal stressors.

Furthermore, simulation studies summarized in Figures 5 and 6 corroborate the empirical findings and highlight the methodological pitfalls of ignoring the multilevel structure. Specifically, using standard joint models that overlook group‐level variation systematically underestimates the intervention effects on both longitudinal and survival outcomes. Moreover, the proposed MJM outperformed traditional models in accurately recovering both fixed and random effects parameters, as well as the intricate associations between trajectories of mental health difficulties and time‐to‐diagnosis outcomes. Additionally, ignoring the group‐level structure substantially underestimates standard errors for fixed‐effect parameters, inflating Type I errors and may falsely indicate statistical significance. These insights underscore the importance of adopting multilevel joint modeling to ensure robust inference for clustered or nested data, a common scenario in educational interventions and CRCTs [56].

Collectively, our results affirm the necessity and methodological advantages of the proposed MJM framework. By integrating real‐world applications with rigorous simulation analyses, the study demonstrates that accounting for the multilevel data structure is critical not only for unbiased estimation of intervention effects but also for accurately assessing variability and dynamic associations over time. Thus, the proposed MJM provides researchers with a powerful and flexible analytic tool, advancing both the methodological rigor and practical relevance of intervention evaluations in cluster‐based study designs.

Our findings further indicated gender, socioeconomic, and geographical disparities. Boys exhibited higher total difficulties at Grade 1 but had a slower increase over time and a lower subsequent risk of clinical mental disorder diagnosis through Grade 12. This gender pattern aligns with national data documenting rapid rising rates of depression and anxiety among adolescent girls compared to boys [57]. Similarly, students from lower socioeconomic status (SES) families presented with higher total difficulties but paradoxically had a lower likelihood of clinical diagnoses, likely reflecting systemic barriers to accessing mental health services, which are often not covered by public or employee benefits for low‐SES families [58]. Furthermore, students attending urban schools had lower total difficulties but a higher risk of clinical diagnoses compared to rural counterparts, likely reflecting the greater accessibility and availability of mental health services in urban regions [58]. Students receiving income assistance or services from child welfare agencies exhibited both higher baseline total difficulties and an elevated risk of clinical diagnoses.

Despite the proposed MJM having made substantial advances, several limitations warrant careful consideration. First, there is temporal misalignment between the SDQ measurements (through 4.5 years) and the longer follow‐up for mental disorder diagnoses: post‐4.5‐year survival risk is modeled with extrapolated SDQ trajectories, so long‐term inferences rely on these assumptions. Second, we only evaluated a linear‐time assumption for the SDQ trajectory; with richer longitudinal data, nonlinear specification (e.g., piecewise or spline‐based time) could be explored. We did examine a quadratic specification, but the three time points favored a linear‐time specification as a reasonable balance between bias and variance. Third, this study considered only the current‐value (CV) association structure linking the longitudinal and survival processes. While CV is a common and interpretable linkage, alternative association structures, including shared random effects (SRE), current slope (CS), and CV‐CS, can offer advantages in certain contexts by linking latent longitudinal tendencies or slopes to risk. However, an SRE association structure would not address one of our central research questions—assessing the indirect effect of PAX on mental disorder risk through its effect on SDQ. Moreover, the specification of such an association structure requires further methodological development to determine optimal strategies for linking longitudinal and survival components, particularly in multilevel settings. We did not perform formal comparisons among these structures here. Future work should investigate these alternatives with richer longitudinal data and use DIC to guide model selection.

Fourth, this research primarily focused on the total difficulties score and time‐to‐the first diagnosis for selected mental disorders. Future investigations should explore SDQ subscales and consider latent multilevel joint models with multiple longitudinal outcomes to capture broader aspects of mental health. Fifth, while the proposed MJM captured the intervention effects well, it tended to underestimate group‐level random effects in both longitudinal and survival submodels. Such underestimation is common in Bayesian multilevel modeling, where estimates naturally shrink toward global means, particular when group sizes are smaller or moderate [59]. Although the current study utilized a fixed group size of 30 and evaluated only three group‐level sample sizes informed by general recommendations from multilevel analysis literature, these guidelines may not adequately account for the complexities inherent in joint modeling frameworks. Consequently, while the chosen sample sizes were reasonable and methodologically justified, future studies would benefit from systematically investigating optimal sample sizes at each hierarchical level specifically tailored to the nuanced demands of MJMs. However, addressing optimal sample sizes requires first improving the computational efficiency of the MJM. In this study, the computational demands already approached the maximum available resources provided by the Digital Alliance of Canada (DRAC). The runtime for the selected MJM (linear trajectory with current value association) on PAC data was approximately 83 h on the MCHP server. Potential solutions for enhancing computational performance include (a) transitioning from JAGS to more efficient platforms such as C++, which offers significant advantages in computational speed due to matrix operations and flexible and faster numerical integration [16], (b) adopting alternative MCMC platforms such as Stan, which employ more efficient sampling algorithms (e.g., Hamiltonian Monte Carlo or No‐U‐Turn sampler) to reduce posterior sample autocorrelation [60].

Sixth, attrition due to migration posed limitations, as students leaving the province resulted in missing longitudinal data and censored survival times, without available information to directly identify these cases. This restricted out capacity to model informative censoring explicitly. Future research could mitigate this limitation by employing substantive model compatible imputation methods or joint model‐based imputation techniques, enhancing the validity of conclusions in the presence of missing data [61, 62].

In summary, this study introduced and rigorously evaluated a Multilevel Joint Model (MJM) for analyzing correlated longitudinal and survival outcomes within nested data structures, as commonly encountered in cluster randomized trials of school‐based interventions. By both empirical application to the PAX intervention and comprehensive simulation studies, the results showed that the proposed MJM outperformed standard joint modeling approaches that ignore grouping, yielding more accurate and unbiased estimates of treatment effects, standard errors, and associations over time. The analyses highlighted the long‐term, direct, and indirect mental health benefits of the PAX program while also uncovering meaningful disparities across gender, socioeconomic status, and geography. Importantly, neglecting group‐level variation led to systematic underestimation of intervention effects and standard errors, underscoring the methodological necessity for multilevel modeling in clustered educational or clinical research. Despite some limitations related to nonlinear trajectories, limited outcome scope, computational demands, and handling of missing data, this work advances both the methodological rigor and practical application of joint modeling, providing a powerful analytic framework for robust intervention evaluation in complex, clustered study designs.

## Funding

This work was supported by the Natural Sciences and Engineering Research Council of Canada (RGPIN‐2022‐04124) and Canadian Institute of Health Research (202003PJT).

## Ethics Statement

This study has been approved by the Health Research Ethics Board (HREB) of the University of Manitoba (H2023:333). Data access was provided by MCHP for use of data contained in the Manitoba Population Research Data Repository under project #2024–043. Data access approval was provided by the Provincial Health Research Privacy Committee (PHRPC No. P2023‐110), Manitoba Education, and Manitoba Families. In accordance with The Healthy Child Manitoba Act, no informed consents or permissions from parents are required. But a note was given to their parents that they can remove their child and/or come to the school to review the questionnaire in advance and then either allow their child to proceed or remove them.

## Conflicts of Interest

The authors declare no conflicts of interest.

## Supporting information


**Data S1:** Supporting Information.

## Data Availability

Data used in this article was derived from administrative health and social data as a secondary use. The data was provided under specific data sharing agreements only for approved use at MCHP. The original source data is not owned by the researchers or MCHP and as such cannot be provided to a public repository. The original data source and approval for use have been noted in the acknowledgments of the article. Where necessary, source data specific to this article or project may be reviewed at MCHP with the consent of the original data providers, along with the required privacy and ethical review bodies.

## References

[1] World Health Organization , “Mental Health of Adolescents,” 2024, https://www.who.int/news‐room/fact‐sheets/detail/adolescent‐mental‐health.

[2] N. Racine , B. A. McArthur , J. E. Cooke , R. Eirich , J. Zhu , and S. Madigan , “Global Prevalence of Depressive and Anxiety Symptoms in Children and Adolescents During COVID‐19: A Meta‐Analysis,” JAMA Pediatrics 175, no. 11 (2021): 1142–1150. 34369987 10.1001/jamapediatrics.2021.2482 PMC8353576

[3] M. E. Loades , E. Chatburn , N. Higson‐Sweeney , et al., “Rapid Systematic Review: The Impact of Social Isolation and Loneliness on the Mental Health of Children and Adolescents in the Context of COVID‐19,” Journal of the American Academy of Child and Adolescent Psychiatry 59, no. 11 (2020): 1218–1239. 32504808 10.1016/j.jaac.2020.05.009 PMC7267797

[4] J. Kim‐Cohen , A. Caspi , T. E. Moffitt , H. Harrington , B. J. Milne , and R. Poulton , “Prior Juvenile Diagnoses in Adults With Mental Disorder: Developmental Follow‐Back of a Prospective‐Longitudinal Cohort,” Archives of General Psychiatry 60, no. 7 (2003): 709–717. 12860775 10.1001/archpsyc.60.7.709

[5] J. P. Shonkoff , A. S. Garner , Committee on Psychosocial Aspects of Child and Family Health Adoption and Dependent Care and Section on Developmental and Behavioral Pediatrics C on EC , et al., “The Lifelong Effects of Early Childhood Adversity and Toxic Stress,” Pediatrics 129, no. 1 (2012): e232–e246. 22201156 10.1542/peds.2011-2663

[6] M. Brownell , T. Thomson , M. Chartier , et al., “The PAX Program in Manitoba: A Population‐Based Analysis of Children's Outcomes,” 2018, http://mchp‐appserv.cpe.umanitoba.ca/deliverablesList.html.

[7] D. Jiang , R. Santos , W. Josephson , T. Mayer , and L. Boyd , “A Comparison of Variable‐ and Person‐Oriented Approaches in Evaluating a Universal Preventive Intervention,” Prevention Science 19, no. 6 (2018): 738–747, 10.1007/s11121-018-0881-x. 29500615

[8] X. Jiang , “Validation Studies on a Screening Tool for Mental Disorder of Children and Youth in Canada and the Implication for Program Evaluation,” Master's thesis, University of Manitoba, 2021, http://hdl.handle.net/1993/35664.

[9] C. L. Faucett and D. C. Thomas , “Simultaneously Modelling Censored Survival Data and Repeatedly Measured Covariates: A Gibbs Sampling Approach,” Statistics in Medicine 15, no. 15 (1996): 1663–1685, 10.1002/(SICI)1097-0258(19960815)15:15<1663::AID-SIM294>3.0.CO;2-1. 8858789

[10] M. S. Wulfsohn and A. A. Tsisatis , “A Joint Model for Survival and Longitudinal Data Measured With Error,” Biometrics 53, no. 1 (1997): 330–339. 9147598

[11] S. Luo and J. Wang , “Bayesian Hierarchical Model for Multiple Repeated Measures and Survival Data: An Application to Parkinson's Disease,” Statistics in Medicine 33, no. 24 (2014): 4279–4291, 10.1002/sim.6228. 24935619 PMC4184935

[12] S. L. Brilleman , M. J. Crowther , M. Moreno‐Betancur , et al., “Joint Longitudinal and Time‐To‐Event Models for Multilevel Hierarchical Data,” Statistical Methods in Medical Research 28, no. 12 (2019): 3502–3515, 10.1177/0962280218808821. 30378472

[13] E. Kürüm , D. V. Nguyen , Y. Li , C. M. Rhee , K. Kalantar‐Zadeh , and D. Şentürk , “Multilevel Joint Modeling of Hospitalization and Survival in Patients on Dialysis,” Statistics 10, no. 1 (2021): e356, 10.1002/sta4.356. PMC9931182 36181392

[14] E. Kürüm , D. V. Nguyen , S. Banerjee , Y. Li , C. M. Rhee , and D. Şentürk , “A Bayesian Multilevel Time‐Varying Framework for Joint Modeling of Hospitalization and Survival in Patients on Dialysis,” Statistics in Medicine 41, no. 29 (2022): 5597–5611, 10.1002/sim.9582. 36181392 PMC9931182

[15] E. Kürüm , D. V. Nguyen , Q. Qian , S. Banerjee , C. M. Rhee , and D. Şentürk , “Spatiotemporal Multilevel Joint Modeling of Longitudinal and Survival Outcomes in End‐Stage Kidney Disease,” Lifetime Data Analysis 30 (2024): 827–852, 10.1007/s10985-024-09635-w. 39367291 PMC11502599

[16] D. Rizopoulos , “The R Package JMbayes for Fitting Joint Models for Longitudinal and Time‐To‐Event Data Using MCMC,” Journal of Statistical Software 72, no. 7 (2016): 1–46, 10.18637/jss.v072.i07.

[17] Y. Liu , M. Torabi , X. Zhang , and D. Jiang , “Improved Joint Modeling of Longitudinal and Survival Data Using a Poisson Regression Approach,” Statistical Methods & Applications 34 (2025): 325–344, 10.1007/s10260-025-00782-4.

[18] P. C. Austin , “A Tutorial on Multilevel Survival Analysis: Methods, Models and Applications,” International Statistical Review 85, no. 2 (2017): 185–203, 10.1111/insr.12214. 29307954 PMC5756088

[19] R. Ma , D. Krewski , and R. T. Burnett , “Random Effects Cox Models: A Poisson Modelling Approach,” Biometrika 90, no. 1 (2003): 157–169.

[20] A. Elghafghuf and H. Stryhn , “Robust Poisson Likelihood Estimation for Frailty Cox Models: A Simulation Study,” Communications in Statistics: Simulation and Computation 46, no. 4 (2017): 2907–2923, 10.1080/03610918.2015.1066806.

[21] M. J. Crowther , R. D. Riley , J. A. Staessen , J. Wang , F. Gueyffier , and P. C. Lambert , “Individual Patient Data Meta‐Analysis of Survival Data Using Poisson Regression Models,” BMC Medical Research Methodology 12 (2012): 12, 10.1186/1471-2288-12-34. 22443286 PMC3398853

[22] F. Hsieh , Y. K. Tseng , and J. L. Wang , “Joint Modeling of Survival and Longitudinal Data: Likelihood Approach Revisited,” Biometrics 62, no. 4 (2006): 1037–1043, 10.1111/j.1541-0420.2006.00570.x. 17156277

[23] D. Rizopoulos , Joint Models for Longitudinal and Survival Data With Application in R (CRC press, 2012).

[24] L. Ferrer , V. Rondeau , J. Dignam , T. Pickles , H. J. Dadda , and C. Proust‐Lima , “Joint Modelling of Longitudinal and Multi‐State Processes: Application to Clinical Progressions in Prostate Cancer,” Statistics in Medicine 35, no. 22 (2016): 3933–3948, 10.1002/sim.6972. 27090611 PMC5012926

[25] R. M. Elashoff , G. Li , and N. Li , “A Joint Model for Longitudinal Measurements and Survival Data in the Presence of Multiple Failure Types,” Biometrics 64, no. 3 (2008): 762–771, 10.1111/j.1541-0420.2007.00952.x. 18162112 PMC2751647

[26] W. Hu , G. Li , and N. Li , “A Bayesian Approach to Joint Analysis of Longitudinal Measurements and Competing Risks Failure Time Data,” Statistics in Medicine 28, no. 11 (2009): 1601–1619, 10.1002/sim.3562. 19308919 PMC3168565

[27] Y. Huang , G. Dagne , and L. Wu , “Bayesian Inference on Joint Models of HIV Dynamics for Time‐To‐Event and Longitudinal Data With Skewness and Covariate Measurement Errors,” Statistics in Medicine 30, no. 24 (2011): 2930–2946, 10.1002/sim.4321. 21805486

[28] L. Liu , X. Huang , and J. O'Quigley , “Analysis of Longitudinal Data in the Presence of Informative Observational Times and a Dependent Terminal Event, With Application to Medical Cost Data,” Biometrics 64, no. 3 (2008): 950–958, 10.1111/j.1541-0420.2007.00954.x. 18162110

[29] L. Liu , J. Z. Ma , and J. O'Quigley , “Joint Analysis of Multi‐Level Repeated Measures Data and Survival: An Application to the End Stage Renal Disease (ESRD) Data,” Statistics in Medicine 27, no. 27 (2008): 5679–5691, 10.1002/sim.3392. 18693300

[30] G. Rodriguez , “Multilevel Generalized Linear Models,” in Handbook of Multilevel Analysis (Springer New York, 2008), 335–376, 10.1007/978-0-387-73186-5.

[31] A. Elghafghuf , H. Stryhn , and C. Waldner , “A Cross‐Classified and Multiple Membership Cox Model Applied to Calf Mortality Data,” Preventive Veterinary Medicine 115, no. 1–2 (2014): 29–38, 10.1016/j.prevetmed.2014.03.012. 24703248 PMC7114250

[32] G. Papageorgiou , K. Mauff , A. Tomer , and D. Rizopoulos , “An Overview of Joint Modeling of Time‐To‐Event and Longitudinal Outcomes,” Annual Review of Statistics and Its Application 6 (2019): 223–240, 10.1146/annurev-statistics-030718-105048.

[33] T. Donovan and R. M. Mickey , “MCMC Diagnostic Approaches,” in Bayesian Statistics for Beginners: A Step‐By‐Step Approach, 1st ed. (Oxford University Press, 2019), 10.1093/oso/9780198841296.001.0001.

[34] V. Roy , “Convergence Diagnostics for Markov Chain Monte Carlo,” Annual Review of Statistics and Its Application 7 (2019): 387–412, http://arxiv.org/abs/1909.11827.

[35] W. A. Link and M. J. Eaton , “On Thinning of Chains in MCMC,” Methods in Ecology and Evolution 3, no. 1 (2012): 112–115, 10.1111/j.2041-210X.2011.00131.x.

[36] D. Alvares and F. J. Rubio , “A Tractable Bayesian Joint Model for Longitudinal and Survival Data,” Statistics in Medicine 40, no. 19 (2021): 4213–4229, 10.1002/sim.9024. 34114254

[37] D. Alvares , E. Lázaro , V. Gómez‐Rubio , and C. Armero , “Bayesian Survival Analysis With BUGS,” Statistics in Medicine 40, no. 12 (2021): 2975–3020, 10.1002/sim.8933. 33713474

[38] D. Lunn , C. Jackson , N. Best , A. Thomas , and D. Spiegelhalter , The BUGS Book. A Practical Introduction to Bayesian Analysis (Chapman Hall, 2013).

[39] H. Goldstein , Multilevel Statistical Models, vol. 922, 4th ed. (John Wiley & Sons, 2011).

[40] R. Goodman , “The Strengths and Difficulties Questionnaire: A Research Note,” Journal of Child Psychology and Psychiatry 38, no. 5 (1997): 581–586, 10.1111/j.1469-7610.1997.tb01545.x. 9255702

[41] D. Chateau , C. Metge , and R. A. Soodeen , “Learning from the Census: The Socio‐Economic Factor Index (SEFI) and Health Outcomes in Manitoba,” 2012. 10.1007/BF03403825 PMC6973861 23618067

[42] D. Jiang , “A Comparison of Variable‐ and Person‐Oriented Approaches in Evaluating a Universal Preventive Intervention,” 2018. 10.1007/s11121-018-0881-x 29500615

[43] I. G. Katsantonis , “Development of Internalizing Mental Health Symptoms From Early Childhood to Late Adolescence,” European Journal of Investigation in Health, Psychology and Education 14, no. 8 (2024): 2404–2416, 10.3390/ejihpe14080159. 39194953 PMC11353613

[44] F. McNicholas , B. Gavin , R. Sellers , et al., “Examining the Mental Health Trajectories of Children and Adolescents: A Cross‐Cohort Analysis,” Psychological Medicine 54, no. 15 (2024): 4062–4070, 10.1017/S0033291724001624. PMC11650181 39564750

[45] S. L. Brilleman , R. Wolfe , M. Moreno‐Betancur , and M. J. Crowther , “Simulating Survival Data Using the Simsurv R Package,” Journal of Statistical Software 97, no. 3 (2021): 1–27, 10.18637/jss.v097.i03.

[46] M. J. Crowther and P. C. Lambert , “Simulating Biologically Plausible Complex Survival Data,” Statistics in Medicine 32, no. 23 (2013): 4118–4134, 10.1002/sim.5823. 23613458

[47] D. Bassiri , Large and Small Sample Properties of Maximum Likelihood Estimates for the Hierarchical Linear Model (Michigan State University. Department of Counseling, Educational Psychology, 1988).

[48] C. A. Scherbaum and J. M. Ferreter , “Estimating Statistical Power and Required Sample Sizes for Organizational Research Using Multilevel Modeling,” Organizational Research Methods 12, no. 2 (2009): 347–367.

[49] J. Hox , M. Moerbeek , and R. de van Schoot , Multilevel Analysis: Techniques and Applications, Third ed. (Routledge, 2017), 10.4324/9781315650982.

[50] J. Hox , “Multilevel Modeling: When and Why,” in Classification, Data Analysis, and Data Highways: Proceedings of the 21st Annual Conference of the Gesellschaft Für Klassifikation EV, University of Potsdam, March 12–14, 1997 (Springer, 1998), 147–154.

[51] M. A. Hasan , “Program Evaluation with Multilevel Longitudinal Data: Evidence from Simulation Study and Cluster Randomized Controlled Trial,” Master's Thesis, University of Manitoba, 2021.

[52] E. Lee and S. Hong , “Adequate Sample Sizes for a Three‐Level Growth Model,” Frontiers in Psychology 12 (2021): 12, 10.3389/fpsyg.2021.685496. PMC8282204 34276510

[53] P. Davis and A. Scott , “The Effect of Interviewer Variance on Domain Comparisons,” Survey Methodology 21 (1995): 99–106.

[54] A. Jahn‐Eimermacher , K. Ingel , and A. Schneider , “Sample Size in Cluster‐Randomized Trials With Time to Event as the Primary Endpoint,” Statistics in Medicine 32, no. 5 (2013): 739–751, 10.1002/sim.5548. 22865817

[55] S. Kalia , “On the Estimation of Intracluster Correlation for Time‐To‐Event in Cluster Randomized Trials,” Master of Science, The University of Western Ontario, 2015, https://ir.lib.uwo.ca/etdhttps://ir.lib.uwo.ca/etd/3213.

[56] N. Hyun , A. E. Idu , A. J. Cook , and J. F. Bobb , “Increased Risk of Type I Errors for Detecting Heterogeneity of Treatment Effects in Cluster‐Randomized Trials Using Mixed‐Effect Models,” 2024. 10.1186/s12874-025-02744-6 PMC12888205 41526818

[57] W. Craig , W. Pickett , and M. King , The Health of Canadian Youth: Findings From the Health Behaviour in School‐Aged Children Study (Public Health Agency of Canada, 2020), https://www.canada.ca/content/dam/phac‐aspc/documents/services/publications/science‐research‐data/hbsc/health‐behaviour‐school‐aged‐children‐study‐report.pdf.

[58] Health Commission of Canada M , “Mental Health and the High Cost of Living—Policy Brief,” 2023.

[59] R. McElreath , Statistical Rethinking: A Bayesian Course With Examples in R and Stan, 2nd ed. (CRC Press, 2020).

[60] Stan Development Team , “Stan Modeling Language,” 2019, https://mc‐stan.org/.

[61] J. W. Bartlett , S. R. Seaman , I. R. White , and J. R. Carpenter , “Multiple Imputation of Covariates by Fully Conditional Specification: Accommodating the Substantive Model,” Statistical Methods in Medical Research 24, no. 4 (2015): 462–487, 10.1177/0962280214521348. 24525487 PMC4513015

[62] B. Hu , L. Li , and T. Greene , “Joint Multiple Imputation for Longitudinal Outcomes and Clinical Events That Truncate Longitudinal Follow‐Up,” Statistics in Medicine 35, no. 17 (2016): 2991–3006, 10.1002/sim.6590. 26179943 PMC4714958

