# Supplementary material for: A Bayesian Multilevel Joint Modeling of Longitudinal and Survival Outcomes in Cluster Randomized Controlled Trial Studies
Source: Stat Med. 2026 Jan 22;45(1-2):e70385. doi: 10.1002/sim.70385 (PMC12824832; doi:10.1002/sim.70385)
Supplement: Supplementary file 1 — Data S1: Supporting Information. [file SIM-45-0-s001.docx]

SUPPLEMENTARY MATERIALS

# Appendix 1 Simulation studies to evaluate performance of the proposed multilevel joint model (MJM)

Table A 1‑1 Results of the proposed MJM censoring rate 20%, ICC at the level 3 in the longitudinal submodel = 0.1, group-level variance in the survival submodel = 0.4

|  | L3 ICC in longitudinal outcome = 0.1; variance at L2 in survival = 0.4 | | | | | | | | | | | | | | | |
| --- | --- | --- | --- | --- | --- | --- | --- | --- | --- | --- | --- | --- | --- | --- | --- | --- |
|  | Parameter | Mean | Bias | RB | MESE | ESE | Mean | Bias | RB | MESE | ESE | Mean | Bias | RB | MESE | ESE |
|  |  | n = 50 | | | | | n = 100 | | | | | n = 200 | | | | |
| K=11 | α (-0.2) | -0.168 | 0.032 | -15.78% | 0.008 | 0.007 | -0.165 | 0.035 | -17.63% | 0.006 | 0.007 | -0.161 | 0.039 | -19.67% | 0.004 | 0.003 |
|  | β_000 (16) | 16.104 | 0.104 | 0.65% | 0.433 | 0.139 | 15.929 | -0.071 | -0.45% | 0.300 | 0.364 | 15.946 | -0.054 | -0.34% | 0.184 | 0.279 |
|  | β_100 (-0.8) | -0.611 | 0.189 | -23.68% | 0.163 | 0.126 | -0.597 | 0.203 | -25.41% | 0.120 | 0.319 | -0.607 | 0.193 | -24.18% | 0.086 | 0.085 |
|  | β_001 (1.6) | 1.401 | -0.199 | -12.46% | 0.298 | 0.370 | 1.274 | -0.326 | -20.35% | 0.216 | 0.374 | 1.615 | 0.015 | 0.92% | 0.148 | 0.481 |
|  | β_101 (-2) | -1.811 | 0.189 | -9.45% | 0.224 | 0.155 | -1.720 | 0.280 | -14.01% | 0.159 | 0.212 | -1.698 | 0.302 | -15.10% | 0.112 | 0.088 |
|  | σ^2_(u_0) (10) | 16.046 | 6.046 | 60.46% | 1.155 | 2.440 | 13.981 | 3.981 | 39.81% | 0.766 | 0.153 | 14.381 | 4.381 | 43.81% | 0.547 | 0.512 |
|  | σ_u_0 u_1 (0) | -0.943 | -0.943 | NA | 0.622 | 0.398 | -0.761 | -0.761 | NA | 0.427 | 0.137 | -0.817 | -0.817 | NA | 0.304 | 0.403 |
|  | σ^2_(u_1 ) (10) | 10.246 | 0.246 | 2.46% | 0.602 | 0.252 | 10.248 | 0.248 | 2.48% | 0.433 | 0.589 | 9.995 | -0.005 | -0.05% | 0.302 | 0.159 |
|  | σ_(v_L ) (2.2361) | 1.062 | -1.175 | -52.53% | 0.373 | 0.363 | 1.108 | -1.128 | -50.45% | 0.235 | 0.064 | 0.788 | -1.448 | -64.76% | 0.139 | 0.137 |
|  | σ_(v_S ) (0.6325) | 0.274 | -0.359 | -56.73% | 0.092 | 0.068 | 0.262 | -0.371 | -58.62% | 0.055 | 0.090 | 0.192 | -0.441 | -69.71% | 0.030 | 0.034 |
|  | σ_ε (5.7446) | 5.700 | -0.045 | -0.78% | 0.044 | 0.041 | 5.725 | -0.020 | -0.35% | 0.032 | 0.016 | 5.731 | -0.014 | -0.24% | 0.023 | 0.010 |
| K=7 | α (-0.2) | -0.175 | 0.025 | -12.28% | 0.008 | 0.004 | -0.169 | 0.031 | -15.41% | 0.006 | 0.004 | -0.165 | 0.035 | -17.63% | 0.004 | 0.002 |
|  | β_000 (16) | 16.307 | 0.307 | 1.92% | 0.413 | 0.145 | 16.011 | 0.011 | 0.10% | 0.285 | 0.423 | 15.909 | -0.091 | -0.57% | 0.170 | 0.279 |
|  | β_100 (-0.8) | -0.507 | 0.293 | -36.65% | 0.160 | 0.110 | -0.456 | 0.344 | -42.96% | 0.117 | 0.300 | -0.474 | 0.326 | -40.76% | 0.084 | 0.074 |
|  | β_001 (1.6) | 1.044 | -0.556 | -34.74% | 0.310 | 0.211 | 1.126 | -0.474 | -29.64% | 0.222 | 0.278 | 1.618 | 0.018 | 1.12% | 0.153 | 0.452 |
|  | β_101 (-2) | -1.744 | 0.256 | -12.79% | 0.223 | 0.108 | -1.720 | 0.280 | -14.00% | 0.157 | 0.276 | -1.683 | 0.317 | -15.84% | 0.111 | 0.068 |
|  | σ^2_(u_0) (10) | 15.171 | 5.171 | 51.71% | 1.117 | 2.235 | 13.225 | 3.225 | 32.25% | 0.737 | 0.164 | 13.933 | 3.933 | 39.33% | 0.536 | 0.470 |
|  | σ_u_0 u_1 (0) | -0.835 | -0.835 | NA | 0.611 | 0.521 | -0.722 | -0.722 | NA | 0.418 | 0.115 | -0.822 | -0.822 | NA | 0.299 | 0.382 |
|  | σ^2_(u_1 ) (10) | 10.048 | 0.048 | 0.48% | 0.591 | 0.188 | 9.953 | -0.047 | -0.47% | 0.419 | 0.488 | 9.698 | -0.302 | -3.02% | 0.293 | 0.135 |
|  | σ_(v_L ) (2.2361) | 1.256 | -0.981 | -43.85% | 0.337 | 0.386 | 1.303 | -0.933 | -41.73% | 0.207 | 0.040 | 0.987 | -1.249 | -55.85% | 0.122 | 0.080 |
|  | σ_(v_S ) (0.6325) | 0.301 | -0.331 | -52.39% | 0.080 | 0.107 | 0.278 | -0.354 | -56.04% | 0.048 | 0.052 | 0.225 | -0.407 | -64.35% | 0.030 | 0.045 |
|  | σ_ε (5.7446) | 5.706 | -0.038 | -0.67% | 0.044 | 0.040 | 5.727 | -0.018 | -0.31% | 0.032 | 0.015 | 5.735 | -0.010 | -0.17% | 0.023 | 0.010 |
| K=3 | α (-0.2) | -0.153 | 0.047 | -23.42% | 0.004 | 0.003 | -0.155 | 0.045 | -22.31% | 0.006 | 0.005 | -0.153 | 0.047 | -23.42% | 0.004 | 0.003 |
|  | β_000 (16) | 15.634 | -0.366 | -2.29% | 0.169 | 0.247 | 15.702 | -0.298 | -1.86% | 0.260 | 0.309 | 15.634 | -0.366 | -2.29% | 0.169 | 0.247 |
|  | β_100 (-0.8) | -0.031 | 0.769 | -96.11% | 0.079 | 0.072 | -0.011 | 0.789 | -98.58% | 0.110 | 0.302 | -0.031 | 0.769 | -96.11% | 0.079 | 0.072 |
|  | β_001 (1.6) | 1.521 | -0.079 | -4.94% | 0.165 | 0.396 | 1.070 | -0.530 | -33.11% | 0.241 | 0.200 | 1.521 | -0.079 | -4.94% | 0.165 | 0.396 |
|  | β_101 (-2) | -1.723 | 0.277 | -13.86% | 0.105 | 0.091 | -1.755 | 0.245 | -12.25% | 0.149 | 0.280 | -1.723 | 0.277 | -13.86% | 0.105 | 0.091 |
|  | σ^2_(u_0) (10) | 12.323 | 2.323 | 23.23% | 0.492 | 0.488 | 11.814 | 1.814 | 18.14% | 0.678 | 0.179 | 12.323 | 2.323 | 23.23% | 0.492 | 0.488 |
|  | σ_u_0 u_1 (0) | 0.264 | 0.264 | NA | 0.267 | 0.292 | 0.343 | 0.343 | NA | 0.375 | 0.109 | 0.264 | 0.264 | NA | 0.267 | 0.292 |
|  | σ^2_(u_1 ) (10) | 8.101 | -1.899 | -18.99% | 0.243 | 0.069 | 8.321 | -1.679 | -16.79% | 0.349 | 0.415 | 8.101 | -1.899 | -18.99% | 0.243 | 0.069 |
|  | σ_(v_L ) (2.2361) | 1.372 | -0.864 | -38.65% | 0.117 | 0.063 | 1.587 | -0.650 | -29.05% | 0.182 | 0.041 | 1.372 | -0.864 | -38.65% | 0.117 | 0.063 |
|  | σ_(v_S ) (0.6325) | 0.269 | -0.364 | -57.50% | 0.026 | 0.023 | 0.318 | -0.315 | -49.79% | 0.041 | 0.032 | 0.269 | -0.364 | -57.50% | 0.026 | 0.023 |
|  | σ_ε (5.7446) | 5.768 | 0.023 | 0.40% | 0.023 | 0.008 | 5.758 | 0.013 | 0.23% | 0.032 | 0.015 | 5.768 | 0.023 | 0.40% | 0.023 | 0.008 |

Table A 1‑2 Results of the proposed MJM censoring rate 20%, ICC at the level 3 in the longitudinal submodel = 0.1, group-level variance in the survival submodel = 1

|  | L3 ICC in longitudinal outcome = 0.1; variance at L2 in survival = 1 | | | | | | | | | | | | | | | |
| --- | --- | --- | --- | --- | --- | --- | --- | --- | --- | --- | --- | --- | --- | --- | --- | --- |
|  | Parameter | Mean | Bias | RB | MESE | ESE | Mean | Bias | RB | MESE | ESE | Mean | Bias | RB | MESE | ESE |
|  |  | n = 50 | | | | | n = 100 | | | | | n = 200 | | | | |
| K=11 | α (-0.2) | -0.149 | 0.051 | -25.72% | 0.007 | 0.004 | -0.145 | 0.055 | -27.57% | 0.005 | 0.007 | -0.146 | 0.054 | -26.85% | 0.004 | 0.002 |
|  | β_000 (16) | 16.183 | 0.183 | 1.15% | 0.510 | 0.125 | 15.879 | -0.121 | -0.75% | 0.317 | 0.220 | 15.890 | -0.110 | -0.69% | 0.183 | 0.228 |
|  | β_100 (-0.8) | -0.544 | 0.256 | -32.00% | 0.164 | 0.162 | -0.563 | 0.237 | -29.63% | 0.121 | 0.211 | -0.613 | 0.187 | -23.33% | 0.088 | 0.114 |
|  | β_001 (1.6) | 1.199 | -0.401 | -25.04% | 0.303 | 0.243 | 1.282 | -0.318 | -19.86% | 0.223 | 0.337 | 1.661 | 0.061 | 3.84% | 0.149 | 0.376 |
|  | β_101 (-2) | -1.787 | 0.213 | -10.64% | 0.228 | 0.052 | -1.739 | 0.261 | -13.06% | 0.161 | 0.208 | -1.684 | 0.316 | -15.79% | 0.114 | 0.044 |
|  | σ^2_(u_0) (10) | 16.675 | 6.675 | 66.75% | 1.189 | 1.539 | 14.220 | 4.220 | 42.20% | 0.778 | 0.865 | 14.387 | 4.387 | 43.87% | 0.553 | 0.618 |
|  | σ_u_0 u_1 (0) | -1.357 | -1.357 | NA | 0.646 | 0.460 | -0.675 | -0.675 | NA | 0.432 | 0.429 | -0.710 | -0.710 | NA | 0.308 | 0.287 |
|  | σ^2_(u_1 ) (10) | 10.261 | 0.261 | 2.61% | 0.604 | 0.183 | 10.172 | 0.172 | 1.72% | 0.430 | 0.458 | 10.071 | 0.071 | 0.71% | 0.306 | 0.116 |
|  | σ_(v_L ) (2.2361) | 1.156 | -1.080 | -48.29% | 0.459 | 0.574 | 1.206 | -1.030 | -46.06% | 0.254 | 0.141 | 0.818 | -1.418 | -63.41% | 0.141 | 0.070 |
|  | σ_(v_S ) (0.6325) | 0.265 | -0.735 | -73.54% | 0.093 | 0.065 | 0.345 | -0.655 | -65.53% | 0.067 | 0.040 | 0.267 | -0.733 | -73.32% | 0.038 | 0.032 |
|  | σ_ε (5.7446) | 5.695 | -0.050 | -0.86% | 0.044 | 0.030 | 5.726 | -0.018 | -0.32% | 0.032 | 0.025 | 5.740 | -0.005 | -0.09% | 0.023 | 0.007 |
| K=7 | α (-0.2) | -0.169 | 0.031 | -15.41% | 0.006 | 0.004 | -0.153 | 0.047 | -23.46% | 0.005 | 0.006 | -0.153 | 0.047 | -23.59% | 0.004 | 0.001 |
|  | β_000 (16) | 16.011 | 0.011 | 0.07% | 0.286 | 0.422 | 15.957 | -0.043 | -0.27% | 0.307 | 0.207 | 15.940 | -0.060 | -0.38% | 0.177 | 0.297 |
|  | β_100 (-0.8) | -0.456 | 0.344 | -42.94% | 0.117 | 0.300 | -0.448 | 0.352 | -44.01% | 0.119 | 0.242 | -0.489 | 0.311 | -38.86% | 0.085 | 0.100 |
|  | β_001 (1.6) | 1.126 | -0.474 | -29.65% | 0.223 | 0.279 | 1.124 | -0.476 | -29.74% | 0.230 | 0.385 | 1.573 | -0.027 | -1.67% | 0.153 | 0.464 |
|  | β_101 (-2) | -1.719 | 0.281 | -14.05% | 0.158 | 0.276 | -1.725 | 0.275 | -13.76% | 0.160 | 0.281 | -1.707 | 0.293 | -14.65% | 0.113 | 0.037 |
|  | σ^2_(u_0) (10) | 13.226 | 3.226 | 32.26% | 0.737 | 0.165 | 13.597 | 3.597 | 35.97% | 0.756 | 0.734 | 13.832 | 3.832 | 38.32% | 0.538 | 0.656 |
|  | σ_u_0 u_1 (0) | -0.722 | -0.722 | NA | 0.418 | 0.115 | -0.666 | -0.666 | NA | 0.424 | 0.429 | -0.632 | -0.632 | NA | 0.304 | 0.294 |
|  | σ^2_(u_1 ) (10) | 9.952 | -0.048 | -0.48% | 0.418 | 0.487 | 9.925 | -0.075 | -0.75% | 0.418 | 0.475 | 9.855 | -0.145 | -1.45% | 0.298 | 0.080 |
|  | σ_(v_L ) (2.2361) | 1.303 | -0.933 | -41.72% | 0.207 | 0.039 | 1.400 | -0.836 | -37.37% | 0.225 | 0.078 | 1.041 | -1.195 | -53.45% | 0.130 | 0.040 |
|  | σ_(v_S ) (0.6325) | 0.278 | -0.354 | -56.03% | 0.048 | 0.052 | 0.416 | -0.584 | -58.37% | 0.065 | 0.072 | 0.331 | -0.669 | -66.90% | 0.038 | 0.037 |
|  | σ_ε (5.7446) | 5.727 | -0.017 | -0.30% | 0.032 | 0.015 | 5.730 | -0.015 | -0.25% | 0.032 | 0.025 | 5.744 | -0.001 | -0.01% | 0.023 | 0.008 |
| K=3 | α (-0.2) | -0.152 | 0.048 | -23.96% | 0.008 | 0.009 | -0.156 | 0.044 | -21.94% | 0.006 | 0.006 | -0.149 | 0.051 | -25.55% | 0.004 | 0.001 |
|  | β_000 (16) | 15.901 | -0.099 | -0.62% | 0.360 | 0.263 | 15.807 | -0.193 | -1.21% | 0.264 | 0.220 | 15.666 | -0.334 | -2.09% | 0.178 | 0.131 |
|  | β_100 (-0.8) | -0.054 | 0.746 | -93.23% | 0.153 | 0.204 | -0.093 | 0.707 | -88.32% | 0.112 | 0.257 | -0.114 | 0.686 | -85.81% | 0.081 | 0.097 |
|  | β_001 (1.6) | 1.019 | -0.581 | -36.34% | 0.337 | 0.467 | 0.902 | -0.698 | -43.60% | 0.247 | 0.411 | 1.512 | -0.088 | -5.53% | 0.168 | 0.187 |
|  | β_101 (-2) | -1.815 | 0.185 | -9.25% | 0.216 | 0.185 | -1.743 | 0.257 | -12.85% | 0.153 | 0.341 | -1.701 | 0.299 | -14.96% | 0.107 | 0.058 |
|  | σ^2_(u_0) (10) | 14.712 | 4.712 | 47.12% | 1.090 | 1.199 | 12.351 | 2.351 | 23.51% | 0.699 | 0.826 | 12.381 | 2.381 | 23.81% | 0.495 | 0.448 |
|  | σ_u_0 u_1 (0) | -0.514 | -0.514 | NA | 0.591 | 0.637 | 0.310 | 0.310 | NA | 0.390 | 0.288 | 0.286 | 0.286 | NA | 0.274 | 0.267 |
|  | σ^2_(u_1 ) (10) | 8.756 | -1.244 | -12.44% | 0.517 | 0.149 | 8.569 | -1.431 | -14.31% | 0.358 | 0.540 | 8.437 | -1.563 | -15.63% | 0.254 | 0.038 |
|  | σ_(v_L ) (2.2361) | 1.510 | -0.726 | -32.47% | 0.268 | 0.323 | 1.603 | -0.634 | -28.33% | 0.186 | 0.067 | 1.470 | -0.766 | -34.27% | 0.124 | 0.089 |
|  | σ_(v_S ) (0.6325) | 0.426 | -0.574 | -57.39% | 0.073 | 0.084 | 0.479 | -0.521 | -52.07% | 0.054 | 0.047 | 0.412 | -0.588 | -58.81% | 0.034 | 0.018 |
|  | σ_ε (5.7446) | 5.724 | -0.021 | -0.36% | 0.045 | 0.030 | 5.756 | 0.011 | 0.20% | 0.032 | 0.030 | 5.770 | 0.025 | 0.44% | 0.023 | 0.009 |

Table A 1‑3 Results of the proposed MJM censoring rate 20%, ICC at the level 3 in the longitudinal submodel = 0.3, group-level variance in the survival submodel = 0.4

|  | L3 ICC in longitudinal outcome = 0.3; variance at L2 in survival = 0.4 | | | | | | | | | | | | | | | |
| --- | --- | --- | --- | --- | --- | --- | --- | --- | --- | --- | --- | --- | --- | --- | --- | --- |
|  | Parameter | Mean | Bias | RB | MESE | ESE | Mean | Bias | RB | MESE | ESE | Mean | Bias | RB | MESE | ESE |
|  |  | n = 50 | | | | | n = 100 | | | | | n = 200 | | | | |
| K=11 | α (-0.2) | -0.162 | 0.038 | -19.03% | 0.007 | 0.006 | -0.164 | 0.036 | -17.89% | 0.005 | 0.004 | -0.164 | 0.036 | -18.22% | 0.004 | 0.002 |
|  | β_000 (16) | 16.126 | 0.126 | 0.79% | 0.897 | 0.252 | 15.645 | -0.355 | -2.216% | 0.557 | 0.491 | 15.859 | -0.141 | -0.88% | 0.319 | 0.414 |
|  | β_100 (-0.8) | -0.521 | 0.279 | -34.87% | 0.164 | 0.122 | -0.519 | 0.281 | -35.15% | 0.123 | 0.197 | -0.640 | 0.160 | -19.96% | 0.088 | 0.099 |
|  | β_001 (1.6) | 1.106 | -0.494 | -30.86% | 0.359 | 1.003 | 1.474 | -0.126 | -7.85% | 0.269 | 0.055 | 1.667 | 0.067 | 4.22% | 0.175 | 0.543 |
|  | β_101 (-2) | -1.820 | 0.180 | -8.99% | 0.227 | 0.106 | -1.827 | 0.173 | -8.65% | 0.163 | 0.178 | -1.600 | 0.400 | -19.98% | 0.114 | 0.050 |
|  | σ^2_(u_0) (10) | 26.804 | 16.804 | 168.04% | 1.581 | 3.518 | 24.595 | 14.595 | 145.95% | 1.075 | 0.152 | 25.183 | 15.183 | 151.83% | 0.772 | 1.973 |
|  | σ_u_0 u_1 (0) | -1.622 | -1.622 | NA | 0.762 | 1.028 | -1.555 | -1.555 | NA | 0.539 | 0.223 | -1.496 | -1.496 | NA | 0.380 | 0.029 |
|  | σ^2_(u_1 ) (10) | 10.408 | 0.408 | 4.08% | 0.611 | 0.168 | 10.358 | 0.358 | 3.58% | 0.442 | 0.476 | 9.992 | -0.008 | -0.08% | 0.304 | 0.336 |
|  | σ_(v_L ) (2.2361) | 2.404 | -1.839 | -43.33% | 0.766 | 0.364 | 2.342 | -1.901 | -44.81% | 0.428 | 0.291 | 1.757 | -2.486 | -58.59% | 0.238 | 0.107 |
|  | σ_(v_S ) (0.6325) | 0.244 | -0.389 | -61.49% | 0.086 | 0.044 | 0.280 | -0.353 | -55.80% | 0.057 | 0.033 | 0.213 | -0.419 | -66.32% | 0.033 | 0.014 |
|  | σ_ε (5.7446) | 5.693 | -0.052 | -0.90% | 0.044 | 0.023 | 5.717 | -0.027 | -0.48% | 0.032 | 0.025 | 5.742 | -0.003 | -0.05% | 0.023 | 0.005 |
| K=7 | α (-0.2) | -0.164 | 0.036 | -17.86% | 0.007 | 0.006 | -0.167 | 0.033 | -16.46% | 0.005 | 0.003 | -0.165 | 0.035 | -17.26% | 0.004 | 0.001 |
|  | β_000 (16) | 16.188 | 0.188 | 1.17% | 0.701 | 0.152 | 15.629 | -0.371 | -2.320% | 0.460 | 0.437 | 15.894 | -0.106 | -0.663% | 0.310 | 0.383 |
|  | β_100 (-0.8) | -0.410 | 0.390 | -48.72% | 0.161 | 0.106 | -0.368 | 0.432 | -53.94% | 0.119 | 0.201 | -0.511 | 0.289 | -36.07% | 0.086 | 0.091 |
|  | β_001 (1.6) | 0.904 | -0.696 | -43.48% | 0.357 | 0.936 | 1.497 | -0.103 | -6.42% | 0.271 | 0.600 | 1.611 | 0.011 | 0.66% | 0.181 | 0.698 |
|  | β_101 (-2) | -1.773 | 0.227 | -11.34% | 0.226 | 0.078 | -1.815 | 0.185 | -9.24% | 0.160 | 0.231 | -1.598 | 0.402 | -20.12% | 0.113 | 0.026 |
|  | σ^2_(u_0) (10) | 25.563 | 15.563 | 155.63% | 1.538 | 3.119 | 23.214 | 13.214 | 132.14% | 1.030 | 0.900 | 23.361 | 13.361 | 133.61% | 0.732 | 1.772 |
|  | σ_u_0 u_1 (0) | -1.842 | -1.842 | NA | 0.751 | 0.713 | -1.606 | -1.606 | NA | 0.522 | 0.276 | -1.457 | -1.457 | NA | 0.371 | 0.095 |
|  | σ^2_(u_1 ) (10) | 10.200 | 0.200 | 2.00% | 0.597 | 0.182 | 10.022 | 0.022 | 0.22% | 0.426 | 0.433 | 9.767 | -0.233 | -2.33% | 0.296 | 0.313 |
|  | σ_(v_L ) (2.2361) | 2.359 | -1.884 | -44.41% | 0.557 | 0.554 | 2.308 | -1.934 | -45.60% | 0.338 | 0.132 | 2.126 | -2.117 | -49.90% | 0.225 | 0.242 |
|  | σ_(v_S ) (0.6325) | 0.279 | -0.353 | -55.87% | 0.075 | 0.078 | 0.331 | -0.301 | -47.62% | 0.054 | 0.051 | 0.232 | -0.400 | -63.27% | 0.031 | 0.044 |
|  | σ_ε (5.7446) | 5.699 | -0.045 | -0.79% | 0.044 | 0.023 | 5.722 | -0.022 | -0.39% | 0.032 | 0.024 | 5.747 | 0.003 | 0.05% | 0.023 | 0.003 |
| K=3 | α (-0.2) | -0.147 | 0.053 | -26.34% | 0.007 | 0.008 | -0.151 | 0.049 | -24.31% | 0.005 | 0.003 | -0.150 | 0.050 | -24.79% | 0.004 | 0.004 |
|  | β_000 (16) | 15.740 | -0.260 | -1.63% | 0.616 | 0.449 | 15.530 | -0.470 | -2.94% | 0.431 | 0.575 | 15.527 | -0.473 | -2.96% | 0.282 | 0.168 |
|  | β_100 (-0.8) | -0.022 | 0.778 | -97.28% | 0.153 | 0.136 | 0.057 | 0.857 | -107.07% | 0.112 | 0.200 | -0.070 | 0.730 | -91.19% | 0.081 | 0.085 |
|  | β_001 (1.6) | 1.189 | -0.411 | -25.69% | 0.377 | 1.138 | 1.320 | -0.280 | -17.48% | 0.294 | 0.525 | 1.727 | 0.127 | 7.96% | 0.199 | 0.315 |
|  | β_101 (-2) | -1.820 | 0.180 | -8.99% | 0.216 | 0.023 | -1.867 | 0.133 | -6.64% | 0.152 | 0.259 | -1.637 | 0.363 | -18.14% | 0.107 | 0.039 |
|  | σ^2_(u_0) (10) | 20.604 | 10.604 | 106.04% | 1.321 | 1.657 | 18.731 | 8.731 | 87.31% | 0.897 | 0.951 | 19.281 | 9.281 | 92.81% | 0.645 | 1.230 |
|  | σ_u_0 u_1 (0) | -0.742 | -0.742 | NA | 0.658 | 0.700 | -0.374 | -0.374 | NA | 0.457 | 0.240 | -0.368 | -0.368 | NA | 0.326 | 0.075 |
|  | σ^2_(u_1 ) (10) | 8.845 | -1.155 | -11.55% | 0.519 | 0.102 | 8.495 | -1.505 | -15.05% | 0.361 | 0.349 | 8.271 | -1.729 | -17.29% | 0.250 | 0.261 |
|  | σ_(v_L ) (2.2361) | 3.013 | -1.230 | -28.98% | 0.452 | 0.492 | 2.981 | -1.262 | -29.74% | 0.306 | 0.070 | 2.682 | -1.561 | -36.79% | 0.196 | 0.205 |
|  | σ_(v_S ) (0.6325) | 0.270 | -0.362 | -57.29% | 0.056 | 0.018 | 0.304 | -0.329 | -51.96% | 0.040 | 0.055 | 0.261 | -0.371 | -58.73% | 0.027 | 0.023 |
|  | σ_ε (5.7446) | 5.726 | -0.019 | -0.33% | 0.045 | 0.023 | 5.755 | 0.010 | 0.18% | 0.032 | 0.024 | 5.777 | 0.032 | 0.56% | 0.023 | 0.003 |

Table A 1‑4 Results of the proposed MJM censoring rate 20%, ICC at the level 3 in the longitudinal submodel = 0.3, group-level variance in the survival submodel = 1

|  | L3 ICC in longitudinal outcome = 0.3; variance at L2 in survival = 0.4 | | | | | | | | | | | | | | | |
| --- | --- | --- | --- | --- | --- | --- | --- | --- | --- | --- | --- | --- | --- | --- | --- | --- |
|  | Parameter | Mean | Bias | RB | MESE | ESE | Mean | Bias | RB | MESE | ESE | Mean | Bias | RB | MESE | ESE |
|  |  | n = 50 | | | | | n = 100 | | | | | n = 200 | | | | |
| K=11 | α (-0.2) | -0.147 | 0.053 | -26.52% | 0.007 | 0.002 | -0.146 | 0.054 | -27.19% | 0.005 | 0.009 | -0.144 | 0.056 | -27.87% | 0.003 | 0.003 |
|  | β_000 (16) | 16.208 | 0.208 | 1.30% | 0.795 | 0.475 | 15.554 | -0.446 | -2.79% | 0.516 | 0.469 | 15.819 | -0.181 | -1.13% | 0.350 | 0.389 |
|  | β_100 (-0.8) | -0.600 | 0.200 | -24.95% | 0.167 | 0.139 | -0.534 | 0.266 | -33.26% | 0.124 | 0.287 | -0.653 | 0.147 | -18.34% | 0.090 | 0.041 |
|  | β_001 (1.6) | 1.178 | -0.422 | -26.39% | 0.364 | 1.253 | 1.455 | -0.145 | -9.06% | 0.272 | 0.637 | 1.769 | 0.169 | 10.53% | 0.177 | 0.614 |
|  | β_101 (-2) | -1.778 | 0.222 | -11.11% | 0.230 | 0.126 | -1.771 | 0.229 | -11.47% | 0.164 | 0.227 | -1.669 | 0.331 | -16.57% | 0.117 | 0.019 |
|  | σ^2_(u_0) (10) | 27.552 | 17.552 | 175.52% | 1.630 | 3.269 | 25.988 | 15.988 | 159.88% | 1.118 | 0.767 | 24.896 | 14.896 | 148.96% | 0.767 | 1.084 |
|  | σ_u_0 u_1 (0) | -1.249 | -1.249 | NA | 0.790 | 0.610 | -1.654 | -1.654 | NA | 0.555 | 0.522 | -1.188 | -1.188 | NA | 0.389 | 0.493 |
|  | σ^2_(u_1 ) (10) | 10.451 | 0.451 | 4.51% | 0.616 | 0.157 | 10.388 | 0.388 | 3.88% | 0.443 | 0.432 | 10.467 | 0.467 | 4.67% | 0.317 | 0.187 |
|  | σ_(v_L ) (2.2361) | 2.081 | -2.162 | -50.95% | 0.687 | 1.074 | 2.117 | -2.126 | -50.10% | 0.399 | 0.326 | 1.879 | -2.363 | -55.70% | 0.258 | 0.122 |
|  | σ_(v_S ) (0.6325) | 0.311 | -0.689 | -68.92% | 0.106 | 0.092 | 0.352 | -0.648 | -64.85% | 0.069 | 0.060 | 0.284 | -0.716 | -71.56% | 0.040 | 0.028 |
|  | σ_ε (5.7446) | 5.709 | -0.036 | -0.62% | 0.045 | 0.040 | 5.726 | -0.018 | -0.32% | 0.032 | 0.017 | 5.743 | -0.001 | -0.02% | 0.023 | 0.009 |
| K=7 | α (-0.2) | -0.152 | 0.048 | -24.02% | 0.007 | 0.006 | -0.154 | 0.046 | -23.01% | 0.005 | 0.011 | -0.149 | 0.051 | -25.73% | 0.003 | 0.001 |
|  | β_000 (16) | 16.270 | 0.270 | 1.69% | 0.748 | 0.244 | 15.707 | -0.293 | -1.83% | 0.502 | 0.338 | 15.824 | -0.176 | -1.10% | 0.307 | 0.493 |
|  | β_100 (-0.8) | -0.513 | 0.287 | -35.93% | 0.166 | 0.144 | -0.410 | 0.390 | -48.79% | 0.121 | 0.285 | -0.519 | 0.281 | -35.17% | 0.089 | 0.025 |
|  | β_001 (1.6) | 1.016 | -0.584 | -36.52% | 0.362 | 1.142 | 1.376 | -0.224 | -13.99% | 0.267 | 0.769 | 1.777 | 0.177 | 11.04% | 0.182 | 0.746 |
|  | β_101 (-2) | -1.746 | 0.254 | -12.68% | 0.231 | 0.109 | -1.805 | 0.195 | -9.73% | 0.163 | 0.240 | -1.695 | 0.305 | -15.24% | 0.116 | 0.007 |
|  | σ^2_(u_0) (10) | 25.132 | 15.132 | 151.32% | 1.534 | 3.401 | 22.973 | 12.973 | 129.73% | 1.028 | 0.561 | 23.687 | 13.687 | 136.87% | 0.744 | 0.402 |
|  | σ_u_0 u_1 (0) | -1.466 | -1.466 | NA | 0.766 | 0.486 | -1.376 | -1.376 | NA | 0.524 | 0.461 | -1.289 | -1.289 | NA | 0.378 | 0.537 |
|  | σ^2_(u_1 ) (10) | 10.340 | 0.340 | 3.40% | 0.611 | 0.209 | 10.138 | 0.138 | 1.38% | 0.430 | 0.472 | 10.275 | 0.275 | 2.75% | 0.311 | 0.138 |
|  | σ_(v_L ) (2.2361) | 2.530 | -1.713 | -40.37% | 0.590 | 0.656 | 2.564 | -1.679 | -39.58% | 0.368 | 0.273 | 2.077 | -2.166 | -51.04% | 0.224 | 0.371 |
|  | σ_(v_S ) (0.6325) | 0.340 | -0.660 | -66.00% | 0.087 | 0.101 | 0.453 | -0.547 | -54.66% | 0.068 | 0.040 | 0.323 | -0.677 | -67.70% | 0.038 | 0.053 |
|  | σ_ε (5.7446) | 5.714 | -0.031 | -0.54% | 0.045 | 0.038 | 5.733 | -0.012 | -0.21% | 0.032 | 0.017 | 5.749 | 0.004 | 0.007% | 0.023 | 0.011 |
| K=3 | α (-0.2) | -0.142 | 0.058 | -29.23% | 0.007 | 0.003 | -0.152 | 0.048 | -23.93% | 0.005 | 0.009 | -0.145 | 0.055 | -27.51% | 0.004 | 0.001 |
|  | β_000 (16) | 15.806 | -0.194 | -1.21% | 0.609 | 0.241 | 15.598 | -0.402 | -2.51% | 0.440 | 0.438 | 15.466 | -0.534 | -3.33% | 0.298 | 0.326 |
|  | β_100 (-0.8) | -0.126 | 0.674 | -84.22% | 0.157 | 0.182 | -0.048 | 0.752 | -93.97% | 0.115 | 0.296 | -0.150 | 0.650 | -81.22% | 0.084 | 0.033 |
|  | β_001 (1.6) | 1.151 | -0.449 | -28.07% | 0.376 | 0.754 | 1.105 | -0.495 | -30.94% | 0.299 | 0.591 | 1.925 | 0.325 | 20.34% | 0.199 | 0.374 |
|  | β_101 (-2) | -1.824 | 0.176 | -8.78% | 0.221 | 0.109 | -1.819 | 0.181 | -9.05% | 0.155 | 0.288 | -1.703 | 0.297 | -14.85% | 0.110 | 0.020 |
|  | σ^2_(u_0) (10) | 20.865 | 10.865 | 108.65% | 1.354 | 2.383 | 19.453 | 9.453 | 94.53% | 0.917 | 0.933 | 18.906 | 8.906 | 89.06% | 0.639 | 1.204 |
|  | σ_u_0 u_1 (0) | -0.332 | -0.332 | NA | 0.680 | 0.472 | -0.409 | -0.409 | NA | 0.470 | 0.644 | -0.006 | -0.006 | NA | 0.330 | 0.397 |
|  | σ^2_(u_1 ) (10) | 9.015 | -0.985 | -9.85% | 0.531 | 0.168 | 8.844 | -1.156 | -11.56% | 0.375 | 0.372 | 8.808 | -1.192 | -11.92% | 0.265 | 0.096 |
|  | σ_(v_L ) (2.2361) | 2.970 | -1.273 | -30.00% | 0.448 | 0.534 | 3.024 | -1.218 | -28.72% | 0.310 | 0.110 | 2.868 | -1.375 | -32.41% | 0.210 | 0.118 |
|  | σ_(v_S ) (0.6325) | 0.393 | -0.607 | -60.67% | 0.068 | 0.053 | 0.477 | -0.523 | -52.35% | 0.054 | 0.056 | 0.424 | -0.576 | -57.64% | 0.035 | 0.021 |
|  | σ_ε (5.7446) | 5.741 | -0.004 | -0.06% | 0.045 | 0.038 | 5.757 | 0.012 | 0.21% | 0.032 | 0.018 | 5.776 | 0.031 | 0.54% | 0.023 | 0.014 |

Table A 1‑5 Results of the proposed MJM censoring rate 60%, ICC at the level 3 in the longitudinal submodel = 0.1, group-level variance in the survival submodel = 0.4

|  | L3 ICC in longitudinal outcome = 0.1; variance at L2 in survival = 0.4 | | | | | | | | | | | | | | | |
| --- | --- | --- | --- | --- | --- | --- | --- | --- | --- | --- | --- | --- | --- | --- | --- | --- |
|  | Parameter | Mean | Bias | RB | MESE | ESE | Mean | Bias | RB | MESE | ESE | Mean | Bias | RB | MESE | ESE |
|  |  | n = 50 | | | | | n = 100 | | | | | n = 200 | | | | |
| K=11 | α (-0.2) | -0.142 | 0.058 | -28.93% | 0.007 | 0.005 | -0.142 | 0.058 | -29.08% | 0.006 | 0.004 | -0.138 | 0.062 | -31.06% | 0.004 | 0.006 |
|  | β_000 (16) | 16.019 | 0.019 | 0.12% | 0.601 | 0.223 | 15.788 | -0.212 | -1.33% | 0.326 | 0.239 | 15.809 | -0.191 | -1.19% | 0.253 | 0.202 |
|  | β_100 (-0.8) | -0.923 | -0.123 | 15.37% | 0.130 | 0.020 | -0.940 | -0.140 | 17.45% | 0.093 | 0.109 | -1.024 | -0.224 | 28.04% | 0.067 | 0.119 |
|  | β_001 (1.6) | 1.482 | -0.118 | -7.37% | 0.275 | 0.435 | 1.612 | 0.012 | 0.75% | 0.199 | 0.170 | 1.773 | 0.173 | 10.81% | 0.137 | 0.387 |
|  | β_101 (-2) | -1.677 | 0.323 | -16.13% | 0.183 | 0.121 | -1.654 | 0.346 | -17.29% | 0.128 | 0.075 | -1.581 | 0.419 | -20.94% | 0.089 | 0.062 |
|  | σ^2_(u_0) (10) | 15.131 | 5.131 | 51.31% | 0.956 | 1.734 | 14.683 | 4.683 | 46.83% | 0.645 | 0.468 | 14.945 | 4.945 | 49.45% | 0.463 | 0.777 |
|  | σ_u_0 u_1 (0) | -0.570 | -0.570 | NA | 0.476 | 0.154 | 0.003 | 0.003 | NA | 0.325 | 0.223 | 0.064 | 0.064 | NA | 0.231 | 0.137 |
|  | σ^2_(u_1 ) (10) | 10.761 | 0.761 | 7.61% | 0.484 | 0.288 | 10.690 | 0.690 | 6.90% | 0.339 | 0.382 | 10.590 | 0.590 | 5.90% | 0.240 | 0.259 |
|  | σ_(v_L ) (2.2361) | 0.963 | -1.273 | -56.95% | 0.603 | 0.316 | 0.813 | -1.423 | -63.64% | 0.273 | 0.113 | 0.664 | -1.572 | -70.31% | 0.211 | 0.099 |
|  | σ_(v_S ) (0.6325) | 0.210 | -0.422 | -66.79% | 0.109 | 0.055 | 0.297 | -0.336 | -53.10% | 0.092 | 0.068 | 0.216 | -0.417 | -65.88% | 0.052 | 0.017 |
|  | σ_ε (5.7446) | 5.747 | 0.003 | 0.01% | 0.033 | 0.047 | 5.738 | -0.007 | -0.12% | 0.022 | 0.018 | 5.735 | -0.010 | -0.17% | 0.016 | 0.007 |
| K=7 | α (-0.2) | -0.145 | 0.055 | -27.42% | 0.007 | 0.006 | -0.144 | 0.056 | -27.89% | 0.006 | 0.004 | -0.139 | 0.061 | -30.73% | 0.004 | 0.005 |
|  | β_000 (16) | 16.055 | 0.055 | 0.34% | 0.426 | 0.238 | 15.791 | -0.209 | -1.31% | 0.267 | 0.240 | 15.831 | -0.169 | -1.06% | 0.204 | 0.228 |
|  | β_100 (-0.8) | -0.878 | -0.078 | 9.74% | 0.131 | 0.031 | -0.893 | -0.093 | 11.59% | 0.092 | 0.114 | -0.986 | -0.186 | 23.22% | 0.066 | 0.114 |
|  | β_001 (1.6) | 1.363 | -0.237 | -14.80% | 0.279 | 0.371 | 1.595 | -0.005 | -0.34% | 0.203 | 0.174 | 1.734 | 0.134 | 8.40% | 0.140 | 0.397 |
|  | β_101 (-2) | -1.719 | 0.281 | -14.06% | 0.184 | 0.102 | -1.693 | 0.307 | -15.37% | 0.127 | 0.075 | -1.613 | 0.387 | -19.36% | 0.089 | 0.060 |
|  | σ^2_(u_0) (10) | 14.830 | 4.830 | 48.30% | 0.945 | 1.515 | 14.597 | 4.597 | 45.97% | 0.644 | 0.683 | 14.735 | 4.735 | 47.35% | 0.456 | 0.705 |
|  | σ_u_0 u_1 (0) | -0.483 | -0.483 | NA | 0.471 | 0.136 | 0.063 | 0.063 | NA | 0.322 | 0.215 | 0.119 | 0.119 | NA | 0.231 | 0.098 |
|  | σ^2_(u_1 ) (10) | 10.662 | 0.662 | 6.62% | 0.480 | 0.274 | 10.546 | 0.546 | 5.46% | 0.333 | 0.372 | 10.496 | 0.496 | 4.96% | 0.238 | 0.210 |
|  | σ_(v_L ) (2.2361) | 1.027 | -1.209 | -54.08% | 0.372 | 0.421 | 0.849 | -1.387 | -62.03% | 0.208 | 0.121 | 0.806 | -1.430 | -63.95% | 0.156 | 0.062 |
|  | σ_(v_S ) (0.6325) | 0.239 | -0.393 | -62.16% | 0.095 | 0.018 | 0.383 | -0.249 | -39.40% | 0.093 | 0.087 | 0.279 | -0.354 | -55.92% | 0.052 | 0.035 |
|  | σ_ε (5.7446) | 5.748 | 0.003 | 0.01% | 0.033 | 0.047 | 5.738 | -0.007 | -0.12% | 0.022 | 0.018 | 5.735 | -0.010 | -0.17% | 0.016 | 0.007 |
| K=3 | α (-0.2) | -0.134 | 0.066 | -32.84% | 0.007 | 0.001 | -0.132 | 0.068 | -34.20% | 0.005 | 0.001 | -0.123 | 0.077 | -38.44% | 0.003 | 0.003 |
|  | β_000 (16) | 15.863 | -0.137 | -0.86% | 0.401 | 0.208 | 15.783 | -0.217 | -1.36% | 0.278 | 0.199 | 15.702 | -0.298 | -1.86% | 0.175 | 0.151 |
|  | β_100 (-0.8) | -0.687 | 0.113 | -14.18% | 0.130 | 0.012 | -0.711 | 0.089 | -11.16% | 0.091 | 0.136 | -0.759 | 0.041 | -5.06% | 0.066 | 0.127 |
|  | β_001 (1.6) | 1.369 | -0.231 | -14.42% | 0.298 | 0.358 | 1.445 | -0.155 | -9.71% | 0.221 | 0.177 | 1.831 | 0.231 | 14.41% | 0.152 | 0.270 |
|  | β_101 (-2) | -1.787 | 0.213 | -10.65% | 0.184 | 0.127 | -1.752 | 0.248 | -12.42% | 0.127 | 0.114 | -1.699 | 0.301 | -15.07% | 0.089 | 0.071 |
|  | σ^2_(u_0) (10) | 14.057 | 4.057 | 40.57% | 0.913 | 1.543 | 13.531 | 3.531 | 35.31% | 0.614 | 0.720 | 14.132 | 4.132 | 41.32% | 0.446 | 0.618 |
|  | σ_u_0 u_1 (0) | -0.302 | -0.302 | NA | 0.461 | 0.176 | 0.395 | 0.395 | NA | 0.311 | 0.223 | 0.448 | 0.448 | NA | 0.222 | 0.093 |
|  | σ^2_(u_1 ) (10) | 10.178 | 0.178 | 1.78% | 0.466 | 0.267 | 10.017 | 0.017 | 0.17% | 0.322 | 0.378 | 9.839 | -0.161 | -1.61% | 0.228 | 0.311 |
|  | σ_(v_L ) (2.2361) | 1.508 | -0.728 | -32.57% | 0.303 | 0.253 | 1.425 | -0.811 | -36.26% | 0.202 | 0.036 | 1.196 | -1.040 | -46.52% | 0.125 | 0.072 |
|  | σ_(v_S ) (0.6325) | 0.204 | -0.429 | -67.76% | 0.067 | 0.017 | 0.320 | -0.312 | -49.36% | 0.062 | 0.097 | 0.253 | -0.380 | -60.02% | 0.040 | 0.047 |
|  | σ_ε (5.7446) | 5.751 | 0.007 | 0.12% | 0.033 | 0.047 | 5.739 | -0.005 | -0.09% | 0.022 | 0.018 | 5.737 | -0.008 | -0.13% | 0.016 | 0.007 |

Table A 1‑6 Results of the proposed MJM censoring rate 60%, ICC at the level 3 in the longitudinal submodel = 0.1, group-level variance in the survival submodel = 1\

|  | L3 ICC in longitudinal outcome = 0.1; variance at L2 in survival = 1 | | | | | | | | | | | | | | | |
| --- | --- | --- | --- | --- | --- | --- | --- | --- | --- | --- | --- | --- | --- | --- | --- | --- |
|  | Parameter | Mean | Bias | RB | MESE | ESE | Mean | Bias | RB | MESE | ESE | Mean | Bias | RB | MESE | ESE |
|  |  | n = 50 | | | | | n = 100 | | | | | n = 200 | | | | |
| K=11 | α (-0.2) | -0.133 | 0.067 | -33.74% | 0.008 | 0.016 | -0.136 | 0.064 | -31.96% | 0.005 | 0.001 | -0.129 | 0.071 | -35.39% | 0.004 | 0.004 |
|  | β_000 (16) | 15.938 | -0.062 | -0.39% | 0.452 | 0.239 | 15.889 | -0.111 | -0.69% | 0.295 | 0.251 | 15.814 | -0.186 | -1.17% | 0.237 | 0.273 |
|  | β_100 (-0.8) | -0.934 | -0.134 | 16.73% | 0.130 | 0.052 | -0.981 | -0.181 | 22.67% | 0.093 | 0.110 | -0.998 | -0.198 | 24.74% | 0.066 | 0.118 |
|  | β_001 (1.6) | 1.559 | -0.041 | -2.56% | 0.275 | 0.530 | 1.533 | -0.067 | -4.21% | 0.198 | 0.217 | 1.764 | 0.164 | 10.27% | 0.136 | 0.456 |
|  | β_101 (-2) | -1.711 | 0.289 | -14.45% | 0.182 | 0.153 | -1.639 | 0.361 | -18.07% | 0.128 | 0.069 | -1.611 | 0.389 | -19.45% | 0.089 | 0.078 |
|  | σ^2_(u_0) (10) | 15.531 | 5.531 | 55.31% | 0.941 | 1.785 | 14.499 | 4.499 | 44.99% | 0.636 | 0.334 | 14.715 | 4.715 | 47.15% | 0.453 | 0.868 |
|  | σ_u_0 u_1 (0) | -0.136 | -0.136 | NA | 0.471 | 0.402 | -0.061 | -0.061 | NA | 0.326 | 0.098 | 0.093 | 0.093 | NA | 0.231 | 0.107 |
|  | σ^2_(u_1 ) (10) | 10.717 | 0.717 | 7.17% | 0.486 | 0.522 | 10.753 | 0.753 | 7.53% | 0.342 | 0.229 | 10.503 | 0.503 | 5.03% | 0.241 | 0.232 |
|  | σ_(v_L ) (2.2361) | 0.762 | -1.474 | -65.93% | 0.425 | 0.444 | 0.753 | -1.483 | -66.31% | 0.244 | 0.106 | 0.717 | -1.519 | -67.94% | 0.188 | 0.063 |
|  | σ_(v_S ) (0.6325) | 0.306 | -0.694 | -69.39% | 0.155 | 0.111 | 0.338 | -0.662 | -66.15% | 0.099 | 0.088 | 0.258 | -0.742 | -74.22% | 0.056 | 0.024 |
|  | σ_ε (5.7446) | 5.736 | -0.009 | -0.15% | 0.032 | 0.034 | 5.737 | -0.008 | -0.13% | 0.022 | 0.021 | 5.738 | -0.006 | -0.11% | 0.016 | 0.006 |
| K=7 | α (-0.2) | -0.136 | 0.064 | -31.86% | 0.008 | 0.015 | -0.137 | 0.063 | -31.64% | 0.005 | 0.001 | -0.131 | 0.069 | -34.53% | 0.004 | 0.005 |
|  | β_000 (16) | 15.929 | -0.071 | -0.44% | 0.403 | 0.107 | 15.955 | -0.045 | -0.28% | 0.294 | 0.261 | 15.834 | -0.166 | -1.04% | 0.196 | 0.322 |
|  | β_100 (-0.8) | -0.913 | -0.113 | 14.10% | 0.129 | 0.038 | -0.940 | -0.140 | 17.54% | 0.093 | 0.121 | -0.974 | -0.174 | 21.80% | 0.066 | 0.114 |
|  | β_001 (1.6) | 1.525 | -0.075 | -4.69% | 0.273 | 0.412 | 1.429 | -0.171 | -10.72% | 0.201 | 0.230 | 1.734 | 0.134 | 8.39% | 0.139 | 0.501 |
|  | β_101 (-2) | -1.729 | 0.271 | -13.57% | 0.182 | 0.167 | -1.664 | 0.336 | -16.80% | 0.127 | 0.073 | -1.629 | 0.371 | -18.53% | 0.089 | 0.076 |
|  | σ^2_(u_0) (10) | 15.378 | 5.378 | 53.78% | 0.936 | 1.879 | 14.210 | 4.210 | 42.10% | 0.629 | 0.108 | 14.484 | 4.484 | 44.84% | 0.451 | 0.778 |
|  | σ_u_0 u_1 (0) | -0.098 | -0.098 | NA | 0.469 | 0.332 | 0.051 | 0.051 | NA | 0.322 | 0.106 | 0.141 | 0.141 | NA | 0.228 | 0.107 |
|  | σ^2_(u_1 ) (10) | 10.662 | 0.662 | 6.62% | 0.483 | 0.490 | 10.619 | 0.619 | 6.19% | 0.338 | 0.243 | 10.443 | 0.443 | 4.43% | 0.238 | 0.186 |
|  | σ_(v_L ) (2.2361) | 0.910 | -1.326 | -59.31% | 0.362 | 0.315 | 0.951 | -1.285 | -57.48% | 0.234 | 0.113 | 0.873 | -1.363 | -60.96% | 0.147 | 0.091 |
|  | σ_(v_S ) (0.6325) | 0.344 | -0.656 | -65.62% | 0.134 | 0.078 | 0.376 | -0.624 | -62.42% | 0.090 | 0.096 | 0.294 | -0.706 | -70.58% | 0.052 | 0.026 |
|  | σ_ε (5.7446) | 5.736 | -0.008 | -0.15% | 0.032 | 0.034 | 5.737 | -0.007 | -0.13% | 0.022 | 0.021 | 5.738 | -0.006 | -0.11% | 0.016 | 0.006 |
| K=3 | α (-0.2) | -0.124 | 0.076 | -37.95% | 0.007 | 0.007 | -0.129 | 0.071 | -35.45% | 0.005 | 0.001 | -0.122 | 0.078 | -38.78% | 0.003 | 0.003 |
|  | β_000 (16) | 15.929 | -0.071 | -0.45% | 0.396 | 0.172 | 15.900 | -0.100 | -0.63% | 0.276 | 0.212 | 15.715 | -0.285 | -1.78% | 0.179 | 0.191 |
|  | β_100 (-0.8) | -0.688 | 0.112 | -14.03% | 0.127 | 0.017 | -0.738 | 0.062 | -7.75% | 0.092 | 0.136 | -0.751 | 0.049 | -6.11% | 0.066 | 0.107 |
|  | β_001 (1.6) | 1.377 | -0.223 | -13.94% | 0.297 | 0.306 | 1.342 | -0.258 | -16.11% | 0.218 | 0.198 | 1.789 | 0.189 | 11.80% | 0.152 | 0.289 |
|  | β_101 (-2) | -1.806 | 0.194 | -9.69% | 0.179 | 0.155 | -1.746 | 0.254 | -12.69% | 0.127 | 0.118 | -1.722 | 0.278 | -13.91% | 0.089 | 0.067 |
|  | σ^2_(u_0) (10) | 14.356 | 4.356 | 43.56% | 0.899 | 1.677 | 13.325 | 3.325 | 33.25% | 0.606 | 0.098 | 13.906 | 3.906 | 39.06% | 0.440 | 0.731 |
|  | σ_u_0 u_1 (0) | 0.220 | 0.220 | NA | 0.448 | 0.232 | 0.398 | 0.398 | NA | 0.308 | 0.134 | 0.427 | 0.427 | NA | 0.220 | 0.041 |
|  | σ^2_(u_1 ) (10) | 9.926 | -0.074 | -0.74% | 0.455 | 0.478 | 9.980 | -0.020 | -0.20% | 0.321 | 0.262 | 9.772 | -0.228 | -2.28% | 0.225 | 0.263 |
|  | σ_(v_L ) (2.2361) | 1.441 | -0.795 | -35.57% | 0.304 | 0.235 | 1.419 | -0.817 | -36.52% | 0.201 | 0.070 | 1.234 | -1.002 | -44.83% | 0.127 | 0.077 |
|  | σ_(v_S ) (0.6325) | 0.286 | -0.714 | -71.35% | 0.084 | 0.049 | 0.399 | -0.601 | -60.14% | 0.067 | 0.091 | 0.324 | -0.676 | -67.63% | 0.042 | 0.052 |
|  | σ_ε (5.7446) | 5.739 | -0.005 | -0.09% | 0.032 | 0.033 | 5.740 | -0.005 | -0.08% | 0.022 | 0.021 | 5.741 | -0.004 | -0.07% | 0.016 | 0.006 |

Table A 1‑7 Results of the proposed MJM censoring rate 60%, ICC at the level 3 in the longitudinal submodel = 0.3, group-level variance in the survival submodel = 0.4

|  | L3 ICC in longitudinal outcome = 0.3; variance at L2 in survival = 0.4 | | | | | | | | | | | | | | | |
| --- | --- | --- | --- | --- | --- | --- | --- | --- | --- | --- | --- | --- | --- | --- | --- | --- |
|  | Parameter | Mean | Bias | RB | MESE | ESE | Mean | Bias | RB | MESE | ESE | Mean | Bias | RB | MESE | ESE |
|  |  | n = 50 | | | | | n = 100 | | | | | n = 200 | | | | |
| K=11 | α (-0.2) | -0.147 | 0.053 | -26.53% | 0.008 | 0.017 | -0.144 | 0.056 | -27.79% | 0.006 | 0.004 | -0.141 | 0.059 | -29.33% | 0.004 | 0.005 |
|  | β_000 (16) | 15.915 | -0.085 | -0.53% | 0.752 | 0.395 | 15.569 | -0.431 | -2.70% | 0.489 | 0.371 | 15.640 | -0.360 | -2.25% | 0.331 | 0.380 |
|  | β_100 (-0.8) | -0.873 | -0.073 | 9.14% | 0.127 | 0.067 | -0.949 | -0.149 | 18.67% | 0.093 | 0.088 | -0.998 | -0.198 | 24.77% | 0.066 | 0.116 |
|  | β_001 (1.6) | 1.367 | -0.233 | -14.59% | 0.337 | 0.825 | 1.740 | 0.140 | 8.75% | 0.243 | 0.338 | 1.978 | 0.378 | 23.62% | 0.166 | 0.641 |
|  | β_101 (-2) | -1.734 | 0.266 | -13.32% | 0.180 | 0.127 | -1.652 | 0.348 | -17.38% | 0.128 | 0.030 | -1.606 | 0.394 | -19.68% | 0.090 | 0.083 |
|  | σ^2_(u_0) (10) | 28.743 | 18.743 | 187.43% | 1.458 | 4.011 | 25.561 | 15.561 | 155.61% | 0.944 | 0.913 | 26.485 | 16.485 | 164.85% | 0.686 | 1.406 |
|  | σ_u_0 u_1 (0) | -0.242 | -0.242 | NA | 0.570 | 0.166 | -0.003 | -0.003 | NA | 0.391 | 0.343 | -0.059 | -0.059 | NA | 0.278 | 0.410 |
|  | σ^2_(u_1 ) (10) | 10.492 | 0.492 | 4.92% | 0.471 | 0.397 | 10.745 | 0.745 | 7.45% | 0.341 | 0.376 | 10.517 | 0.517 | 5.17% | 0.238 | 0.226 |
|  | σ_(v_L ) (2.2361) | 1.469 | -2.773 | -65.37% | 0.721 | 0.741 | 1.411 | -2.832 | -66.74% | 0.398 | 0.146 | 1.345 | -2.897 | -68.29% | 0.253 | 0.134 |
|  | σ_(v_S ) (0.6325) | 0.253 | -0.379 | -59.94% | 0.141 | 0.039 | 0.297 | -0.335 | -53.02% | 0.092 | 0.055 | 0.234 | -0.398 | -62.96% | 0.055 | 0.020 |
|  | σ_ε (5.7446) | 5.741 | -0.004 | -0.06% | 0.032 | 0.042 | 5.737 | -0.007 | -0.13% | 0.022 | 0.017 | 5.738 | -0.006 | -0.11% | 0.016 | 0.004 |
| K=7 | α (-0.2) | -0.146 | 0.054 | -27.10% | 0.008 | 0.015 | -0.146 | 0.054 | -27.18% | 0.006 | 0.004 | -0.146 | 0.054 | -27.21% | 0.006 | 0.004 |
|  | β_000 (16) | 16.025 | 0.025 | 0.16% | 0.696 | 0.104 | 15.632 | -0.368 | -2.30% | 0.450 | 0.430 | 15.633 | -0.367 | -2.30% | 0.446 | 0.429 |
|  | β_100 (-0.8) | -0.840 | -0.040 | 4.98% | 0.127 | 0.071 | -0.908 | -0.108 | 13.56% | 0.093 | 0.099 | -0.911 | -0.111 | 13.84% | 0.091 | 0.100 |
|  | β_001 (1.6) | 1.214 | -0.386 | -24.13% | 0.340 | 0.580 | 1.604 | 0.004 | 0.22% | 0.246 | 0.410 | 1.611 | 0.011 | 0.70% | 0.244 | 0.423 |
|  | β_101 (-2) | -1.757 | 0.243 | -12.14% | 0.179 | 0.128 | -1.678 | 0.322 | -16.10% | 0.127 | 0.042 | -1.675 | 0.325 | -16.24% | 0.126 | 0.045 |
|  | σ^2_(u_0) (10) | 27.946 | 17.946 | 179.46% | 1.424 | 4.107 | 24.657 | 14.657 | 146.57% | 0.921 | 1.159 | 24.673 | 14.673 | 146.73% | 0.912 | 1.164 |
|  | σ_u_0 u_1 (0) | -0.187 | -0.187 | NA | 0.562 | 0.141 | 0.088 | 0.088 | NA | 0.385 | 0.365 | 0.086 | 0.086 | NA | 0.380 | 0.366 |
|  | σ^2_(u_1 ) (10) | 10.410 | 0.410 | 4.10% | 0.469 | 0.387 | 10.612 | 0.612 | 6.12% | 0.338 | 0.399 | 10.605 | 0.605 | 6.05% | 0.335 | 0.391 |
|  | σ_(v_L ) (2.2361) | 1.756 | -2.487 | -58.61% | 0.601 | 0.688 | 1.632 | -2.611 | -61.54% | 0.344 | 0.314 | 1.634 | -2.609 | -61.49% | 0.341 | 0.312 |
|  | σ_(v_S ) (0.6325) | 0.257 | -0.375 | -59.29% | 0.111 | 0.064 | 0.384 | -0.249 | -39.32% | 0.091 | 0.069 | 0.381 | -0.251 | -39.69% | 0.090 | 0.070 |
|  | σ_ε (5.7446) | 5.741 | -0.004 | -0.06% | 0.032 | 0.041 | 5.738 | -0.007 | -0.12% | 0.022 | 0.017 | 5.738 | -0.007 | -0.12% | 0.022 | 0.017 |
| K=3 | α (-0.2) | -0.130 | 0.070 | -34.94% | 0.007 | 0.011 | -0.130 | 0.070 | -34.96% | 0.005 | 0.004 | -0.122 | 0.078 | -38.96% | 0.003 | 0.003 |
|  | β_000 (16) | 16.083 | 0.083 | 0.52% | 0.716 | 0.129 | 15.559 | -0.441 | -2.76% | 0.445 | 0.395 | 15.523 | -0.477 | -2.98% | 0.303 | 0.289 |
|  | β_100 (-0.8) | -0.633 | 0.167 | -20.88% | 0.126 | 0.067 | -0.707 | 0.093 | -11.62% | 0.092 | 0.122 | -0.727 | 0.073 | -9.06% | 0.066 | 0.125 |
|  | β_001 (1.6) | 0.878 | -0.722 | -45.14% | 0.354 | 0.268 | 1.520 | -0.080 | -5.00% | 0.262 | 0.269 | 2.002 | 0.402 | 25.14% | 0.183 | 0.446 |
|  | β_101 (-2) | -1.836 | 0.164 | -8.19% | 0.178 | 0.112 | -1.752 | 0.248 | -12.39% | 0.127 | 0.071 | -1.724 | 0.276 | -13.81% | 0.089 | 0.086 |
|  | σ^2_(u_0) (10) | 23.534 | 13.534 | 135.34% | 1.260 | 2.673 | 21.696 | 11.696 | 116.96% | 0.843 | 0.852 | 23.105 | 13.105 | 131.05% | 0.621 | 0.922 |
|  | σ_u_0 u_1 (0) | 0.162 | 0.162 | NA | 0.522 | 0.106 | 0.457 | 0.457 | NA | 0.362 | 0.329 | 0.368 | 0.368 | NA | 0.260 | 0.301 |
|  | σ^2_(u_1 ) (10) | 9.780 | -0.220 | -2.20% | 0.448 | 0.456 | 10.002 | 0.002 | 0.016% | 0.323 | 0.389 | 9.747 | -0.253 | -2.53% | 0.226 | 0.275 |
|  | σ_(v_L ) (2.2361) | 2.852 | -1.391 | -32.79% | 0.547 | 0.531 | 2.480 | -1.763 | -41.55% | 0.323 | 0.124 | 2.349 | -1.894 | -44.64% | 0.217 | 0.128 |
|  | σ_(v_S ) (0.6325) | 0.199 | -0.433 | -68.50% | 0.073 | 0.014 | 0.317 | -0.315 | -49.81% | 0.061 | 0.085 | 0.240 | -0.392 | -62.02% | 0.040 | 0.038 |
|  | σ_ε (5.7446) | 5.744 | 0.000 | -0.01% | 0.032 | 0.041 | 5.742 | -0.003 | -0.05% | 0.022 | 0.018 | 5.742 | -0.003 | -0.05% | 0.016 | 0.005 |

Table A 1‑8 Results of the proposed MJM censoring rate 60%, ICC at the level 3 in the longitudinal submodel = 0.3, group-level variance in the survival submodel = 1

|  | L3 ICC in longitudinal outcome = 0.3; variance at L2 in survival = 1 | | | | | | | | | | | | | | | |
| --- | --- | --- | --- | --- | --- | --- | --- | --- | --- | --- | --- | --- | --- | --- | --- | --- |
|  | Parameter | Mean | Bias | RB | MESE | ESE | Mean | Bias | RB | MESE | ESE | Mean | Bias | RB | MESE | ESE |
|  |  | n = 50 | | | | | n = 100 | | | | | n = 200 | | | | |
| K=11 | α (-0.2) | -0.138 | 0.062 | -31.01% | 0.008 | 0.017 | -0.134 | 0.066 | -33.09% | 0.005 | 0.003 | -0.133 | 0.067 | -33.59% | 0.004 | 0.002 |
|  | β_000 (16) | 16.015 | 0.015 | 9.39E-04 | 0.728 | 0.362 | 15.667 | -0.333 | -2.08% | 0.508 | 0.464 | 15.696 | -0.304 | -1.90% | 0.330 | 0.407 |
|  | β_100 (-0.8) | -0.917 | -0.117 | 14.57% | 0.129 | 0.069 | -0.942 | -0.142 | 17.78% | 0.094 | 0.138 | -1.014 | -0.214 | 26.77% | 0.067 | 0.118 |
|  | β_001 (1.6) | 1.341 | -0.259 | -16.21% | 0.335 | 0.888 | 1.629 | 0.029 | 1.80% | 0.242 | 0.377 | 1.881 | 0.281 | 17.58% | 0.167 | 0.686 |
|  | β_101 (-2) | -1.707 | 0.293 | -14.65% | 0.181 | 0.119 | -1.660 | 0.340 | -16.99% | 0.128 | 0.074 | -1.615 | 0.385 | -19.23% | 0.090 | 0.084 |
|  | σ^2_(u_0) (10) | 27.930 | 17.930 | 179.30% | 1.419 | 3.887 | 26.003 | 16.003 | 160.03% | 0.957 | 0.728 | 26.634 | 16.634 | 166.34% | 0.687 | 1.810 |
|  | σ_u_0 u_1 (0) | -0.274 | -0.274 | NA | 0.566 | 0.325 | -0.305 | -0.305 | NA | 0.398 | 0.381 | -0.128 | -0.128 | NA | 0.281 | 0.147 |
|  | σ^2_(u_1 ) (10) | 10.550 | 0.550 | 5.50% | 0.478 | 0.550 | 10.721 | 0.721 | 7.21% | 0.342 | 0.388 | 10.645 | 0.645 | 6.45% | 0.242 | 0.185 |
|  | σ_(v_L ) (2.2361) | 1.495 | -2.747 | -64.75% | 0.695 | 0.737 | 1.449 | -2.794 | -65.85% | 0.413 | 0.062 | 1.276 | -2.967 | -69.93% | 0.254 | 0.114 |
|  | σ_(v_S ) (0.6325) | 0.324 | -0.676 | -67.58% | 0.161 | 0.126 | 0.333 | -0.667 | -66.68% | 0.098 | 0.089 | 0.279 | -0.721 | -72.09% | 0.060 | 0.041 |
|  | σ_ε (5.7446) | 5.738 | -0.006 | -0.11% | 0.032 | 0.040 | 5.731 | -0.013 | -0.23% | 0.022 | 0.021 | 5.738 | -0.006 | -0.11% | 0.016 | 0.004 |
| K=7 | α (-0.2) | -0.140 | 0.060 | -29.99% | 0.008 | 0.016 | -0.136 | 0.064 | -32.02% | 0.005 | 0.004 | -0.135 | 0.065 | -32.37% | 0.003 | 0.006 |
|  | β_000 (16) | 16.000 | 0.000 | 0.001% | 0.694 | 0.080 | 15.700 | -0.300 | -1.88% | 0.472 | 0.577 | 15.698 | -0.302 | -1.89% | 0.312 | 0.407 |
|  | β_100 (-0.8) | -0.876 | -0.076 | 9.52% | 0.129 | 0.062 | -0.898 | -0.098 | 12.27% | 0.093 | 0.130 | -0.927 | -0.127 | 15.85% | 0.068 | 0.140 |
|  | β_001 (1.6) | 1.295 | -0.305 | -19.08% | 0.335 | 0.654 | 1.489 | -0.111 | -6.97% | 0.247 | 0.539 | 1.810 | 0.210 | 13.13% | 0.174 | 0.594 |
|  | β_101 (-2) | -1.736 | 0.264 | -13.19% | 0.180 | 0.135 | -1.693 | 0.307 | -15.37% | 0.128 | 0.074 | -1.655 | 0.345 | -17.27% | 0.091 | 0.096 |
|  | σ^2_(u_0) (10) | 27.322 | 17.322 | 173.22% | 1.393 | 4.322 | 24.919 | 14.919 | 149.19% | 0.931 | 0.620 | 25.659 | 15.659 | 156.59% | 0.688 | 1.319 |
|  | σ_u_0 u_1 (0) | -0.184 | -0.184 | NA | 0.559 | 0.276 | -0.178 | -0.178 | NA | 0.390 | 0.292 | -0.150 | -0.150 | NA | 0.280 | 0.208 |
|  | σ^2_(u_1 ) (10) | 10.436 | 0.436 | 4.36% | 0.473 | 0.543 | 10.584 | 0.584 | 5.84% | 0.339 | 0.360 | 10.348 | 0.348 | 3.48% | 0.238 | 0.220 |
|  | σ_(v_L ) (2.2361) | 1.784 | -2.459 | -57.96% | 0.610 | 0.681 | 1.749 | -2.494 | -58.78% | 0.364 | 0.328 | 1.504 | -2.738 | -64.54% | 0.228 | 0.093 |
|  | σ_(v_S ) (0.6325) | 0.349 | -0.651 | -65.15% | 0.133 | 0.079 | 0.405 | -0.595 | -59.52% | 0.093 | 0.067 | 0.288 | -0.712 | -71.15% | 0.049 | 0.037 |
|  | σ_ε (5.7446) | 5.738 | -0.006 | -0.11% | 0.032 | 0.041 | 5.732 | -0.013 | -0.22% | 0.022 | 0.021 | 5.738 | -0.006 | -0.11% | 0.017 | 0.005 |
| K=3 | α (-0.2) | -0.126 | 0.074 | -36.82% | 0.007 | 0.008 | -0.129 | 0.071 | -35.42% | 0.005 | 0.004 | -0.121 | 0.079 | -39.26% | 0.003 | 0.003 |
|  | β_000 (16) | 16.156 | 0.156 | 0.98% | 0.694 | 0.204 | 15.581 | -0.419 | -2.62% | 0.463 | 0.399 | 15.599 | -0.401 | -2.51% | 0.306 | 0.309 |
|  | β_100 (-0.8) | -0.659 | 0.141 | -17.66% | 0.126 | 0.036 | -0.698 | 0.102 | -12.69% | 0.092 | 0.146 | -0.754 | 0.046 | -5.70% | 0.067 | 0.132 |
|  | β_001 (1.6) | 0.911 | -0.689 | -43.09% | 0.348 | 0.405 | 1.595 | -0.005 | -0.30% | 0.258 | 0.321 | 1.895 | 0.295 | 18.46% | 0.182 | 0.446 |
|  | β_101 (-2) | -1.800 | 0.200 | -10.00% | 0.178 | 0.127 | -1.775 | 0.225 | -11.23% | 0.127 | 0.103 | -1.729 | 0.271 | -13.53% | 0.090 | 0.081 |
|  | σ^2_(u_0) (10) | 23.101 | 13.101 | 131.01% | 1.236 | 2.906 | 21.507 | 11.507 | 115.07% | 0.838 | 0.189 | 23.074 | 13.074 | 130.74% | 0.620 | 1.314 |
|  | σ_u_0 u_1 (0) | -0.022 | -0.022 | NA | 0.519 | 0.213 | 0.104 | 0.104 | NA | 0.363 | 0.389 | 0.232 | 0.232 | NA | 0.261 | 0.059 |
|  | σ^2_(u_1 ) (10) | 9.738 | -0.262 | -2.62% | 0.445 | 0.543 | 9.964 | -0.036 | -0.36% | 0.322 | 0.403 | 9.884 | -0.116 | -1.16% | 0.229 | 0.246 |
|  | σ_(v_L ) (2.2361) | 2.790 | -1.453 | -34.25% | 0.532 | 0.501 | 2.603 | -1.639 | -38.64% | 0.336 | 0.157 | 2.366 | -1.877 | -44.24% | 0.218 | 0.122 |
|  | σ_(v_S ) (0.6325) | 0.282 | -0.718 | -71.84% | 0.082 | 0.020 | 0.415 | -0.585 | -58.53% | 0.068 | 0.080 | 0.318 | -0.682 | -68.20% | 0.042 | 0.057 |
|  | σ_ε (5.7446) | 5.741 | -0.003 | -0.06% | 0.032 | 0.040 | 5.736 | -0.009 | -0.16% | 0.022 | 0.021 | 5.741 | -0.003 | -0.06% | 0.016 | 0.003 |

# Appendix 2 Simulation studies to evaluate the impact of ignoring the group level

Table A 2‑1 Results of the proposed MJM versus standard JM censoring rate 20%, n = 50

|  |  | Proposed MJM | | | | | Standard JM | | | | |
| --- | --- | --- | --- | --- | --- | --- | --- | --- | --- | --- | --- |
|  | Parameter | Mean | Bias | RB | MESE | ESE | Mean | Bias | RB | MESE | ESE |
| L3 ICC in longitudinal outcome = 0.1; variance at L2 in survival = 0.4 | α (-0.2) | -0.168 | 0.032 | -15.78% | 0.008 | 0.007 | -0.158 | 0.042 | -21.06% | 0.007 | 0.01 |
|  | β_000 (16) | 16.104 | 0.104 | 0.65% | 0.433 | 0.139 | 16.042 | 0.042 | 0.26% | 0.189 | 0.509 |
|  | β_100 (-0.8) | -0.611 | 0.189 | -23.68% | 0.163 | 0.126 | -0.289 | 0.511 | -63.89% | 0.055 | 0.186 |
|  | β_001 (1.6) | 1.401 | -0.199 | -12.46% | 0.298 | 0.370 | 1.364 | -0.236 | -14.77% | 0.271 | 0.704 |
|  | β_101 (-2) | -1.811 | 0.189 | -9.45% | 0.224 | 0.155 | -1.556 | 0.444 | -22.21% | 0.119 | 0.222 |
|  | σ^2_(u_0) (10) | 16.046 | 6.046 | 60.46% | 1.155 | 2.440 | 13.466 | 3.466 | 34.66% | NA | 1.423 |
|  | σ_u_0 u_1 (0) | -0.943 | -0.943 | NA | 0.622 | 0.398 | 4.357 | 4.357 | NA | NA | 0.634 |
|  | σ^2_(u_1 ) (10) | 10.246 | 0.246 | 2.46% | 0.602 | 0.252 | 8.563 | -1.437 | -14.37% | NA | 0.637 |
|  | σ_(v_L ) (2.2361) | 1.062 | -1.175 | -52.53% | 0.373 | 0.363 |  |  |  |  |  |
|  | σ_(v_S ) (0.6325) | 0.274 | -0.359 | -56.73% | 0.092 | 0.068 |  |  |  |  |  |
|  | σ_ε (5.7446) | 5.700 | -0.045 | -0.78% | 0.044 | 0.041 | 5.742 | -0.003 | -0.05% | NA | 0.043 |
| L3 ICC in longitudinal outcome = 0.1; variance at L2 in survival = 1 | α (-0.2) | -0.149 | 0.051 | -25.72% | 0.007 | 0.004 | -0.14 | 0.060 | -29.82% | 0.006 | 0.012 |
|  | β_000 (16) | 16.183 | 0.183 | 1.15% | 0.510 | 0.125 | 16.029 | 0.029 | 0.18% | 0.191 | 0.519 |
|  | β_100 (-0.8) | -0.544 | 0.256 | -32.00% | 0.164 | 0.162 | -0.271 | 0.529 | -66.09% | 0.056 | 0.207 |
|  | β_001 (1.6) | 1.199 | -0.401 | -25.04% | 0.303 | 0.243 | 1.384 | -0.216 | -13.51% | 0.273 | 0.724 |
|  | β_101 (-2) | -1.787 | 0.213 | -10.64% | 0.228 | 0.052 | -1.661 | 0.339 | -16.95% | 0.118 | 0.249 |
|  | σ^2_(u_0) (10) | 16.675 | 6.675 | 66.75% | 1.189 | 1.539 | 13.457 | 3.457 | 34.57% | NA | 1.447 |
|  | σ_u_0 u_1 (0) | -1.357 | -1.357 | NA | 0.646 | 0.460 | 5.099 | 5.099 | NA | NA | 0.582 |
|  | σ^2_(u_1 ) (10) | 10.261 | 0.261 | 2.61% | 0.604 | 0.183 | 8.537 | -1.463 | -14.63% | NA | 0.602 |
|  | σ_(v_L ) (2.2361) | 1.156 | -1.080 | -48.29% | 0.459 | 0.574 |  |  |  |  |  |
|  | σ_(v_S ) (0.6325) | 0.265 | -0.735 | -73.54% | 0.093 | 0.065 |  |  |  |  |  |
|  | σ_ε (5.7446) | 5.695 | -0.050 | -0.86% | 0.044 | 0.030 | 5.742 | -0.003 | -0.05% | NA | 0.045 |
| L3 ICC in longitudinal outcome = 0.3; variance at L2 in survival = 0.4 | α (-0.2) | -0.162 | 0.038 | -19.03% | 0.007 | 0.006 | -0.157 | 0.043 | -21.71% | 0.006 | 0.01 |
|  | β_000 (16) | 16.126 | 0.126 | 0.79% | 0.897 | 0.252 | 16.218 | 0.218 | 1.36% | 0.221 | 0.940 |
|  | β_100 (-0.8) | -0.521 | 0.279 | -34.87% | 0.164 | 0.122 | -0.235 | 0.565 | -70.68% | 0.058 | 0.215 |
|  | β_001 (1.6) | 1.106 | -0.494 | -30.86% | 0.359 | 1.003 | 1.257 | -0.343 | -21.44% | 0.321 | 1.262 |
|  | β_101 (-2) | -1.820 | 0.180 | -8.99% | 0.227 | 0.106 | -1.654 | 0.346 | -17.31% | 0.115 | 0.248 |
|  | σ^2_(u_0) (10) | 26.804 | 16.804 | 168.04% | 1.581 | 3.518 | 25.643 | 15.643 | 156.43% | NA | 3.615 |
|  | σ_u_0 u_1 (0) | -1.622 | -1.622 | NA | 0.762 | 1.028 | 0.285 | 0.285 | NA | NA | 0.827 |
|  | σ^2_(u_1 ) (10) | 10.408 | 0.408 | 4.08% | 0.611 | 0.168 | 8.548 | -1.452 | -14.52% | NA | 0.603 |
|  | σ_(v_L ) (2.2361) | 2.404 | -1.839 | -43.33% | 0.766 | 0.364 |  |  |  |  |  |
|  | σ_(v_S ) (0.6325) | 0.244 | -0.389 | -61.49% | 0.086 | 0.044 |  |  |  |  |  |
|  | σ_ε (5.7446) | 5.693 | -0.052 | -0.90% | 0.044 | 0.023 | 5.733 | -0.011 | -0.20% | NA | 0.044 |
| L3 ICC in longitudinal outcome = 0.3; variance at L2 in survival = 1 | α (-0.2) | -0.147 | 0.053 | -26.52% | 0.007 | 0.002 | -0.137 | 0.063 | -31.27% | 0.006 | 0.012 |
|  | β_000 (16) | 16.208 | 0.208 | 1.30% | 0.795 | 0.475 | 16.180 | 0.180 | 1.12% | 0.224 | 0.944 |
|  | β_100 (-0.8) | -0.600 | 0.200 | -24.95% | 0.167 | 0.139 | -0.211 | 0.589 | -73.59% | 0.060 | 0.202 |
|  | β_001 (1.6) | 1.178 | -0.422 | -26.39% | 0.364 | 1.253 | 1.344 | -0.256 | -15.99% | 0.323 | 1.280 |
|  | β_101 (-2) | -1.778 | 0.222 | -11.11% | 0.230 | 0.126 | -1.745 | 0.255 | -12.74% | 0.109 | 0.258 |
|  | σ^2_(u_0) (10) | 27.552 | 17.552 | 175.52% | 1.630 | 3.269 | 25.734 | 15.734 | 157.34% | NA | 3.575 |
|  | σ_u_0 u_1 (0) | -1.249 | -1.249 | NA | 0.790 | 0.610 | 2.094 | 2.094 | NA | NA | 0.785 |
|  | σ^2_(u_1 ) (10) | 10.451 | 0.451 | 4.51% | 0.616 | 0.157 | 8.527 | -1.473 | -14.73% | NA | 0.584 |
|  | σ_(v_L ) (2.2361) | 2.081 | -2.162 | -50.95% | 0.687 | 1.074 |  |  |  |  |  |
|  | σ_(v_S ) (0.6325) | 0.311 | -0.689 | -68.92% | 0.106 | 0.092 |  |  |  |  |  |
|  | σ_ε (5.7446) | 5.709 | -0.036 | -0.62% | 0.045 | 0.040 | 5.735 | -0.010 | -0.17% | NA | 0.045 |

Table A 2‑2 Results of the proposed MJM versus standard JM censoring rate 20%, n = 100

|  |  | Proposed MJM | | | | | Standard JM | | | | |
| --- | --- | --- | --- | --- | --- | --- | --- | --- | --- | --- | --- |
|  | Parameter | Mean | Bias | RB | MESE | ESE | Mean | Bias | RB | MESE | ESE |
| L3 ICC in longitudinal outcome = 0.1; variance at L2 in survival = 0.4 | α (-0.2) | -0.165 | 0.035 | -17.63% | 0.006 | 0.007 | -0.156 | 0.044 | -21.99% | 0.005 | 0.007 |
|  | β_000 (16) | 15.929 | -0.071 | -0.45% | 0.300 | 0.364 | 16.048 | 0.048 | 0.30% | 0.136 | 0.355 |
|  | β_100 (-0.8) | -0.597 | 0.203 | -25.41% | 0.120 | 0.319 | -0.257 | 0.543 | -67.81% | 0.040 | 0.144 |
|  | β_001 (1.6) | 1.274 | -0.326 | -20.35% | 0.216 | 0.374 | 1.319 | -0.281 | -17.56% | 0.193 | 0.495 |
|  | β_101 (-2) | -1.720 | 0.280 | -14.01% | 0.159 | 0.212 | -1.560 | 0.440 | -22.02% | 0.087 | 0.169 |
|  | σ^2_(u_0) (10) | 13.981 | 3.981 | 39.81% | 0.766 | 0.153 | 13.830 | 3.830 | 38.30% | NA | 1.116 |
|  | σ_u_0 u_1 (0) | -0.761 | -0.761 | NA | 0.427 | 0.137 | 2.372 | 2.372 | NA | NA | 0.449 |
|  | σ^2_(u_1 ) (10) | 10.248 | 0.248 | 2.48% | 0.433 | 0.589 | 8.606 | -1.394 | -13.94% | NA | 0.419 |
|  | σ_(v_L ) (2.2361) | 1.108 | -1.128 | -50.45% | 0.235 | 0.064 |  |  |  |  |  |
|  | σ_(v_S ) (0.6325) | 0.262 | -0.371 | -58.62% | 0.055 | 0.090 |  |  |  |  |  |
|  | σ_ε (5.7446) | 5.725 | -0.020 | -0.35% | 0.032 | 0.016 | 5.742 | -0.002 | -0.04% | NA | 0.031 |
| L3 ICC in longitudinal outcome = 0.1; variance at L2 in survival = 1 | α (-0.2) | -0.145 | 0.055 | -27.57% | 0.005 | 0.007 | -0.136 | 0.064 | -31.85% | 0.004 | 0.008 |
|  | β_000 (16) | 15.879 | -0.121 | -0.75% | 0.317 | 0.220 | 16.021 | 0.021 | 0.13% | 0.137 | 0.355 |
|  | β_100 (-0.8) | -0.563 | 0.237 | -29.63% | 0.121 | 0.211 | -0.233 | 0.567 | -70.84% | 0.041 | 0.144 |
|  | β_001 (1.6) | 1.282 | -0.318 | -19.86% | 0.223 | 0.337 | 1.375 | -0.225 | -14.08% | 0.194 | 0.500 |
|  | β_101 (-2) | -1.739 | 0.261 | -13.06% | 0.161 | 0.208 | -1.691 | 0.309 | -15.44% | 0.085 | 0.178 |
|  | σ^2_(u_0) (10) | 14.220 | 4.220 | 42.20% | 0.778 | 0.865 | 13.717 | 3.717 | 37.17% | NA | 1.097 |
|  | σ_u_0 u_1 (0) | -0.675 | -0.675 | NA | 0.432 | 0.429 | 4.196 | 4.196 | NA | NA | 0.448 |
|  | σ^2_(u_1 ) (10) | 10.172 | 0.172 | 1.72% | 0.430 | 0.458 | 8.567 | -1.433 | -14.33% | NA | 0.435 |
|  | σ_(v_L ) (2.2361) | 1.206 | -1.030 | -46.06% | 0.254 | 0.141 |  |  |  |  |  |
|  | σ_(v_S ) (0.6325) | 0.345 | -0.655 | -65.53% | 0.067 | 0.040 |  |  |  |  |  |
|  | σ_ε (5.7446) | 5.726 | -0.018 | -0.32% | 0.032 | 0.025 | 5.743 | -0.001 | -0.02% | NA | 0.030 |
| L3 ICC in longitudinal outcome = 0.3; variance at L2 in survival = 0.4 | α (-0.2) | -0.164 | 0.036 | -17.89% | 0.005 | 0.004 | -0.154 | 0.046 | -23.05% | 0.005 | 0.007 |
|  | β_000 (16) | 15.645 | -0.355 | -2.216% | 0.557 | 0.491 | 16.204 | 0.204 | 1.27% | 0.159 | 0.617 |
|  | β_100 (-0.8) | -0.519 | 0.281 | -35.15% | 0.123 | 0.197 | -0.178 | 0.622 | -77.76% | 0.042 | 0.160 |
|  | β_001 (1.6) | 1.474 | -0.126 | -7.85% | 0.269 | 0.055 | 1.194 | -0.406 | -25.35% | 0.229 | 0.879 |
|  | β_101 (-2) | -1.827 | 0.173 | -8.65% | 0.163 | 0.178 | -1.672 | 0.328 | -16.38% | 0.081 | 0.195 |
|  | σ^2_(u_0) (10) | 24.595 | 14.595 | 145.95% | 1.075 | 0.152 | 26.423 | 16.423 | 164.23% | NA | 2.922 |
|  | σ_u_0 u_1 (0) | -1.555 | -1.555 | NA | 0.539 | 0.223 | -2.878 | -2.878 | NA | NA | 0.633 |
|  | σ^2_(u_1 ) (10) | 10.358 | 0.358 | 3.58% | 0.442 | 0.476 | 8.522 | -1.478 | -14.78% | NA | 0.431 |
|  | σ_(v_L ) (2.2361) | 2.342 | -1.901 | -44.81% | 0.428 | 0.291 |  |  |  |  |  |
|  | σ_(v_S ) (0.6325) | 0.280 | -0.353 | -55.80% | 0.057 | 0.033 |  |  |  |  |  |
|  | σ_ε (5.7446) | 5.717 | -0.027 | -0.48% | 0.032 | 0.025 | 5.735 | -0.009 | -0.16% | NA | 0.029 |
| L3 ICC in longitudinal outcome = 0.3; variance at L2 in survival = 1 | α (-0.2) | -0.146 | 0.054 | -27.19% | 0.005 | 0.009 | -0.134 | 0.066 | -32.86% | 0.004 | 0.008 |
|  | β_000 (16) | 15.554 | -0.446 | -2.79% | 0.516 | 0.469 | 16.176 | 0.176 | 1.10% | 0.161 | 0.620 |
|  | β_100 (-0.8) | -0.534 | 0.266 | -33.26% | 0.124 | 0.287 | -0.173 | 0.627 | -78.32% | 0.044 | 0.155 |
|  | β_001 (1.6) | 1.455 | -0.145 | -9.06% | 0.272 | 0.637 | 1.279 | -0.321 | -20.06% | 0.230 | 0.876 |
|  | β_101 (-2) | -1.771 | 0.229 | -11.47% | 0.164 | 0.227 | -1.758 | 0.242 | -12.10% | 0.076 | 0.200 |
|  | σ^2_(u_0) (10) | 25.988 | 15.988 | 159.88% | 1.118 | 0.767 | 26.394 | 16.394 | 163.94% | NA | 2.979 |
|  | σ_u_0 u_1 (0) | -1.654 | -1.654 | NA | 0.555 | 0.522 | -0.989 | -0.989 | NA | NA | 0.608 |
|  | σ^2_(u_1 ) (10) | 10.388 | 0.388 | 3.88% | 0.443 | 0.432 | 8.586 | -1.414 | -14.14% | NA | 0.418 |
|  | σ_(v_L ) (2.2361) | 2.117 | -2.126 | -50.10% | 0.399 | 0.326 |  |  |  |  |  |
|  | σ_(v_S ) (0.6325) | 0.352 | -0.648 | -64.85% | 0.069 | 0.060 |  |  |  |  |  |
|  | σ_ε (5.7446) | 5.726 | -0.018 | -0.32% | 0.032 | 0.017 | 5.733 | -0.012 | -0.20% | NA | 0.030 |

Table A 2‑3 Results of the proposed MJM versus standard JM censoring rate 20%, n = 200

|  |  | Proposed MJM | | | | | Standard JM | | | | |
| --- | --- | --- | --- | --- | --- | --- | --- | --- | --- | --- | --- |
|  | Parameter | Mean | Bias | RB | MESE | ESE | Mean | Bias | RB | MESE | ESE |
| L3 ICC in longitudinal outcome = 0.1; variance at L2 in survival = 0.4 | α (-0.2) | -0.161 | 0.039 | -19.67% | 0.004 | 0.003 | -0.156 | 0.044 | -22.15% | 0.003 | 0.005 |
|  | β_000 (16) | 15.946 | -0.054 | -0.34% | 0.184 | 0.279 | 16.070 | 0.070 | 0.44% | 0.096 | 0.247 |
|  | β_100 (-0.8) | -0.607 | 0.193 | -24.18% | 0.086 | 0.085 | -0.234 | 0.566 | -70.80% | 0.028 | 0.088 |
|  | β_001 (1.6) | 1.615 | 0.015 | 0.92% | 0.148 | 0.481 | 1.323 | -0.277 | -17.29% | 0.136 | 0.364 |
|  | β_101 (-2) | -1.698 | 0.302 | -15.10% | 0.112 | 0.088 | -1.572 | 0.428 | -21.40% | 0.062 | 0.105 |
|  | σ^2_(u_0) (10) | 14.381 | 4.381 | 43.81% | 0.547 | 0.512 | 13.940 | 3.940 | 39.40% | NA | 0.743 |
|  | σ_u_0 u_1 (0) | -0.817 | -0.817 | NA | 0.304 | 0.403 | 1.915 | 1.915 | NA | NA | 0.312 |
|  | σ^2_(u_1 ) (10) | 9.995 | -0.005 | -0.05% | 0.302 | 0.159 | 8.595 | -1.405 | -14.05% | NA | 0.310 |
|  | σ_(v_L ) (2.2361) | 0.788 | -1.448 | -64.76% | 0.139 | 0.137 |  |  |  |  |  |
|  | σ_(v_S ) (0.6325) | 0.192 | -0.441 | -69.71% | 0.030 | 0.034 |  |  |  |  |  |
|  | σ_ε (5.7446) | 5.731 | -0.014 | -0.24% | 0.023 | 0.010 | 5.744 | -0.001 | -0.02% | NA | 0.022 |
| L3 ICC in longitudinal outcome = 0.1; variance at L2 in survival = 1 | α (-0.2) | -0.146 | 0.054 | -26.85% | 0.004 | 0.002 | -0.136 | 0.064 | -31.83% | 0.003 | 0.006 |
|  | β_000 (16) | 15.890 | -0.110 | -0.69% | 0.183 | 0.228 | 16.045 | 0.045 | 0.28% | 0.097 | 0.248 |
|  | β_100 (-0.8) | -0.613 | 0.187 | -23.33% | 0.088 | 0.114 | -0.211 | 0.589 | -73.63% | 0.029 | 0.098 |
|  | β_001 (1.6) | 1.661 | 0.061 | 3.84% | 0.149 | 0.376 | 1.380 | -0.220 | -13.73% | 0.137 | 0.363 |
|  | β_101 (-2) | -1.684 | 0.316 | -15.79% | 0.114 | 0.044 | -1.700 | 0.300 | -15.02% | 0.061 | 0.119 |
|  | σ^2_(u_0) (10) | 14.387 | 4.387 | 43.87% | 0.553 | 0.618 | 13.859 | 3.859 | 38.59% | NA | 0.747 |
|  | σ_u_0 u_1 (0) | -0.710 | -0.710 | NA | 0.308 | 0.287 | 3.892 | 3.892 | NA | NA | 0.312 |
|  | σ^2_(u_1 ) (10) | 10.071 | 0.071 | 0.71% | 0.306 | 0.116 | 8.537 | -1.463 | -14.63% | NA | 0.310 |
|  | σ_(v_L ) (2.2361) | 0.818 | -1.418 | -63.41% | 0.141 | 0.070 |  |  |  |  |  |
|  | σ_(v_S ) (0.6325) | 0.267 | -0.733 | -73.32% | 0.038 | 0.032 |  |  |  |  |  |
|  | σ_ε (5.7446) | 5.740 | -0.005 | -0.09% | 0.023 | 0.007 | 5.744 | -0.001 | -0.01% | NA | 0.022 |
| L3 ICC in longitudinal outcome = 0.3; variance at L2 in survival = 0.4 | α (-0.2) | -0.164 | 0.036 | -18.22% | 0.004 | 0.002 | -0.154 | 0.046 | -22.95% | 0.003 | 0.005 |
|  | β_000 (16) | 15.859 | -0.141 | -0.88% | 0.319 | 0.414 | 16.260 | 0.260 | 1.63% | 0.113 | 0.443 |
|  | β_100 (-0.8) | -0.640 | 0.160 | -19.96% | 0.088 | 0.099 | -0.165 | 0.635 | -79.32% | 0.030 | 0.105 |
|  | β_001 (1.6) | 1.667 | 0.067 | 4.22% | 0.175 | 0.543 | 1.184 | -0.416 | -25.99% | 0.162 | 0.640 |
|  | β_101 (-2) | -1.600 | 0.400 | -19.98% | 0.114 | 0.050 | -1.666 | 0.334 | -16.68% | 0.058 | 0.130 |
|  | σ^2_(u_0) (10) | 25.183 | 15.183 | 151.83% | 0.772 | 1.973 | 26.632 | 16.632 | 166.32% | NA | 1.849 |
|  | σ_u_0 u_1 (0) | -1.496 | -1.496 | NA | 0.380 | 0.029 | -4.332 | -4.332 | NA | NA | 0.438 |
|  | σ^2_(u_1 ) (10) | 9.992 | -0.008 | -0.08% | 0.304 | 0.336 | 8.549 | -1.451 | -14.51% | NA | 0.306 |
|  | σ_(v_L ) (2.2361) | 1.757 | -2.486 | -58.59% | 0.238 | 0.107 |  |  |  |  |  |
|  | σ_(v_S ) (0.6325) | 0.213 | -0.419 | -66.32% | 0.033 | 0.014 |  |  |  |  |  |
|  | σ_ε (5.7446) | 5.742 | -0.003 | -0.05% | 0.023 | 0.005 | 5.737 | -0.008 | -0.13% | NA | 0.023 |
| L3 ICC in longitudinal outcome = 0.3; variance at L2 in survival = 1 | α (-0.2) | -0.144 | 0.056 | -27.87% | 0.003 | 0.003 | -0.134 | 0.066 | -32.90% | 0.003 | 0.006 |
|  | β_000 (16) | 15.819 | -0.181 | -1.13% | 0.350 | 0.389 | 16.224 | 0.224 | 1.40% | 0.114 | 0.457 |
|  | β_100 (-0.8) | -0.653 | 0.147 | -18.34% | 0.090 | 0.041 | -0.154 | 0.646 | -80.78% | 0.031 | 0.103 |
|  | β_001 (1.6) | 1.769 | 0.169 | 10.53% | 0.177 | 0.614 | 1.267 | -0.333 | -20.81% | 0.163 | 0.646 |
|  | β_101 (-2) | -1.669 | 0.331 | -16.57% | 0.117 | 0.019 | -1.758 | 0.242 | -12.08% | 0.054 | 0.135 |
|  | σ^2_(u_0) (10) | 24.896 | 14.896 | 148.96% | 0.767 | 1.084 | 26.685 | 16.685 | 166.85% | NA | 1.932 |
|  | σ_u_0 u_1 (0) | -1.188 | -1.188 | NA | 0.389 | 0.493 | -1.366 | -1.366 | NA | NA | 0.419 |
|  | σ^2_(u_1 ) (10) | 10.467 | 0.467 | 4.67% | 0.317 | 0.187 | 8.559 | -1.441 | -14.41% | NA | 0.289 |
|  | σ_(v_L ) (2.2361) | 1.879 | -2.363 | -55.70% | 0.258 | 0.122 |  |  |  |  |  |
|  | σ_(v_S ) (0.6325) | 0.284 | -0.716 | -71.56% | 0.040 | 0.028 |  |  |  |  |  |
|  | σ_ε (5.7446) | 5.743 | -0.001 | -0.02% | 0.023 | 0.009 | 5.736 | -0.009 | -0.15% | NA | 0.022 |

Table A 2‑4 Results of the proposed MJM versus standard JM censoring rate 60%, n = 50

|  |  | Proposed MJM | | | | | Standard JM | | | | |
| --- | --- | --- | --- | --- | --- | --- | --- | --- | --- | --- | --- |
|  | Parameter | Mean | Bias | RB | MESE | ESE | Mean | Bias | RB | MESE | ESE |
| L3 ICC in longitudinal outcome = 0.1; variance at L2 in survival = 0.4 | α (-0.2) | -0.142 | 0.058 | -28.93% | 0.007 | 0.005 | -0.101 | 0.099 | -49.58% | 0.004 | 0.009 |
|  | β_000 (16) | 16.019 | 0.019 | 0.12% | 0.601 | 0.223 | 16.026 | 0.026 | 0.16% | 0.178 | 0.504 |
|  | β_100 (-0.8) | -0.923 | -0.123 | 15.37% | 0.130 | 0.020 | -0.280 | 0.520 | -65.02% | 0.051 | 0.178 |
|  | β_001 (1.6) | 1.482 | -0.118 | -7.37% | 0.275 | 0.435 | 1.593 | -0.007 | -0.41% | 0.252 | 0.687 |
|  | β_101 (-2) | -1.677 | 0.323 | -16.13% | 0.183 | 0.121 | -2.005 | -0.005 | 0.26% | 0.070 | 0.220 |
|  | σ^2_(u_0) (10) | 15.131 | 5.131 | 51.31% | 0.956 | 1.734 | 15.009 | 5.009 | 50.09% | NA | 1.440 |
|  | σ_u_0 u_1 (0) | -0.570 | -0.570 | NA | 0.476 | 0.154 | 0.257 | 0.257 | NA | NA | 0.525 |
|  | σ^2_(u_1 ) (10) | 10.761 | 0.761 | 7.61% | 0.484 | 0.288 | 10.33 | 0.333 | 3.33% | NA | 0.667 |
|  | σ_(v_L ) (2.2361) | 0.963 | -1.273 | -56.95% | 0.603 | 0.316 |  |  |  |  |  |
|  | σ_(v_S ) (0.6325) | 0.210 | -0.422 | -66.79% | 0.109 | 0.055 |  |  |  |  |  |
|  | σ_ε (5.7446) | 5.747 | 0.003 | 0.01% | 0.033 | 0.047 | 5.716 | -0.028 | -0.49% | NA | 0.034 |
| L3 ICC in longitudinal outcome = 0.1; variance at L2 in survival = 1 | α (-0.2) | -0.133 | 0.067 | -33.74% | 0.008 | 0.016 | -0.093 | 0.107 | -53.41% | 0.005 | 0.007 |
|  | β_000 (16) | 15.938 | -0.062 | -0.39% | 0.452 | 0.239 | 16.061 | 0.061 | 0.38% | 0.176 | 0.526 |
|  | β_100 (-0.8) | -0.934 | -0.134 | 16.73% | 0.130 | 0.052 | -0.085 | 0.715 | -89.41% | 0.053 | 0.146 |
|  | β_001 (1.6) | 1.559 | -0.041 | -2.56% | 0.275 | 0.530 | 1.622 | 0.022 | 1.38% | 0.250 | 0.681 |
|  | β_101 (-2) | -1.711 | 0.289 | -14.45% | 0.182 | 0.153 | -2.026 | -0.026 | 1.31% | 0.073 | 0.201 |
|  | σ^2_(u_0) (10) | 15.531 | 5.531 | 55.31% | 0.941 | 1.785 | 14.715 | 4.715 | 47.15% | NA | 1.351 |
|  | σ_u_0 u_1 (0) | -0.136 | -0.136 | NA | 0.471 | 0.402 | -0.637 | -0.637 | NA | NA | 0.500 |
|  | σ^2_(u_1 ) (10) | 10.717 | 0.717 | 7.17% | 0.486 | 0.522 | 11.546 | 1.546 | 15.46% | NA | 0.622 |
|  | σ_(v_L ) (2.2361) | 0.762 | -1.474 | -65.93% | 0.425 | 0.444 |  |  |  |  |  |
|  | σ_(v_S ) (0.6325) | 0.306 | -0.694 | -69.39% | 0.155 | 0.111 |  |  |  |  |  |
|  | σ_ε (5.7446) | 5.736 | -0.009 | -0.15% | 0.032 | 0.034 | 5.722 | -0.023 | -0.40% | NA | 0.032 |
| L3 ICC in longitudinal outcome = 0.3; variance at L2 in survival = 0.4 | α (-0.2) | -0.147 | 0.053 | -26.53% | 0.008 | 0.017 | -0.100 | 0.100 | -49.92% | 0.005 | 0.007 |
|  | β_000 (16) | 15.915 | -0.085 | -0.53% | 0.752 | 0.395 | 16.143 | 0.143 | 0.90% | 0.211 | 0.977 |
|  | β_100 (-0.8) | -0.873 | -0.073 | 9.14% | 0.127 | 0.067 | -0.085 | 0.715 | -89.42% | 0.056 | 0.146 |
|  | β_001 (1.6) | 1.367 | -0.233 | -14.59% | 0.337 | 0.825 | 1.659 | 0.059 | 3.72% | 0.297 | 1.284 |
|  | β_101 (-2) | -1.734 | 0.266 | -13.32% | 0.180 | 0.127 | -2.029 | -0.029 | 1.47% | 0.078 | 0.205 |
|  | σ^2_(u_0) (10) | 28.743 | 18.743 | 187.43% | 1.458 | 4.011 | 27.724 | 17.724 | 177.24% | NA | 3.743 |
|  | σ_u_0 u_1 (0) | -0.242 | -0.242 | NA | 0.570 | 0.166 | -2.913 | -2.913 | NA | NA | 0.582 |
|  | σ^2_(u_1 ) (10) | 10.492 | 0.492 | 4.92% | 0.471 | 0.397 | 11.477 | 1.477 | 14.77% | NA | 0.591 |
|  | σ_(v_L ) (2.2361) | 1.469 | -2.773 | -65.37% | 0.721 | 0.741 |  |  |  |  |  |
|  | σ_(v_S ) (0.6325) | 0.253 | -0.379 | -59.94% | 0.141 | 0.039 |  |  |  |  |  |
|  | σ_ε (5.7446) | 5.741 | -0.004 | -0.06% | 0.032 | 0.042 | 5.720 | -0.024 | -0.43% | NA | 0.033 |
| L3 ICC in longitudinal outcome = 0.3; variance at L2 in survival = 1 | α (-0.2) | -0.138 | 0.062 | -31.01% | 0.008 | 0.017 | -0.096 | 0.104 | -51.77% | 0.005 | 0.007 |
|  | β_000 (16) | 16.015 | 0.015 | 9.39E-04 | 0.728 | 0.362 | 16.115 | 0.115 | 0.72% | 0.210 | 0.973 |
|  | β_100 (-0.8) | -0.917 | -0.117 | 14.57% | 0.129 | 0.069 | -0.104 | 0.696 | -87.00% | 0.056 | 0.143 |
|  | β_001 (1.6) | 1.341 | -0.259 | -16.21% | 0.335 | 0.888 | 1.673 | 0.073 | 4.53% | 0.297 | 1.279 |
|  | β_101 (-2) | -1.707 | 0.293 | -14.65% | 0.181 | 0.119 | -2.033 | -0.033 | 1.67% | 0.078 | 0.207 |
|  | σ^2_(u_0) (10) | 27.930 | 17.930 | 179.30% | 1.419 | 3.887 | 27.729 | 17.729 | 177.29% | NA | 3.787 |
|  | σ_u_0 u_1 (0) | -0.274 | -0.274 | NA | 0.566 | 0.325 | -2.655 | -2.655 | NA | NA | 0.555 |
|  | σ^2_(u_1 ) (10) | 10.550 | 0.550 | 5.50% | 0.478 | 0.550 | 11.369 | 1.369 | 13.69% | NA | 0.561 |
|  | σ_(v_L ) (2.2361) | 1.495 | -2.747 | -64.75% | 0.695 | 0.737 |  |  |  |  |  |
|  | σ_(v_S ) (0.6325) | 0.324 | -0.676 | -67.58% | 0.161 | 0.126 |  |  |  |  |  |
|  | σ_ε (5.7446) | 5.738 | -0.006 | -0.11% | 0.032 | 0.040 | 5.719 | -0.025 | -0.44% | NA | 0.032 |

Table A 2‑5 Results of the proposed MJM versus standard JM censoring rate 60%, n = 100

|  |  | Proposed MJM | | | | | Standard JM | | | | |
| --- | --- | --- | --- | --- | --- | --- | --- | --- | --- | --- | --- |
|  | Parameter | Mean | Bias | RB | MESE | ESE | Mean | Bias | RB | MESE | ESE |
| L3 ICC in longitudinal outcome = 0.1; variance at L2 in survival = 0.4 | α (-0.2) | -0.142 | 0.058 | -29.08% | 0.006 | 0.004 | -0.096 | 0.104 | -52.00% | 0.003 | 0.005 |
|  | β_000 (16) | 15.788 | -0.212 | -1.33% | 0.326 | 0.239 | 16.056 | 0.056 | 0.35% | 0.126 | 0.348 |
|  | β_100 (-0.8) | -0.940 | -0.140 | 17.45% | 0.093 | 0.109 | -0.052 | 0.748 | -93.55% | 0.037 | 0.099 |
|  | β_001 (1.6) | 1.612 | 0.012 | 0.75% | 0.199 | 0.170 | 1.617 | 0.017 | 1.06% | 0.177 | 0.475 |
|  | β_101 (-2) | -1.654 | 0.346 | -17.29% | 0.128 | 0.075 | -2.008 | -0.008 | 0.38% | 0.051 | 0.144 |
|  | σ^2_(u_0) (10) | 14.683 | 4.683 | 46.83% | 0.645 | 0.468 | 14.944 | 4.944 | 49.44% | NA | 1.012 |
|  | σ_u_0 u_1 (0) | 0.003 | 0.003 | NA | 0.325 | 0.223 | -0.285 | -0.285 | NA | NA | 0.352 |
|  | σ^2_(u_1 ) (10) | 10.690 | 0.690 | 6.90% | 0.339 | 0.382 | 11.689 | 1.689 | 16.89% | NA | 0.451 |
|  | σ_(v_L ) (2.2361) | 0.813 | -1.423 | -63.64% | 0.273 | 0.113 |  |  |  |  |  |
|  | σ_(v_S ) (0.6325) | 0.297 | -0.336 | -53.10% | 0.092 | 0.068 |  |  |  |  |  |
|  | σ_ε (5.7446) | 5.738 | -0.007 | -0.12% | 0.022 | 0.018 | 5.724 | -0.020 | -0.35% | NA | 0.021 |
| L3 ICC in longitudinal outcome = 0.1; variance at L2 in survival = 1 | α (-0.2) | -0.136 | 0.064 | -31.96% | 0.005 | 0.001 | -0.092 | 0.108 | -53.91% | 0.003 | 0.005 |
|  | β_000 (16) | 15.889 | -0.111 | -0.69% | 0.295 | 0.251 | 16.061 | 0.061 | 0.38% | 0.125 | 0.340 |
|  | β_100 (-0.8) | -0.981 | -0.181 | 22.67% | 0.093 | 0.110 | -0.081 | 0.719 | -89.87% | 0.037 | 0.097 |
|  | β_001 (1.6) | 1.533 | -0.067 | -4.21% | 0.198 | 0.217 | 1.601 | 0.001 | 0.05% | 0.176 | 0.469 |
|  | β_101 (-2) | -1.639 | 0.361 | -18.07% | 0.128 | 0.069 | -1.993 | 0.007 | -0.33% | 0.051 | 0.145 |
|  | σ^2_(u_0) (10) | 14.499 | 4.499 | 44.99% | 0.636 | 0.334 | 14.873 | 4.873 | 48.73% | NA | 1.051 |
|  | σ_u_0 u_1 (0) | -0.061 | -0.061 | NA | 0.326 | 0.098 | 0.085 | 0.085 | NA | NA | 0.331 |
|  | σ^2_(u_1 ) (10) | 10.753 | 0.753 | 7.53% | 0.342 | 0.229 | 11.553 | 1.553 | 15.53% | NA | 0.450 |
|  | σ_(v_L ) (2.2361) | 0.753 | -1.483 | -66.31% | 0.244 | 0.106 |  |  |  |  |  |
|  | σ_(v_S ) (0.6325) | 0.338 | -0.662 | -66.15% | 0.099 | 0.088 |  |  |  |  |  |
|  | σ_ε (5.7446) | 5.737 | -0.008 | -0.13% | 0.022 | 0.021 | 5.724 | -0.020 | -0.36% | NA | 0.021 |
| L3 ICC in longitudinal outcome = 0.3; variance at L2 in survival = 0.4 | α (-0.2) | -0.144 | 0.056 | -27.79% | 0.006 | 0.004 | -0.100 | 0.100 | -50.12% | 0.003 | 0.005 |
|  | β_000 (16) | 15.569 | -0.431 | -2.70% | 0.489 | 0.371 | 16.104 | 0.104 | 0.65% | 0.150 | 0.615 |
|  | β_100 (-0.8) | -0.949 | -0.149 | 18.67% | 0.093 | 0.088 | -0.080 | 0.720 | -89.95% | 0.039 | 0.100 |
|  | β_001 (1.6) | 1.740 | 0.140 | 8.75% | 0.243 | 0.338 | 1.667 | 0.067 | 4.17% | 0.210 | 0.867 |
|  | β_101 (-2) | -1.652 | 0.348 | -17.38% | 0.128 | 0.030 | -2.007 | -0.007 | 0.34% | 0.055 | 0.145 |
|  | σ^2_(u_0) (10) | 25.561 | 15.561 | 155.61% | 0.944 | 0.913 | 28.366 | 18.366 | 183.66% | NA | 2.972 |
|  | σ_u_0 u_1 (0) | -0.003 | -0.003 | NA | 0.391 | 0.343 | -1.217 | -1.217 | NA | NA | 0.421 |
|  | σ^2_(u_1 ) (10) | 10.745 | 0.745 | 7.45% | 0.341 | 0.376 | 11.467 | 1.467 | 14.67% | NA | 0.420 |
|  | σ_(v_L ) (2.2361) | 1.411 | -2.832 | -66.74% | 0.398 | 0.146 |  |  |  |  |  |
|  | σ_(v_S ) (0.6325) | 0.297 | -0.335 | -53.02% | 0.092 | 0.055 |  |  |  |  |  |
|  | σ_ε (5.7446) | 5.737 | -0.007 | -0.13% | 0.022 | 0.017 | 5.721 | -0.024 | -0.41% | NA | 0.021 |
| L3 ICC in longitudinal outcome = 0.3; variance at L2 in survival = 1 | α (-0.2) | -0.134 | 0.066 | -33.09% | 0.005 | 0.003 | -0.095 | 0.105 | -52.49% | 0.003 | 0.005 |
|  | β_000 (16) | 15.667 | -0.333 | -2.08% | 0.508 | 0.464 | 16.139 | 0.139 | 0.87% | 0.149 | 0.631 |
|  | β_100 (-0.8) | -0.942 | -0.142 | 17.78% | 0.094 | 0.138 | -0.107 | 0.693 | -86.68% | 0.039 | 0.097 |
|  | β_001 (1.6) | 1.629 | 0.029 | 1.80% | 0.242 | 0.377 | 1.590 | -0.010 | -0.66% | 0.210 | 0.862 |
|  | β_101 (-2) | -1.660 | 0.340 | -16.99% | 0.128 | 0.074 | -1.991 | 0.009 | -0.46% | 0.055 | 0.142 |
|  | σ^2_(u_0) (10) | 26.003 | 16.003 | 160.03% | 0.957 | 0.728 | 28.182 | 18.182 | 181.82% | NA | 2.919 |
|  | σ_u_0 u_1 (0) | -0.305 | -0.305 | NA | 0.398 | 0.381 | -1.380 | -1.380 | NA | NA | 0.439 |
|  | σ^2_(u_1 ) (10) | 10.721 | 0.721 | 7.21% | 0.342 | 0.388 | 11.362 | 1.362 | 13.62% | NA | 0.431 |
|  | σ_(v_L ) (2.2361) | 1.449 | -2.794 | -65.85% | 0.413 | 0.062 |  |  |  |  |  |
|  | σ_(v_S ) (0.6325) | 0.333 | -0.667 | -66.68% | 0.098 | 0.089 |  |  |  |  |  |
|  | σ_ε (5.7446) | 5.731 | -0.013 | -0.23% | 0.022 | 0.021 | 5.723 | -0.022 | -0.38% | NA | 0.022 |

Table A 2‑6 Results of the proposed MJM versus standard JM censoring rate 60%, n = 200

|  |  | Proposed MJM | | | | | Standard JM | | | | |
| --- | --- | --- | --- | --- | --- | --- | --- | --- | --- | --- | --- |
|  | Parameter | Mean | Bias | RB | MESE | ESE | Mean | Bias | RB | MESE | ESE |
| L3 ICC in longitudinal outcome = 0.1; variance at L2 in survival = 0.4 | α (-0.2) | -0.138 | 0.062 | -31.06% | 0.004 | 0.006 | -0.097 | 0.103 | -51.32% | 0.002 | 0.003 |
|  | β_000 (16) | 15.809 | -0.191 | -1.19% | 0.253 | 0.202 | 16.108 | 0.108 | 0.67% | 0.088 | 0.239 |
|  | β_100 (-0.8) | -1.024 | -0.224 | 28.04% | 0.067 | 0.119 | -0.013 | 0.787 | -98.40% | 0.025 | 0.064 |
|  | β_001 (1.6) | 1.773 | 0.173 | 10.81% | 0.137 | 0.387 | 1.599 | -0.001 | -0.06% | 0.124 | 0.346 |
|  | β_101 (-2) | -1.581 | 0.419 | -20.94% | 0.089 | 0.062 | -2.006 | -0.006 | 0.29% | 0.036 | 0.096 |
|  | σ^2_(u_0) (10) | 14.945 | 4.945 | 49.45% | 0.463 | 0.777 | 14.953 | 4.953 | 49.53% | NA | 0.749 |
|  | σ_u_0 u_1 (0) | 0.064 | 0.064 | NA | 0.231 | 0.137 | 0.690 | 0.690 | NA | NA | 0.269 |
|  | σ^2_(u_1 ) (10) | 10.590 | 0.590 | 5.90% | 0.240 | 0.259 | 11.666 | 1.666 | 16.66% | NA | 0.331 |
|  | σ_(v_L ) (2.2361) | 0.664 | -1.572 | -70.31% | 0.211 | 0.099 |  |  |  |  |  |
|  | σ_(v_S ) (0.6325) | 0.216 | -0.417 | -65.88% | 0.052 | 0.017 |  |  |  |  |  |
|  | σ_ε (5.7446) | 5.735 | -0.010 | -0.17% | 0.016 | 0.007 | 5.722 | -0.022 | -0.38% | NA | 0.015 |
| L3 ICC in longitudinal outcome = 0.1; variance at L2 in survival = 1 | α (-0.2) | -0.129 | 0.071 | -35.39% | 0.004 | 0.004 | -0.093 | 0.107 | -53.35% | 0.002 | 0.003 |
|  | β_000 (16) | 15.814 | -0.186 | -1.17% | 0.237 | 0.273 | 16.101 | 0.101 | 0.63% | 0.088 | 0.239 |
|  | β_100 (-0.8) | -0.998 | -0.198 | 24.74% | 0.066 | 0.118 | -0.045 | 0.755 | -94.43% | 0.025 | 0.066 |
|  | β_001 (1.6) | 1.764 | 0.164 | 10.27% | 0.136 | 0.456 | 1.587 | -0.013 | -0.83% | 0.124 | 0.343 |
|  | β_101 (-2) | -1.611 | 0.389 | -19.45% | 0.089 | 0.078 | -1.998 | 0.002 | -0.12% | 0.036 | 0.098 |
|  | σ^2_(u_0) (10) | 14.715 | 4.715 | 47.15% | 0.453 | 0.868 | 14.927 | 4.927 | 49.27% | NA | 0.677 |
|  | σ_u_0 u_1 (0) | 0.093 | 0.093 | NA | 0.231 | 0.107 | 0.675 | 0.675 | NA | NA | 0.261 |
|  | σ^2_(u_1 ) (10) | 10.503 | 0.503 | 5.03% | 0.241 | 0.232 | 11.580 | 1.580 | 15.80% | NA | 0.326 |
|  | σ_(v_L ) (2.2361) | 0.717 | -1.519 | -67.94% | 0.188 | 0.063 |  |  |  |  |  |
|  | σ_(v_S ) (0.6325) | 0.258 | -0.742 | -74.22% | 0.056 | 0.024 |  |  |  |  |  |
|  | σ_ε (5.7446) | 5.738 | -0.006 | -0.11% | 0.016 | 0.006 | 5.723 | -0.022 | -0.38% | NA | 0.015 |
| L3 ICC in longitudinal outcome = 0.3; variance at L2 in survival = 0.4 | α (-0.2) | -0.141 | 0.059 | -29.33% | 0.004 | 0.005 | -0.100 | 0.100 | -50.11% | 0.003 | 0.004 |
|  | β_000 (16) | 15.640 | -0.360 | -2.25% | 0.331 | 0.380 | 16.222 | 0.222 | 1.39% | 0.105 | 0.431 |
|  | β_100 (-0.8) | -0.998 | -0.198 | 24.77% | 0.066 | 0.116 | -0.047 | 0.753 | -94.13% | 0.027 | 0.062 |
|  | β_001 (1.6) | 1.978 | 0.378 | 23.62% | 0.166 | 0.641 | 1.573 | -0.027 | -1.69% | 0.148 | 0.600 |
|  | β_101 (-2) | -1.606 | 0.394 | -19.68% | 0.090 | 0.083 | -2.001 | -0.001 | 0.05% | 0.038 | 0.094 |
|  | σ^2_(u_0) (10) | 26.485 | 16.485 | 164.85% | 0.686 | 1.406 | 28.407 | 18.407 | 184.07% | NA | 1.866 |
|  | σ_u_0 u_1 (0) | -0.059 | -0.059 | NA | 0.278 | 0.410 | 0.069 | 0.069 | NA | NA | 0.325 |
|  | σ^2_(u_1 ) (10) | 10.517 | 0.517 | 5.17% | 0.238 | 0.226 | 11.523 | 1.523 | 15.23% | NA | 0.327 |
|  | σ_(v_L ) (2.2361) | 1.345 | -2.897 | -68.29% | 0.253 | 0.134 |  |  |  |  |  |
|  | σ_(v_S ) (0.6325) | 0.234 | -0.398 | -62.96% | 0.055 | 0.020 |  |  |  |  |  |
|  | σ_ε (5.7446) | 5.738 | -0.006 | -0.11% | 0.016 | 0.004 | 5.720 | -0.025 | -0.43% | NA | 0.016 |
| L3 ICC in longitudinal outcome = 0.3; variance at L2 in survival = 1 | α (-0.2) | -0.133 | 0.067 | -33.59% | 0.004 | 0.002 | -0.096 | 0.104 | -52.13% | 0.002 | 0.004 |
|  | β_000 (16) | 15.696 | -0.304 | -1.90% | 0.330 | 0.407 | 16.243 | 0.243 | 1.52% | 0.105 | 0.446 |
|  | β_100 (-0.8) | -1.014 | -0.214 | 26.77% | 0.067 | 0.118 | -0.072 | 0.728 | -91.04% | 0.027 | 0.063 |
|  | β_001 (1.6) | 1.881 | 0.281 | 17.58% | 0.167 | 0.686 | 1.537 | -0.063 | -3.92% | 0.148 | 0.637 |
|  | β_101 (-2) | -1.615 | 0.385 | -19.23% | 0.090 | 0.084 | -1.992 | 0.008 | -0.41% | 0.038 | 0.096 |
|  | σ^2_(u_0) (10) | 26.634 | 16.634 | 166.34% | 0.687 | 1.810 | 28.407 | 18.407 | 184.07% | NA | 1.977 |
|  | σ_u_0 u_1 (0) | -0.128 | -0.128 | NA | 0.281 | 0.147 | 0.504 | 0.504 | NA | NA | 0.321 |
|  | σ^2_(u_1 ) (10) | 10.645 | 0.645 | 6.45% | 0.242 | 0.185 | 11.408 | 1.408 | 14.08% | NA | 0.316 |
|  | σ_(v_L ) (2.2361) | 1.276 | -2.967 | -69.93% | 0.254 | 0.114 |  |  |  |  |  |
|  | σ_(v_S ) (0.6325) | 0.279 | -0.721 | -72.09% | 0.060 | 0.041 |  |  |  |  |  |
|  | σ_ε (5.7446) | 5.738 | -0.006 | -0.11% | 0.016 | 0.004 | 5.720 | -0.025 | -0.43% | NA | 0.014 |

# Appendix 3 Trace plots of Raw MCMC chains for the MJM with a linear trajectory for the real study (PAX)

Note: Alpha indicates the association parameter between the longitudinal and survival outcome. BetaL indicate parameters in the longitudinal submodel: betaL[1] – intercept, betaL[2] – time, betaL[3] – PAX, betaL[4] – interaction of time and PAX, betaL[5] – sex, betaL[6] – interaction of sex and time, betaL[7] – students SEFI, betaL[8] – urban school, betal[9] – mother’s maternal mental disorder diagnosis, betaL[10] – income assistant (IA), betaL[11] – interaction of time and IA, betaL[12] – Child in Care (CIC) service, betaL[13] – interaction of time and CIC, betaL[14] – Child and Family Service (CFS), betaL[15] – interaction of time and CFS. Gamma indicates parameters in the survival submodel: gamma[1] – PAX, gamma[2] – sex, gamma[3] – students SEFI, gamma[4] – urban school, gamma[5] – mother’s maternal mental disorder diagnosis, gamma[6] – IA, gamma[7] – CIC, gamma[8] – CFS. Sigma indicate the standard devitaion of level-1 residual, sigma.vL indicates the standard deviation of random effects at the group level in the longitudinal submodel, sigma.vS indicates the standard deviation of random effects at the group level in the survival submodel. Sigma.uL indicates the variance-covaraince matrix of the random effects at the individual level in the longitudinal submodel: Sigma.uL[1,1] – the variance of random intercepts at the individual level , Sigma.uL[1,2] – covariance of random intercepts and slopes at the individual level, Sigma.uL[2,2] – variance of random slopes at the individual level.

# Appendix 4 Autocorrelation plots of raw MCMC chains for the MJM with a linear trajectory for the real study (PAX)

Note: Alpha indicates the association parameter between the longitudinal and survival outcome. BetaL indicate parameters in the longitudinal submodel: betaL[1] – intercept, betaL[2] – time, betaL[3] – PAX, betaL[4] – interaction of time and PAX, betaL[5] – sex, betaL[6] – interaction of sex and time, betaL[7] – students SEFI, betaL[8] – urban school, betal[9] – mother’s maternal mental disorder diagnosis, betaL[10] – income assistant (IA), betaL[11] – interaction of time and IA, betaL[12] – Child in Care (CIC) service, betaL[13] – interaction of time and CIC, betaL[14] – Child and Family Service (CFS), betaL[15] – interaction of time and CFS. Gamma indicates parameters in the survival submodel: gamma[1] – PAX, gamma[2] – sex, gamma[3] – students SEFI, gamma[4] – urban school, gamma[5] – mother’s maternal mental disorder diagnosis, gamma[6] – IA, gamma[7] – CIC, gamma[8] – CFS. Sigma indicate the standard devitaion of level-1 residual, sigma.vL indicates the standard deviation of random effects at the group level in the longitudinal submodel, sigma.vS indicates the standard deviation of random effects at the group level in the survival submodel. Sigma.uL indicates the variance-covaraince matrix of the random effects at the individual level in the longitudinal submodel: Sigma.uL[1,1] – the variance of random intercepts at the individual level , Sigma.uL[1,2] – covariance of random intercepts and slopes at the individual level, Sigma.uL[2,2] – variance of random slopes at the individual level.

# Appendix 5 Autocorrelation plots of thinned MCMC chains after burn-in for the MJM 1 with a linear trajectory for the real study (PAX)

# Appendix 6 Trace plots of thinned MCMC chains after burn-in for the three MJMs for the real study (PAX)

## MJM1: linear trajectory


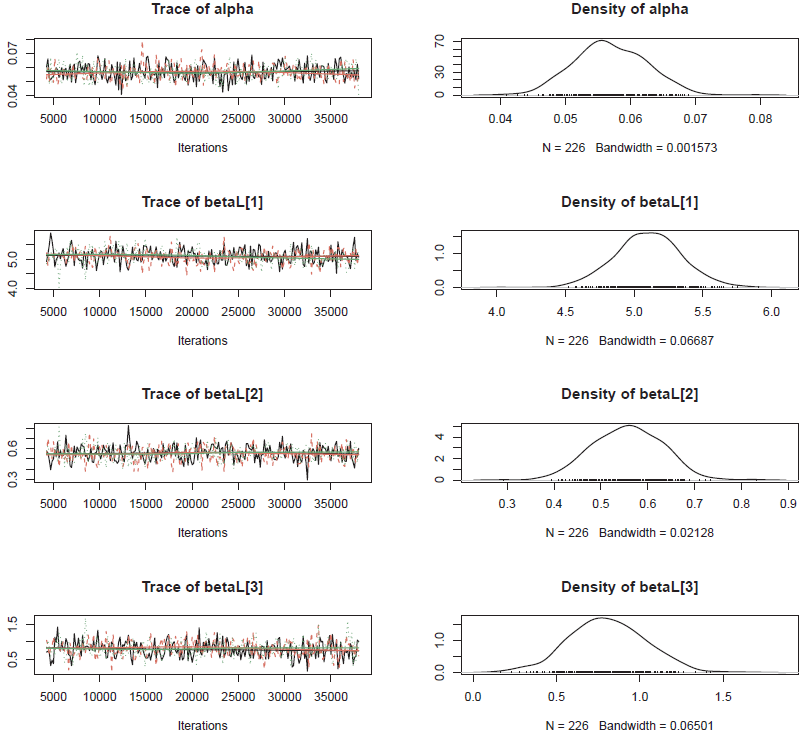


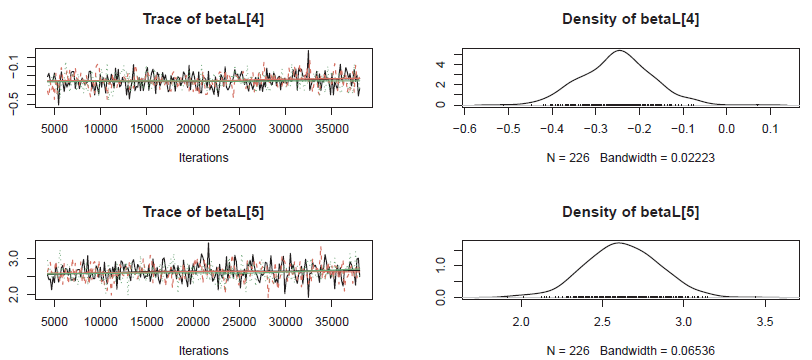


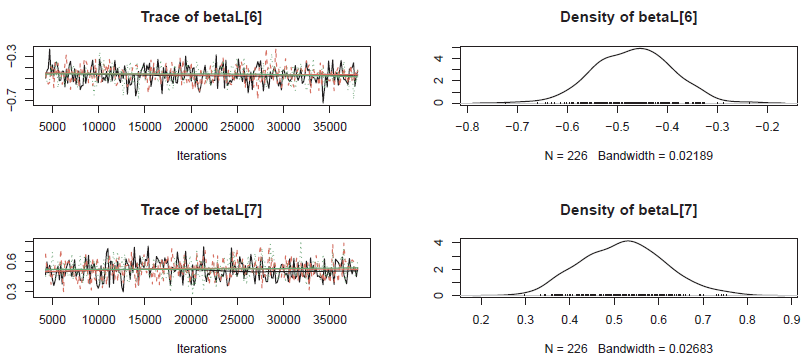


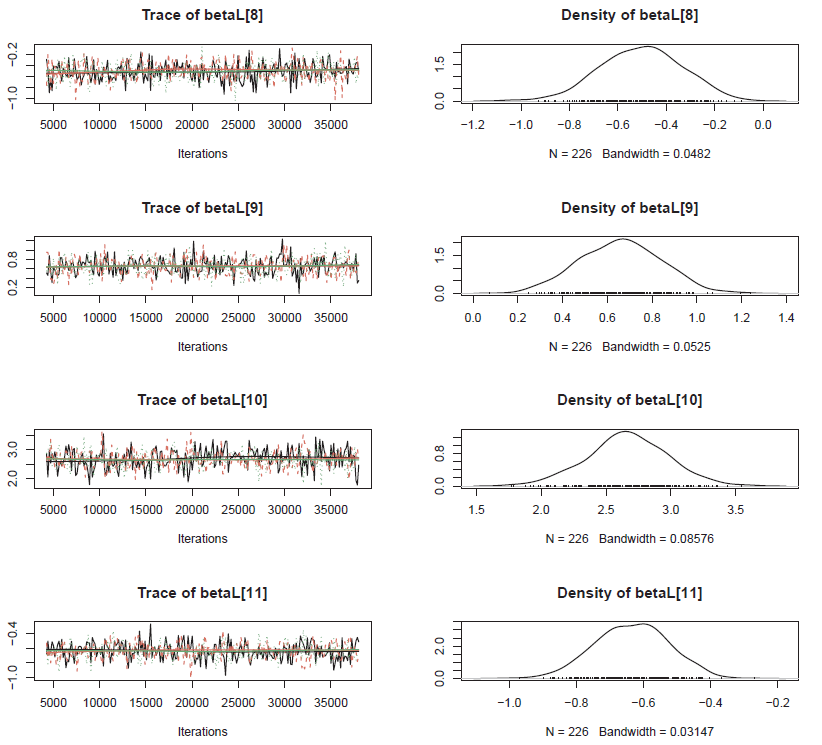


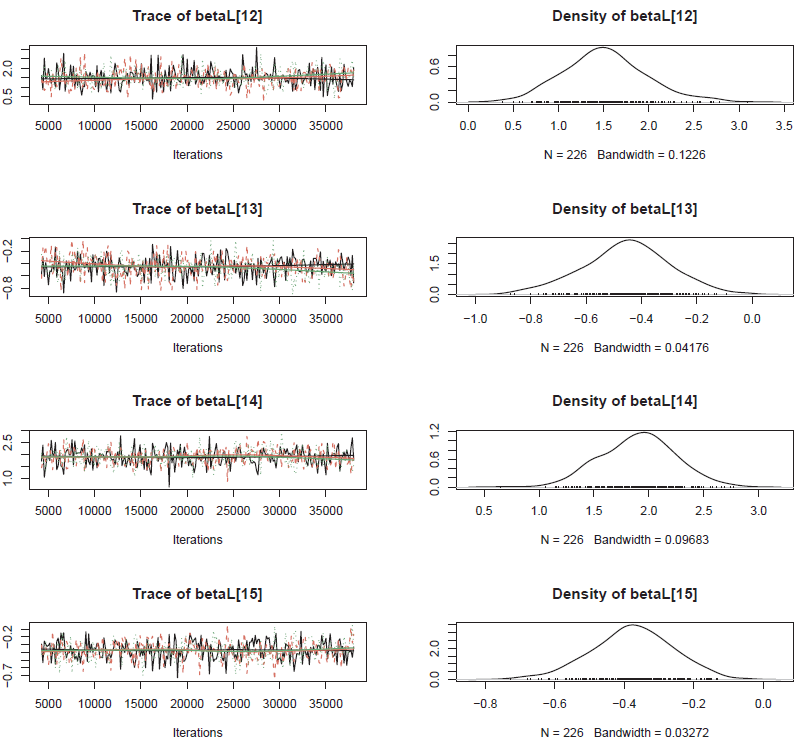


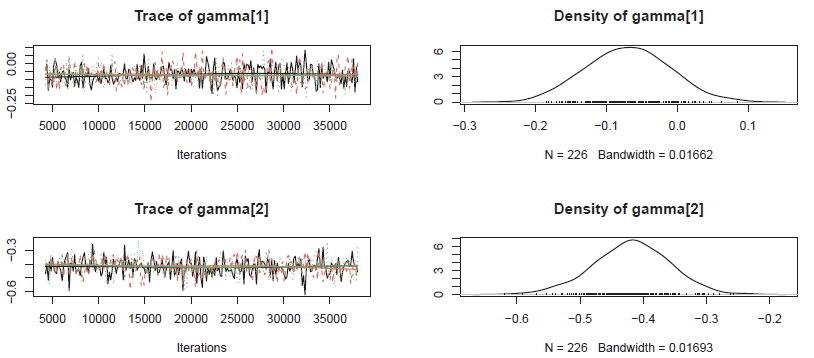


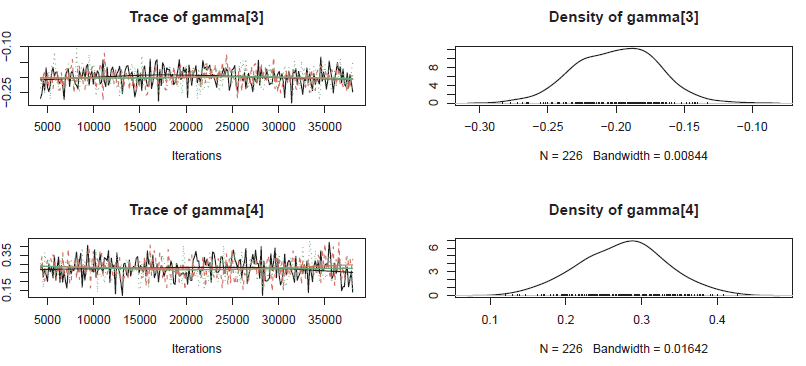

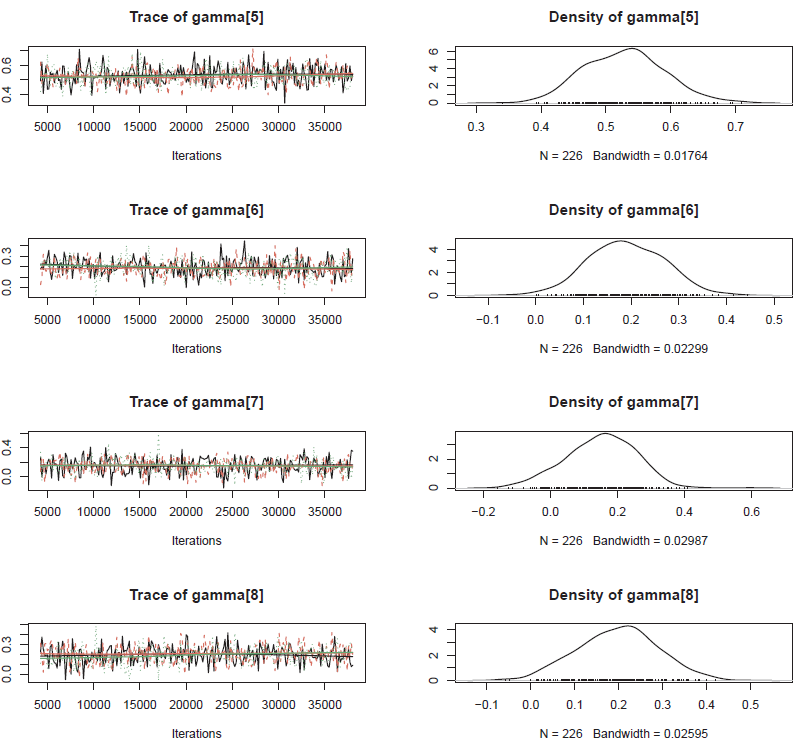

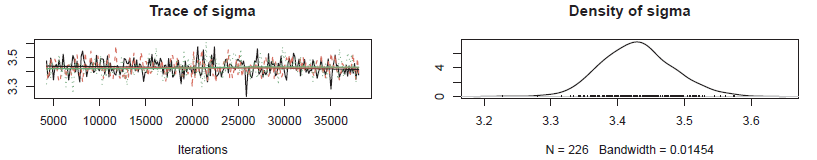

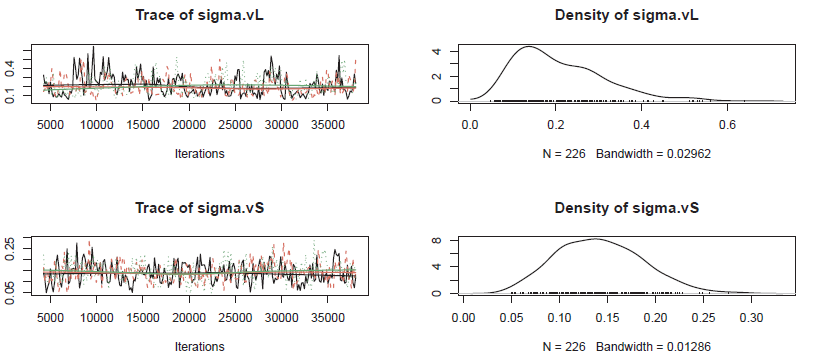


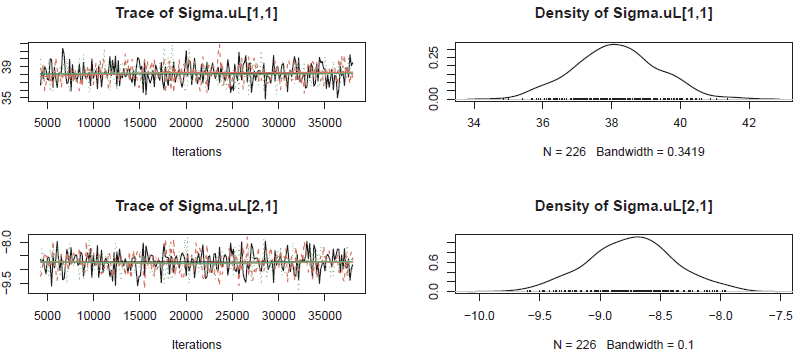

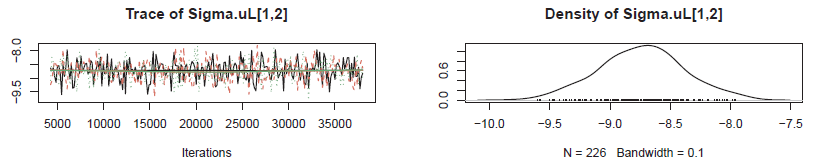

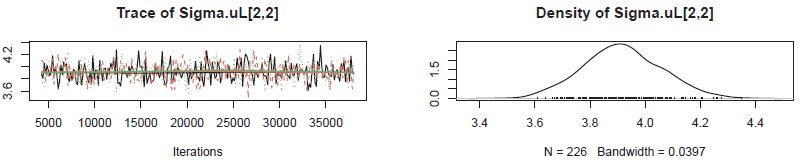


Note: Alpha indicates the association parameter between the longitudinal and survival outcome. BetaL indicate parameters in the longitudinal submodel: betaL[1] – intercept, betaL[2] – time, betaL[3] – PAX, betaL[4] – interaction of time and PAX, betaL[5] – sex, betaL[6] – interaction of sex and time, betaL[7] – students SEFI, betaL[8] – urban school, betal[9] – mother’s maternal mental disorder diagnosis, betaL[10] – income assistant (IA), betaL[11] – interaction of time and IA, betaL[12] – Child in Care (CIC) service, betaL[13] – interaction of time and CIC, betaL[14] – Child and Family Service (CFS), betaL[15] – interaction of time and CFS. Gamma indicates parameters in the survival submodel: gamma[1] – PAX, gamma[2] – sex, gamma[3] – students SEFI, gamma[4] – urban school, gamma[5] – mother’s maternal mental disorder diagnosis, gamma[6] – IA, gamma[7] – CIC, gamma[8] – CFS. Sigma indicate the standard devitaion of level-1 residual, sigma.vL indicates the standard deviation of random effects at the group level in the longitudinal submodel, sigma.vS indicates the standard deviation of random effects at the group level in the survival submodel. Sigma.uL indicates the variance-covaraince matrix of the random effects at the individual level in the longitudinal submodel: Sigma.uL[1,1] – the variance of random intercepts at the individual level , Sigma.uL[1,2] – covariance of random intercepts and slopes at the individual level, Sigma.uL[2,2] – variance of random slopes at the individual level.

## MJM2: quadratic trajectory, slope of time varies by PAX

Note: Alpha indicates the association parameter between the longitudinal and survival outcome. BetaL indicate parameters in the longitudinal submodel: betaL[1] – intercept, betaL[2] – time, betaL[3] – time^2^, betaL[4] – PAX, betaL[5] – interaction of time and PAX, betaL[6] – sex, betaL[7] – interaction of sex and time, betaL[8] – students SEFI, betaL[9] – urban school, betal[10] – mother’s maternal mental disorder diagnosis, betaL[11] – income assistant (IA), betaL[12] – interaction of time and IA, betaL[13] – Child in Care (CIC) service, betaL[14] – interaction of time and CIC, betaL[15] – Child and Family Service (CFS), betaL[16] – interaction of time and CFS. Gamma indicates parameters in the survival submodel: gamma[1] – PAX, gamma[2] – sex, gamma[3] – students SEFI, gamma[4] – urban school, gamma[5] – mother’s maternal mental disorder diagnosis, gamma[6] – IA, gamma[7] – CIC, gamma[8] – CFS. Sigma indicate the standard devitaion of level-1 residual, sigma.vL indicates the standard deviation of random effects at the group level in the longitudinal submodel, sigma.vS indicates the standard deviation of random effects at the group level in the survival submodel. Sigma.uL indicates the variance-covaraince matrix of the random effects at the individual level in the longitudinal submodel: Sigma.uL[1,1] – the variance of the random intercepts at the individual level , Sigma.uL[1,2] – covariance of random intercepts and slopes at the individual level, Sigma.uL[2,2] – variance of random slopes at the individual level.

## MJM3: quadratic trajectory, slope of time and slope of time^2^ varies by PAX

Note: Alpha indicates the association parameter between the longitudinal and survival outcome. BetaL indicate parameters in the longitudinal submodel: betaL[1] – intercept, betaL[2] – time, betaL[3] – time^2^, betaL[4] – PAX, betaL[5] – interaction of time and PAX, betaL[6] – interaction of time^2^ and PAX, betaL[7] – sex, betaL[8] – interaction of sex and time, betaL[9] – students SEFI, betaL[10] – urban school, betal[11] – mother’s maternal mental disorder diagnosis, betaL[12] – income assistant (IA), betaL[13] – interaction of time and IA, betaL[14] – Child in Care (CIC) service, betaL[15] – interaction of time and CIC, betaL[16] – Child and Family Service (CFS), betaL[17] – interaction of time and CFS. Gamma indicates parameters in the survival submodel: gamma[1] – PAX, gamma[2] – sex, gamma[3] – students SEFI, gamma[4] – urban school, gamma[5] – mother’s maternal mental disorder diagnosis, gamma[6] – IA, gamma[7] – CIC, gamma[8] – CFS. Sigma indicate the standard devitaion of level-1 residual, sigma.vL indicates the standard deviation of random effects at the group level in the longitudinal submodel, sigma.vS indicates the standard deviation of random effects at the group level in the survival submodel. Sigma.uL indicates the variance-covaraince matrix of the random effects at the individual level in the longitudinal submodel: Sigma.uL[1,1] – the variance of the random intercepts at the individual level , Sigma.uL[1,2] – covariance of random intercepts and slopes at the individual level, Sigma.uL[2,2] – variance of random slopes at the individual level.

# Appendix 7 Gelman-Rubin-Brooks diagnostics for all the three MJMs for the real study (PAX)

|  |  |  | MJM1 | MJM2 | MJM3 |
| --- | --- | --- | --- | --- | --- |
| Survival | Fixed effects | PAX | 1.04 | 1.01 | 1.01 |
|  |  | Sex M | 1.03 | 1.01 | 1.00 |
|  |  | student_sefi | 1.00 | 1.01 | 1.02 |
|  |  | Urbrha | 1.00 | 1.00 | 1.00 |
|  |  | ever on IA | 1.02 | 1.00 | 1.00 |
|  |  | ever in CIC | 1.00 | 1.00 | 1.00 |
|  |  | ever in CFS | 1.01 | 1.01 | 1.00 |
|  |  | maternal mental disorder | 1.00 | 1.00 | 1.02 |
|  |  | Association | 1.01 | 1.00 | 1.00 |
|  | Random effects | frailty (SD) | 1.01 | 1.01 | 1.00 |
|  |  |  |  |  |  |
| Longitudinal | Fixed effect | Intercept | 1.01 | 1.02 | 1.00 |
|  |  | time | 1.02 | 1.04 | 1.01 |
|  |  | time*time |  | 1.04 | 1.01 |
|  |  | PAX | 1.00 | 1.03 | 1.01 |
|  |  | Sex M | 1.00 | 1.01 | 1.00 |
|  |  | student_sefi | 1.00 | 1.00 | 1.01 |
|  |  | Urbrha | 1.01 | 1.00 | 1.03 |
|  |  | ever on IA | 1.02 | 1.01 | 1.02 |
|  |  | ever in CIC | 1.01 | 1.01 | 1.01 |
|  |  | ever in CFS | 1.03 | 1.00 | 1.01 |
|  |  | maternal mental disorder | 1.00 | 1.01 | 1.06 |
|  |  | time*PAX | 1.03 | 1.02 | 1.01 |
|  |  | time*time*PAX |  |  | 1.01 |
|  |  | time*sexM | 1.00 | 1.01 | 1.00 |
|  |  | time*IA | 1.01 | 1.00 | 1.02 |
|  |  | time*CIC | 1.01 | 1.00 | 1.01 |
|  |  | time*CFS | 1.04 | 1.00 | 1.00 |
|  | Random effects | intercept at individual level (variance) | 1.01 | 1.00 | 1.01 |
|  |  | slope at individual level (variance) | 1.00 |  |  |
|  |  | covariance of random intercept and slope | 1.00 |  |  |
|  |  | residual (SD) | 1.00 | 1.01 | 1.01 |
|  |  | intercept at group level (SD) | 1.11 | 1.01 | 1.03 |

# Appendix 8 Results of sensitivity analysis before and after thinning for MJM 1 with linear trajectory for the real study (PAX)

|  |  |  | MJM 1 (thinned) | | | | MJM 1 (without thinned) | | | |
| --- | --- | --- | --- | --- | --- | --- | --- | --- | --- | --- |
| Parameters | | | estimate | SD | Q2.5 | Q97.5 | estimate | SD | Q2.5 | Q97.5 |
| Survival | Fixed effects | PAX | -0.064 | 0.061 | -0.182 | 0.046 | -0.063 | 0.060 | -0.181 | 0.054 |
|  |  | Sex M | -0.461 | 0.061 | -0.586 | -0.335 | -0.461 | 0.062 | -0.583 | -0.339 |
|  |  | Student Sefi | -0.206 | 0.032 | -0.269 | -0.187 | -0.206 | 0.031 | -0.268 | -0.146 |
|  |  | Urban School | 0.264 | 0.061 | 0.146 | 0.387 | 0.267 | 0.061 | 0.148 | 0.387 |
|  |  | ever on IA | 0.191 | 0.079 | 0.044 | 0.334 | 0.191 | 0.080 | 0.032 | 0.348 |
|  |  | ever in CIC | 0.279 | 0.103 | 0.076 | 0.476 | 0.278 | 0.107 | 0.068 | 0.486 |
|  |  | ever in CFS | 0.187 | 0.093 | -0.010 | 0.360 | 0.187 | 0.093 | 0.004 | 0.367 |
|  |  | maternal mental disorder | 0.569 | 0.062 | 0.445 | 0.688 | 0.568 | 0.062 | 0.445 | 0.690 |
|  |  | Association | 0.056 | 0.006 | 0.045 | 0.066 | 0.056 | 0.006 | 0.045 | 0.067 |
|  | Random effects | frailty (SD) | 0.142 | 0.047 | 0.059 | 0.217 | 0.123 | 0.041 | 0.059 | 0.215 |
|  |  |  |  |  |  |  |  |  |  |  |
| Longitudinal | Fixed effect | Intercept | 5.135 | 0.236 | 4.655 | 5.566 | 5.158 | 0.249 | 4.674 | 5.650 |
|  |  | time | 0.552 | 0.078 | 0.383 | 0.705 | 0.553 | 0.078 | 0.398 | 0.705 |
|  |  | PAX | 0.769 | 0.247 | 0.282 | 1.264 | 0.744 | 0.246 | 0.248 | 1.210 |
|  |  | Sex M | 2.301 | 0.229 | 1.830 | 2.712 | 2.300 | 0.232 | 1.828 | 2.742 |
|  |  | Student Sefi | 0.522 | 0.092 | 0.336 | 0.702 | 0.525 | 0.089 | 0.350 | 0.700 |
|  |  | Urban School | -0.578 | 0.183 | -0.927 | -0.222 | -0.580 | 0.178 | -0.921 | -0.229 |
|  |  | ever on IA | 2.695 | 0.321 | 2.090 | 3.294 | 2.703 | 0.307 | 2.101 | 3.303 |
|  |  | ever in CIC | 1.399 | 0.484 | 0.401 | 2.303 | 1.390 | 0.468 | 0.450 | 2.274 |
|  |  | ever in CFS | 1.570 | 0.370 | 0.795 | 2.277 | 1.577 | 0.353 | 0.897 | 2.267 |
|  |  | maternal mental disorder | 0.561 | 0.177 | 0.230 | 0.899 | 0.559 | 0.184 | 0.198 | 0.916 |
|  |  | time*PAX | -0.249 | 0.080 | -0.401 | -0.096 | -0.249 | 0.084 | -0.409 | -0.082 |
|  |  | time*sexM | -0.387 | 0.080 | -0.512 | -0.234 | -0.389 | 0.081 | -0.542 | -0.226 |
|  |  | time*IA | -0.626 | 0.110 | -0.845 | -0.404 | -0.633 | 0.109 | -0.770 | -0.100 |
|  |  | time*CIC | -0.439 | 0.166 | -0.747 | -0.109 | -0.435 | 0.170 | -0.564 | -0.067 |
|  |  | time*CFS | -0.317 | 0.135 | -0.585 | -0.072 | -0.315 | 0.128 | -2.808 | 0.005 |
|  | Random effects | intercept at individual level (variance) | 36.045 | 1.188 | 33.988 | 38.694 | 35.963 | 1.199 | 33.819 | 38.212 |
|  |  | time (variance) | 3.852 | 0.149 | 3.580 | 4.167 | 3.852 | 0.147 | 3.575 | 4.148 |
|  |  | intercept*time (covariance) | -8.310 | 0.367 | -9.112 | -7.675 | -8.304 | 0.358 | -9.021 | -7.622 |
|  |  | residual (SD) | 3.356 | 0.055 | 3.253 | 3.464 | 3.358 | 0.055 | 3.253 | 3.468 |
|  |  | intercept at group level (SD) | 0.241 | 0.134 | 0.074 | 0.561 | 0.223 | 0.119 | 0.069 | 0.507 |

# Appendix 9 Running time of the MJMs for the real study (PAX)

| Model | running time (hours) |
| --- | --- |
| MJM 1 | 66.73 |
| MJM 2 | 83.33 |
| MJM 3 | 81.56 |

# Appendix 10 Trace plots of MCMC chains for a random replicate in the simulation studies for the proposed MJM

Note: Alpha indicates the association parameter between the longitudinal and survival outcome. BetaL indicate parameters in the longitudinal submodel: betaL[1] – intercept, betaL[2] – time, betaL[3] – PAX, betaL[4] – interaction of time and PAX. Sigma indicate the standard devitaion of level-1 residual, sigma.vL indicates the standard deviation of random effects at the group level in the longitudinal submodel, sigma.vS indicates the standard deviation of random effects at the group level in the survival submodel. Sigma.uL indicates the variance-covaraince matrix of the random effects at the individual level in the longitudinal submodel: Sigma.uL[1,1] – the variance of random intercepts at the individual level , Sigma.uL[1,2] – covariance of the random intercepts and slopes at the individual level, Sigma.uL[2,2] – variance of random slopes at the individual level.
